# Identification of the laccase-like multicopper oxidase gene family of sweet cherry (*Prunus avium* L.) and expression analysis in six ancient Tuscan varieties

**DOI:** 10.1038/s41598-019-39151-z

**Published:** 2019-03-05

**Authors:** Roberto Berni, Emilie Piasecki, Sylvain Legay, Jean-Francois Hausman, Khawar Sohail Siddiqui, Giampiero Cai, Gea Guerriero

**Affiliations:** 1 Department of Life Sciences, University of Siena, via P.A. Mattioli 4, 53100 Siena, Italy; 2 Trees and timber institute-National research council of Italy (CNR-IVALSA), via Aurelia 49, 58022 Follonica, GR Italy; 3 Research and Innovation Department, Luxembourg Institute of Science and Technology, 5 avenue des Hauts-Fourneaux, L-4362 Esch/Alzette, Luxembourg; 4 Life Sciences Department, King Fahd University of Petroleum and Minerals (KFUPM), 31261 Dhahran, Saudi Arabia

## Abstract

Laccase-like multicopper oxidases (LMCOs) are versatile enzymes used as biocatalysts performing the oxidation of different substrates of industrial relevance, with or without the intervention of a mediator. They have attracted a lot of interest for biotechnological applications in light of their eco-friendliness: they indeed oxidize the substrate(s) by coupling the four electron reduction of the final acceptor, molecular oxygen (O_2_), to water. Plant LMCOs represent a still poorly studied, important class of oxidoreductases controlling e.g. the post-harvest quality of fruits and enabling the tailoring of designer energy crops. We here sought to identify the LMCOs in *Prunus avium* L., whose fruits are rich in bioactive molecules, but are also highly perishable. The goal was to analyze them using bioinformatics (phylogenetic and *in silico* structural analyses) and to perform a targeted expression study on a subset of genes in six ancient varieties from Tuscany, all threatened by genetic erosion. These sweet cherry varieties contain higher amount of bioactive molecules, as compared to commercial counterparts. The results shown demonstrate strikingly different gene expression patterns in the six ancient varieties (‘Benedetta’, ‘Carlotta’, ‘Crognola’, ‘Maggiola’, ‘Morellona’, ‘Moscatella’) belonging to the Tuscan Regional Bank of Germplasm, as compared to a widely used commercial one (‘Durone’). The motivation of this study is the economic importance of *P. avium* and the involvement of LMCOs in post-harvest fruit parameters, like color. The results presented pave the way to follow-up researches on LMCOs of sweet cherry exploring post-harvest fruit parameters (e.g. anthocyanin stability responsible for pericarp browning and the preservation of the appealing red color), as well as developmental processes, like stony pit formation.

## Introduction

Laccase-like multicopper oxidases (LMCOs; EC 1.10.3.2) belong to the MCO superfamily and constitute a multigenic family of oxidoreductases that is distributed across bacteria, fungi and plants^1^. Often referred to simply as “laccases” (we will however avoid here the term laccase, which should be used for those enzymes acting on urushiol^1^), MCOs can oxidize a panoply of substrates, many of which are of special industrial interest^2^. The substrate promiscuity of LMCOs is dictated by the redox potential at one of their 4 Cu atoms, i.e. the type 1 catalytic Cu atom (T1 Cu), which is where substrate oxidation takes place^3^. Low-redox potential LMCOs are typically found in bacteria and plants, medium-redox potential ones are produced by fungi, while high-redox potential enzymes are secreted by white-rot basidiomycetes^3^. These latter LMCOs have broader substrate specificities and are therefore mainly used for biotechnological purposes. Fungal laccases are indeed the best studied enzymes showing the broadest industrial applications spanning food processing, biofuel production, textile, pharmaceutical, nanobiotechnology, bioremediation and synthetic chemistry including polymer fabrication^2^.

In plants, LMCOs play an important role in several developmental processes, notably lignin and flavonoid polymerization, as well as anthocyanin degradation responsible for pericarp browning during post-harvest storage of fruits. A recent study on litchi fruits identified a laccase as anthocyanin degrading enzyme responsible for pericarp browning during postharvest storage^4^.

The sequencing of the genomes/transcriptomes of different species, both herbaceous and woody, is providing an unprecedented depth of knowledge which can be used to identify the members of this class of oxidative enzymes in the plant kingdom. Sweet cherry is an important non-climacteric stone fruit (a drupe) valued worldwide; recent progress in sequencing its genome and transcriptome enables detailed insights into the molecular mechanisms which can be useful for breeding purposes, as well as for functional analyses^5,6^.

Sweet cherry is a highly perishable fruit, subject to postharvest bruising, pitting, stem browning, loss of red color^7^; hence, molecular analyses targeting specific classes of enzymes/genes intervening in postharvest stability are of great economic relevance. LMCOs have been shown to intervene in fruit postharvest stability, by affecting parameters such as anthocyanin degradation (fruit browning)^4,8^ and susceptibility to skin disorders (e.g. apple scald)^9^. Additionally, these oxidoreductases also partake in endocarp lignification in drupes and in the formation of the stone^10^. Therefore, they are of relevance for fundamental physiological studies.

We here identified at least 33 putative LMCOs in *P. avium* and analyzed them based on the phylogenetic relationship with thale cress. We have ranked them according to their relationship with the LMCOs of thale cress, litchi and strawberry. Additionally, by performing *in silico* structure modeling and docking studies, we propose potential substrates of a sweet cherry laccase showing sequence homology with a litchi LMCO involved in anthocyanin degradation^4^. Finally, we followed the expression of a subset in ancient Tuscan varieties sampled at commercial harvest (ca. 60 dpa, days post anthesis). These Italian regional varieties are non-commercial, but since they display higher content of polyphenols as compared to commercial fruits^11^, they constitute superior alternative sources of health-promoting compounds of interest for nutraceutics^11,12^. Therefore, the present work will be useful to investigate commercially-relevant features like color stability in such ancient fruits.

## Results

### Identification and bioinformatics of sweet cherry LMCOs

A total of 33 putative sweet cherry LMCOs were retrieved (FASTA sequences in Supplementary Information): the majority of the proteins were predicted to be extracellular due to the presence of a signal peptide (SP) (Table 1). Prediction of transmembrane (TM) regions with Phobius^13^ and TMHMM^14^ identified XP_021803024.1 as potentially membrane-bound. For the remaining sequences, Phobius did not detect any TM regions, while TMHMM predicted the presence of TM domains in XP_021833014.1, XP_021829870.1, XP_021833685.1, XP_017179926.1, XP_021833835.1, XP_007198990.1, XP_021819124.1, XP_007201720.2, XP_021827255.1, XP_021809222.1, XP_021825503.1, XP_021815052.1, XP_021819123.1, XP_021833693.1. However, since for these same sequences, the SP and/or localization predictions highlighted secretion (Table 1), only XP_021803024.1 was retained as potential membrane-bound protein. The accuracy of secretome prediction is indeed higher when combining different tools; for plants, the combination of SignalP/TMHMM/Phobius/TargetP provides specificity of 96.5% and sensitivity of 90.6%^15^.

**Table 1:** Details concerning the phylogenetic group, PROSITE MCO signature, signal peptide (SP) cleavage site and subcellular localization of the 33 putative sweet cherry LMCOs.

Accession number	Group	PROSITE (MCO signature, amino acid positions)	SP (cleavage site)	Localization
XP_021803024.1	6	540–560 PS00079 545–556 PS00080	27–28 But Phobius/TMHMM 1 TM domain	S
XP_021833014.1	2	516–536 PS00079 521–532 PS00080	25–26	S
XP_021829870.1	2	534–554 PS00079 539–550 PS00080	—	M L-S (CELLO)
XP_021833685.1	2	522–542 PS00079 527–538 PS00080	30–31	S
XP_017179926.1	2	540–560 PS00079 545–556 PS00080	31–32 (prediction with TargetP 1.1)	S
XP_021833835.1	2	522–542 PS00079 527–538 PS00080	30–31	S
XP_008246156.1	2	None (sequence is too short)	30–31	S
XP_007198990.1	2	521–541 PS00079 526–537 PS00080	29–30	S
XP_021834477.1	2	464–484 PS00079 469–480 PS00080	—	Any other location L-S (CELLO)
XP_021824778.1	3	541–561 PS00079 546–557 PS00080	32–33	S
XP_021819124.1	4	529–540 PS00080	26–27	S
XP_007201720.2	4	530–541 PS00080 1074–1085 PS00080 1069–1089 PS00079	27–28	S
XP_021820472.1	4	526–546 PS00079 531–542 PS00080	26–27	S
XP_021826148.1	4	533–553 PS00079 538–549 PS00080	24–25	S
XP_007227301.1	4	526–546 PS00079 531–542 PS00080	24–25	S
XP_021827255.1	4	529–540 PS00080	27–28	S
XP_021809222.1	1	541–561 PS00079 546–557 PS00080	31–32	S
XP_021809160.1	1	539–559 PS00079 544–555 PS00080	31–32	S
XP_021814279.1	1	545–565 PS00079 550–561 PS00080	31–32	S
XP_021825503.1	1	544–564 PS00079 549–560 PS00080	23–24	S
XP_021815052.1	4	532–552 PS00079 537–548 PS00080	19–20 (prediction with TargetP 1.1)	S
XP_021834606.1	5	522–542 PS00079 527–538 PS00080	22–23	S
XP_021809566.1	4	None (sequence is too short)	24–25	S
XP_021814580.1	2	517–537 PS00079 522–533 PS00080	22–23	S
XP_021816142.1	4	525–545 PS00079 530–541 PS00080	27–28	S
XP_021819117.1	4	522–542 PS00079 527–538 PS00080	26–27	S
XP_021819123.1	4	530–541 PS00080	27–28	S
XP_020426263.1	4	500–520 PS00079 505–516 PS00080	—	Any other location L-S (CELLO)
XP_007200191.1	4	526–546 PS00079 531–542 PS00080	24–25	S
XP_021829543.1	5	522–542 PS00079 527–538 PS00080	22–23	S
XP_021833316.1	4	521–541 PS00079 526–537 PS00080	26–27	S
XP_021820658.1	2	516–536 PS00079 521–532 PS00080	24–25	S
XP_021833693.1	2	543–563 PS00079 548–559 PS00080	30–31	S

The SP predictions were run with SignalP 4.1^17^, unless otherwise indicated in parenthesis. The localization predictions were run with TargetP 1.1^45,46^, or with CELLO^16^. S: secretory; M: mitochondrial; L: lysosomal.

Three LMCOs (XP_021829870.1, XP_021834477.1, XP_020426263.1) were predicted to be either mitochondrial or to be localized elsewhere; however, a parallel analysis with CELLO^16^ revealed the likelihood of either lysosomal or extracellular localization, although no SP was detected with SignalP 4.1^17^ (Table 1).

With the exception of two sequences, XP_008246156.1 and XP_021809566.1, which were shorter in length, all the other putative *P. avium* LMCOs were found to possess the MCO signature M2 (G-x-[FYW]-x-[LIVMFYW]-x-[CST]-x-{PR}-{K}-x(2)-{S}-x-{LFH}-G-[LM]-x(3)-[LIVMFYW], PROSITE PS00079) and/or M4 (H-C-H-x(3)-H-x(3)-[AG]-[LM], PROSITE PS00079)^18^ (Fig. S1, Table 1). Additionally, the alignment highlighted conservation of the L1 (H-W-H-G-x(9)-D-G-x(5)-Q-C-P-I) and L3 (H-P-x-H-L-H-G-H) regions containing the histidine residues involved in the binding of the T1, T2 and T3 Cu (Fig. S1).

**Figure 1:**
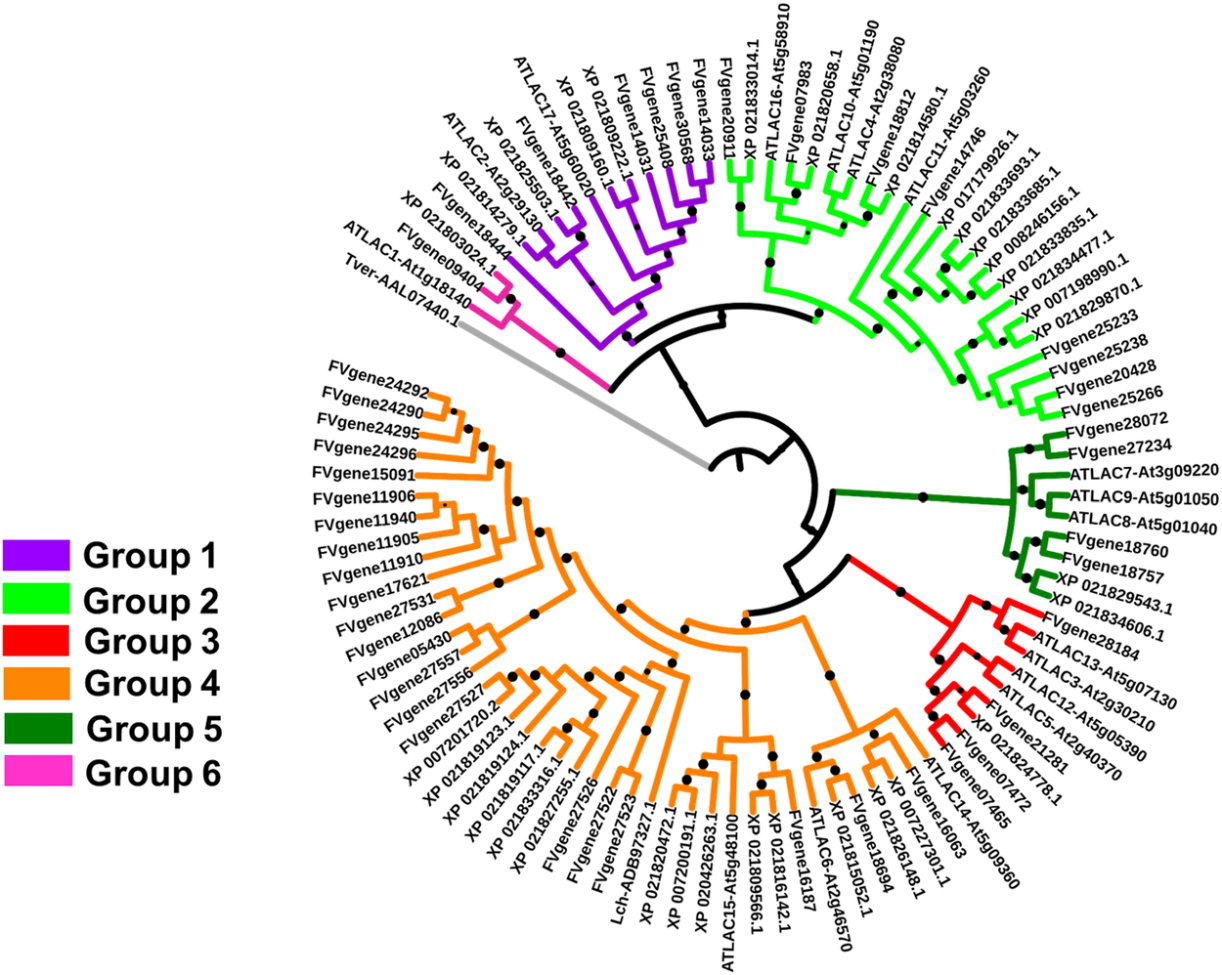
Maximum likelihood phylogenetic tree (bootstraps: 100) of the LMCO full length protein sequences of *A. thaliana* (AT; the gene codes are indicated), *F. vesca* (FV), *P. avium* and *L. chinensis* (Lch). The accession numbers of the LMCOs from wild strawberry, litchi and sweet cherry are indicated. The LMCOs belonging to the different groups reported in thale cress^1^ are indicated in different colors. *Trametes versicolor* laccase sequence (accession number AAL07440.1) was used as outgroup to root the tree. The FASTA full length protein sequences used to build the tree are provided in Supplementary Information. Boostrap values ranging from 0.7 to 1 are displayed as black circles; the bigger the circle, the higher the bootstrap value.

The phylogenetic analysis with LMCOs from *Fragaria vesca* and thale cress revealed the presence of the six groups (groups 1–6) previously reported in *Arabidopsis thaliana*^1^: 4 sweet cherry LMCOs belong to group 1, 11 to group 2, 1 to group 3, 14 to group 4, 2 to group 5 and 1 to group 6 (Table 1 and Fig. 1). We included in the phylogenetic analysis the laccase from *Litchi chinensis* previously reported to be responsible for anthocyanin degradation^4^; it clustered in group 4, where the sweet cherry LMCO giving the best correspondence after BLAST analysis, i.e. XP_021827255.1, is also found (Fig. 1).

### *In silico* structure modelling

For the 3D structure modelling, we based our analysis on the sweet cherry LMCO XP_021827255.1, the litchi LMCO ADB97327.1 involved in anthocyanin degradation^4^, the thale cress LAC15 At5g48100, the Japanese lacquer tree laccase (BAB63411.2) and 2 fungal enzymes, from an ascomycete (*Melanocarpus albomyces*; PDB, 3FU8) and a basidiomycete (*Trametes trogii*; PDB, 2HRG).

Fruit LMCOs showed highest identity to enzymes from plants and lowest identity to fungal ones. Both cherry and litchi showed intermediate identity to the plant ascorbate oxidase (Ao, Fig. S2a). It is interesting that the identity between fruit (litchi *vs* cherry) LMCOs is 61% and between ascomycete (*M. albomyces*, Ma) and basidiomycete (*T. trogii*, Tt) is only 31%, indicating that LMCOs do not show very high sequence similarity even between closely related members (Fig. S2a).

Multiple alignment between fruit, plant, fungal LMCOs and plant Ao is shown in Fig. S2b. The results show that all copper-interacting His residues and a Cys (red) are fully conserved across all groups. However, variation is found in the sequences among L1–L3 regions (unboxed green and cyan highlighted) and substrate-interacting loops (boxed). Further variation is found in the M2–M4 (purple highlight and underlined) region in the residues that may affect the redox potential of the enzyme (orange amino acids) and may interact with the substrate within the active-site (white amino acids on purple background). The active-site catalytic acidic residues in fungal laccases have been substituted by Asn residues in plant LMCOs (# blue amino acids; Fig. S2b).

**Figure 2:**
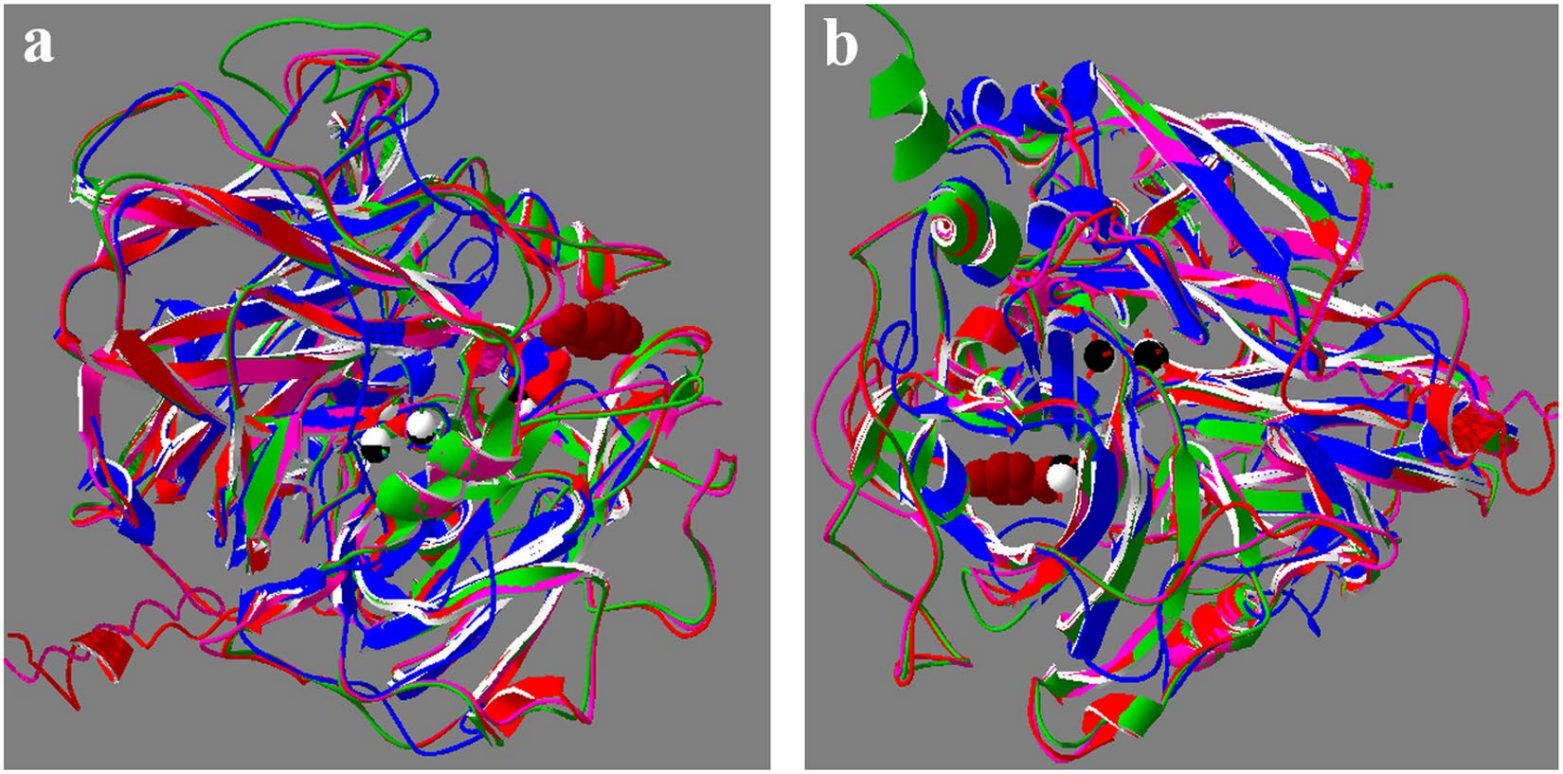
Models of fruit LMCOs superimposed on the X-ray structures of ascorbate oxidase (Ao) from *Cucurbita pepo* and laccase from *Trametes trogii* (Tt) and shown from two different perspectives (**a**,**b**). Red, cherry LMCO; pink, litchi LMCO; green, Ao; blue (Tt). Copper atoms from Ao (white) and Tt (black) are also superimposed. Substrate-binding pocket is depicted with brown space-filled substrate (2,6-dimethoxyphenol) and the copper atom nearest to it is T1. Blue/red space filled atoms showed His residue involved in abstracting electrons from the substrate and relying to the T1 Cu atom.

In the absence of a plant laccase X-ray structure, I-TASSER identified ascorbate oxidase (Ao) from *Cucurbita pepo* (Cp; PDB: 1ASP, 1AOZ) as the most suitable template to generate homology models of fruit LMCOs (Fig. 2). For both fruit models, the normalized Z-score for 1AOZ template was in the range 2–7 and >90% coverage. The C-score and TM-score were 0.55 and 0.79 ± 0.09 respectively for litchi and 0.34 and 0.76 ± 0.09 respectively for cherry LMCO. A C-score is typically in the range of [−5, 2], where a C-score of higher value denotes a model with a high confidence, whereas a TM-score > 0.5 indicates a model of correct topology. The quality of both fruit models based on the Ramachandran plots (results not shown) showed that for litchi and cherry models, 94.2 and 93.5% respectively of the residues were found in favored and allowed regions. The fruit models showed good superposition of the overall structure, copper atoms and electron abstracting His with each other, as well as with Ao (Fig. 2).

**Figure 3:**
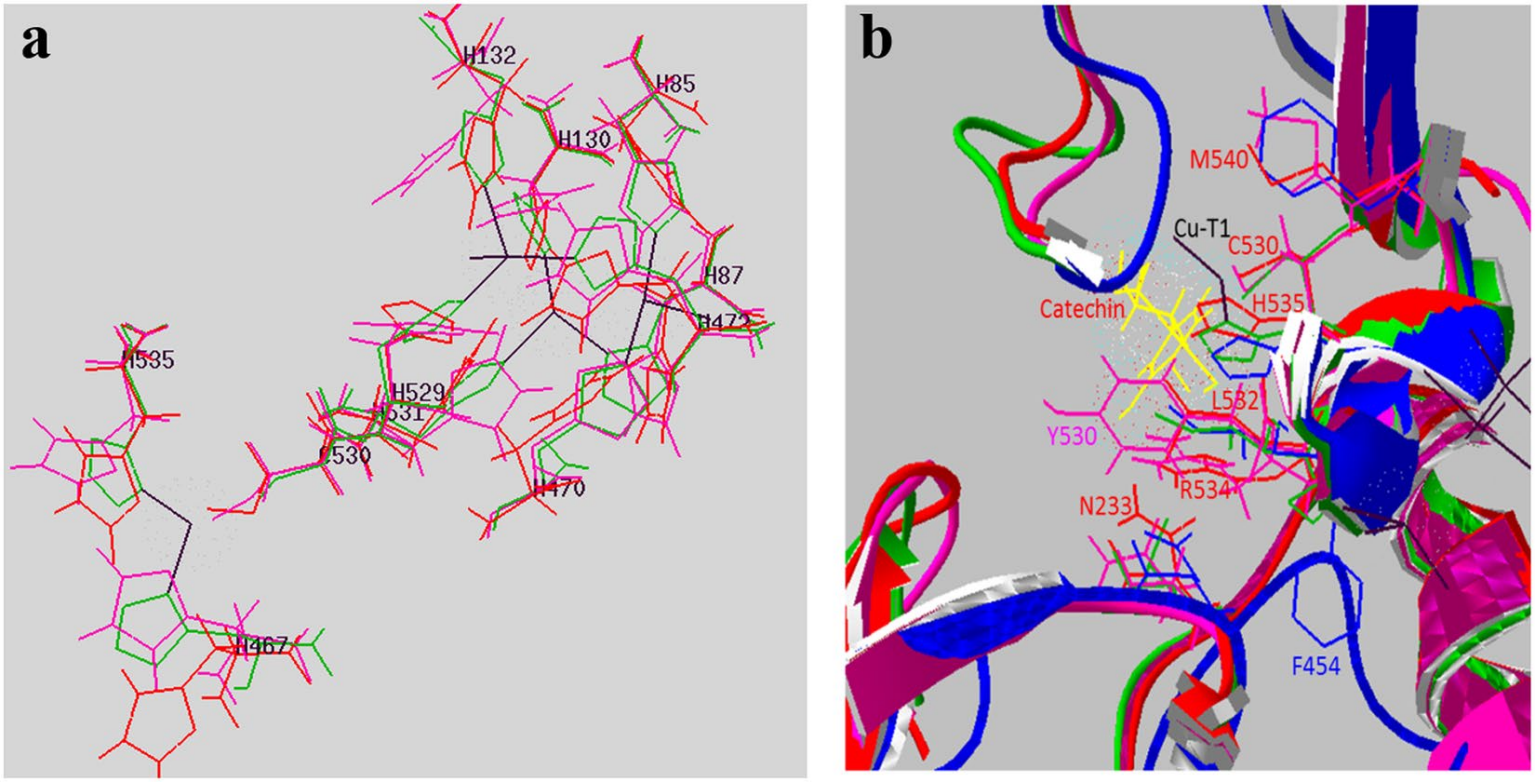
Fruit LMCOs modelled on the X-ray structure of ascorbate oxidase (Ao) along with the X-ray structure of fungal laccase from *Trametes trogii* (Tt) showing copper-interacting and critical catalytic residues. (**a**) Copper-interacting His and Cys residues. (**b**) Substrate-binding loops surrounding the active-site cleft and showing critical residues that interact with the substrate, involved in catalysis and redox potential. Red, cherry LMCO; pink, litchi LMCO; green, Ao; blue, Tt. Yellow, substrate (catechin); black lines, copper. Copper atom on the extreme left in both figures is T1. Residue numbering is based on cherry LMCO sequence and critical residues marked in Fig. S2b (multiple alignment) are depicted in their respective colors.

Figure 3a shows that copper-interacting His residues and a Cys residue in fruit LMCOs are superimposed nicely on those from Ao. The residues that interact with the catechin (yellow) within the active-site surrounded by substrate-binding loops are shown (Fig. 3b). The most critical residues are conserved, except for a residue affecting the redox –potential (cherry, L532), that is substituted by Tyr and Ile in litchi LMCO and Ao, respectively. Acidic residues (D/E) in fungal laccases are implicated in catalysis, but are replaced by Asn in plant LMCOs (N233 in cherry) and by Leu in Ao. Interestingly, a critical substrate-interacting residue that protrudes into the active-site is Arg (R534 in cherry) in all plant LMCOs, is substituted by aromatic (W, F) residues in fungi and Pro in Ao (Fig. 3b).

Various substrates ranging in size from 154 to 514 Da were docked in plant and fungal LMCOs and plant Ao (Table 2).

**Table 2:** The models of LMCOs from fruits compared to the X-ray structures of ascorbate oxidase from *Cucurbita pepo* and LMCO from fungi.

Organisms Substrates	Ao (*Cucurbita pepo*), LMCO (Cherry)	LMCO (Litchi), Laccase (lacquer tree)	Fungal LMCO: *M. albomyces, T. trogii*
2,6-dimethoxyphenol MW, 154.17	Affinity: -4.3, -4.0	Affinity: -4.7, -4.6	Affinity: -4.3, -4.2
			
			
			
			
			
			

Affinity, free-energy of enzyme-substrate binding (more negative value depicts better binding); Ao, Ascorbate oxidase (green); lacquer tree, *Toxicodendron vernicifluum*, (*yellow*); cherry LMCO (red); litchi LMCO (pink); *M*. *albomyces, Melanocarpus albomyces* (ascomycete, turquoise); *T*. *trogii*, *Trametes trogii* (basidiomycete, blue); respective colored space-filled amino acids, surface-exposed His residue involved in substrate binding that acts as a primary electron acceptor involved in shuttling electrons from the substrates (shown in various colors) to T2 and T3 copper (white spheres) via T1 Cu (black sphere), conserved Cys and His residues (black).

The results show that both fruit LMCOs have the largest affinity with catechin and quercetin as substrates, although litchi shows higher affinity than cherry LMCO for both of these substrates. Whereas Ao and cherry LMCO show lowest affinity with the smallest substrate (2, 6-dimethoxyphenol), litchi LMCO showed lowest affinity with the largest substrate (ABTS). This is probably because litchi LMCO is unable to fully accommodate bent ABTS into the active-site (Table 2, in pink), whereas the lacquer laccase shows high affinity (ΔG, −7), as the bent ABTS is fully accommodated in the active-site (Table 2, in yellow). Generally, cherry LMCO shows lowest affinity, whereas laccases from lacquer tree and ascomycete (*M. albomyces*) show the highest affinity for all substrates tested. The variation in binding affinity may be due to the presence of non-conserved residues (such as small *vs* large hydrophobic residues) in and around the substrate-binding loops that may change the size of the active-site pocket, as well as interactions with the substrates (Figs 2 and 3b; Table 2).

The surface morphology analysis for selected LMCOs and Ao in the presence of all substrates is shown in Table 3. The analysis shows the variation in the conformations (extended *vs* bent) of the substrates, as well as variation in the contours (shallow *vs* deep) of the active-site of enzymes.

**Table 3:** Poses of various substrates within the binding-site of LMCO from litchi and sweet cherry, laccase from lacquer tree and ascorbate oxidase (Ao) from plants.

Substrates	Ao (*Cucurbita pepo*)	LMCO (Cherry)	LMCO (Litchi)	Laccase (lacquer tree)
2,6-dimethoxyphenol				
Coniferylalcohol				
Catechin, Cianidanol				
Quercetin				
Secoisolariciresinol				
ABTS				

All laccase and LMCO models were generated using the X-ray structure of Ao from *Cucurbita pepo*. Refer to Table 1 for the properties of all substrates. Red surface, polar; white surface, non-polar.

### Gene expression analysis in the ancient and commercial varieties

To select the LMCO genes for RT-qPCR analysis, we decided to focus our attention on the transcriptomic study previously published on sweet cherry fruit development^5,6^ and designed primers on the contigs encoding putative laccases present in the dataset. We reasoned that those genes would represent the LMCO members expressed (and hence detectable via RT-qPCR) in the fruit tissues of our sweet cherry varieties. Some of the putative LMCOs were however expressed at very low levels in the ancient sweet cherry fruits and were therefore discarded from the subsequent analyses. Indeed, the corresponding primer pairs did not amplify with the range of efficiency accepted for robust expression analysis. A total of nine primer pairs passed the quality control for the amplification efficiencies. Of these nine targets, the BLAST analyses revealed that three belonged to the same transcripts, according to the latest sweet cherry genome assembly^5,6^. Therefore, in the end, six LMCO genes were targeted for gene expression studies, i.e. the genes encoding XP_021833316.1, XP_021824778.1, XP_021809222.1, XP_021834477.1, XP_021814580.1 and XP_021820658.1.

**Figure 4:**
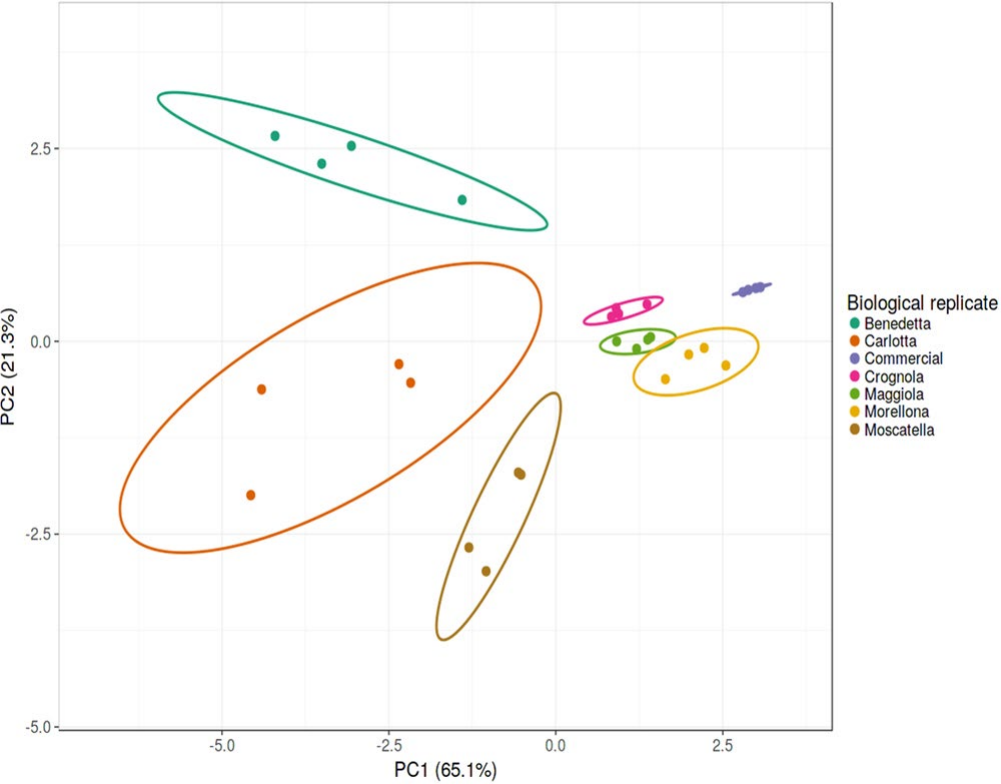
Principal Component Analysis (PCA) of the gene expression data. Circles indicate the 4 biological replicates for each local variety (indicated using different colors, according to the legend on the right-hand side).

The Principal Component Analysis (PCA) of the gene expression data shows co-clustering of the 4 independent biological replicates for each variety studied, as well as a good separation of the varieties (Fig. 4). The first 2 components of the PCA explain 86.4% of the total variance. More specifically, PC1 represents 65.1% of the total variance, while PC2 21%. It is noteworthy that the commercial variety ‘Durone’ forms a distinct group that is well separated from the ancient varieties here investigated.

**Figure 5:**
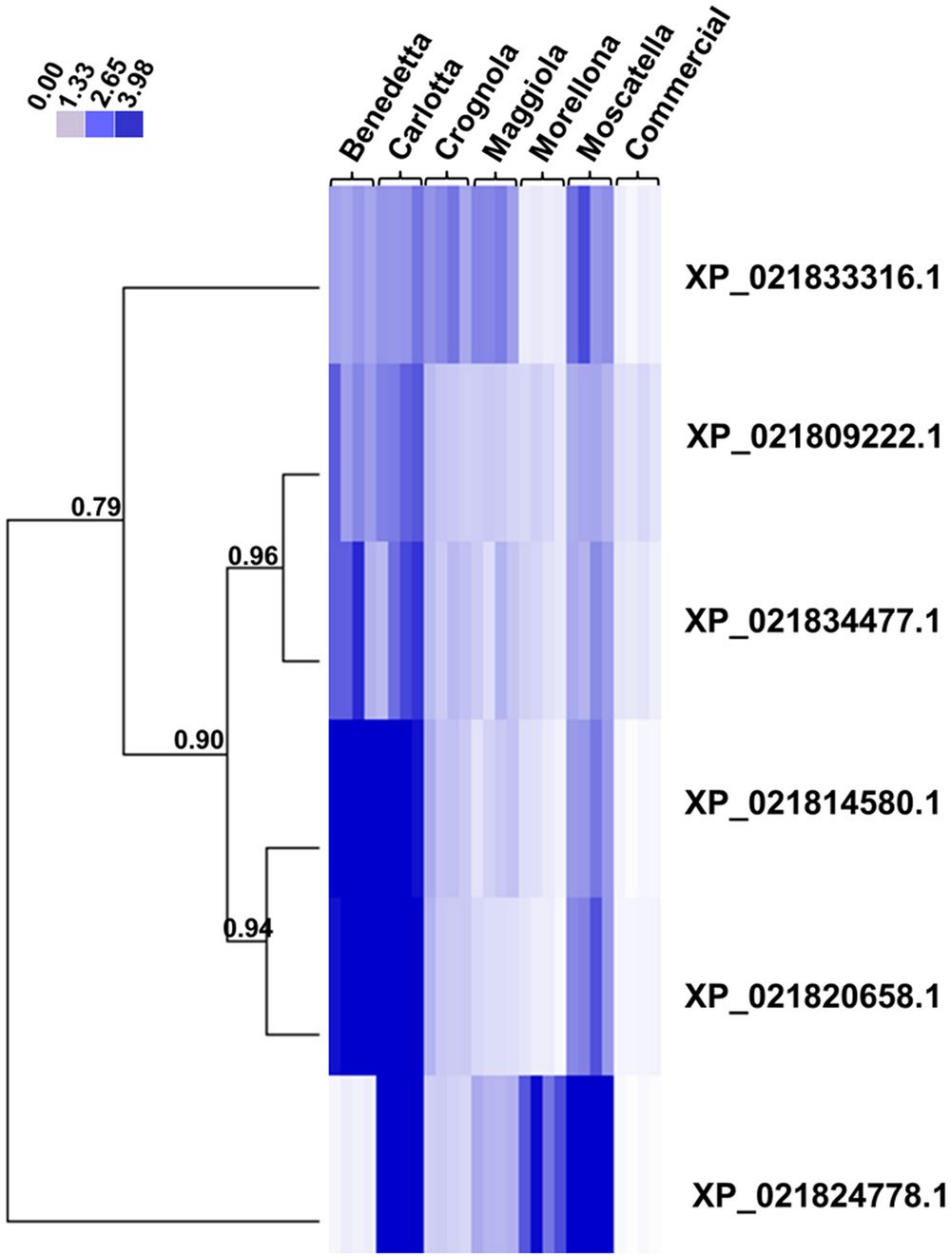
Heat map hierarchical clustering of the six LMCO gene expression data. The accession numbers indicate the proteins encoded by the corresponding genes. Numbers indicate the Pearson correlation coefficients. For each variety, the four biological replicates are displayed. The color bar indicates the expression intensities.

The targeted gene expression analysis shows that the six LMCOs analyzed have statistically significant higher expression in almost all the ancient varieties with respect to the commercial one investigated in the study (Figs 5 and S3).

In particular, four major expression patterns can be identified via the hierarchical clustering of the heat maps (absolute Pearson correlation, average linkage): the genes corresponding to XP_021833316.1 and XP_021824778.1 cluster in two branches, while those coding for XP_021809222.1 and XP_021834477.1, together with XP_021814580.1 and XP_021820658.1 form a third and a fourth expression pattern (Fig. 5). In particular, the genes corresponding to XP_021809222.1, XP_021834477.1, XP_021814580.1 and XP_021820658.1 are characterized by higher expressions in the varieties ‘Benedetta’, ‘Carlotta’ and ‘Moscatella’, with however a more marked pattern for the cluster formed by XP_021814580.1 and XP_021820658.1 (Fig. 5). The only LMCO gene showing much lower expression in ‘Benedetta’ with respect to the other ancient varieties is the one corresponding to XP_021824778.1. Notably, this gene is instead expressed at higher levels in ‘Moscatella’, a variety displaying lower expressions of the other LMCOs investigated (Figs 5 and S3).

## Discussion

Sweet cherry is an economically important tree whose fruits are appreciated for their taste, high content in polyphenols and hence their nutraceutical value^19,20^. The availability of the genome and transcriptome of *P. avium*^5,6^ is a great asset for breeding strategies, as well as for more basic functional studies on genes controlling important fruit parameters, like color, content of bioactives, size, post-harvest stability.

We have here focused our attention on a class of enzymes, the LMCOs, that is still poorly explored in plants, despite their enormous physiological importance. It is for example known that LMCOs control crucial plant physiological processes like lignification^21^, response to exogenous stresses^22^ and post-harvest stability of fruits^4,8,9^.

Our study, based on a comparative analysis with thale cress and *F. vesca*, another member of the family *Rosaceae*, has identified (at least) 33 LMCOs in sweet cherry, the majority of which corresponds to secreted enzymes possessing the reported motifs L1, M2, L3 and M4 of laccases and MCOs^18^ (Table 1).

The 33 *P. avium* LMCOs cluster in the six phylogenetic groups previously identified in *A. thaliana*^1^ (Fig. 1). Attempts to retrace the physiological role of LMCOs on the basis of characteristics such as pI proved not to be appropriate^1^. In the absence of functional studies, it was proposed that any prediction of function should be based on sequence analysis and phylogenetic clustering^1^. To this end, we included both thale cress and strawberry LMCOs in our analysis, since, for both species, electronic fluorescent pictograms (eFP) are available for different tissues and conditions^23,24^. It is for example possible to notice that the cluster of sweet cherry LMCOs belonging to group 4 and comprising those proteins clustering in a sister group with respect to ATLAC15, i.e. XP_021820472.1, XP_007200191.1 and XP_020426263.1, may be involved in the metabolism of proanthocyanidins (PAs), as well as lignification of the seeds and root elongation, in a manner analogous to what shown in *A. thaliana*^25,26^. It will therefore be interesting to study the expression of the corresponding *P. avium* LMCOs in the seed.

As previously mentioned, XP_021827255.1 is the best *P. avium* BLAST match of the litchi LMCO involved in pericarp browning: it is interesting to note that the strawberry LMCO encoded by gene27526 (giving the best match after BLAST search) is expressed at the highest levels in the fruit cortex and pith at stage 1 and 2 (http://mb3.towson.edu/efp/cgi-bin/efpWeb.cgi). Hence, BLAST analysis, coupled to phylogeny and eFP database search can provide an indication of the tissues where the gene is expressed at the highest levels and consequently, of the potential role in sweet cherry. More specific studies relying on gene expression at different developmental stages and post-harvest conditions will provide more solid indications of the LMCO role in cherry fruits.

The sweet cherry LMCOs XP_021814580.1 and XP_021809160.1, XP_021809222.1 clustering with ATLAC4 and ATLAC17 (Fig. 1), may be involved in pit lignification, since the corresponding thale cress genes are known to regulate lignification^27^. The corresponding strawberry LMCO encoded by gene18812 is expressed at higher levels in the carpel wall in the strawberry eFP database, a finding corroborating the potential involvement in lignification of the *P. avium* related LMCOs.

In the absence of a plant laccase X-ray structure, both fruit LMCOs (XP_021827255.1, ADB97327.1) were modelled on the Ao template^28,29^. The functional role of Ao (EC 1.10.3.3) has been a mystery due to its requirement for ascorbate as a sole substrate with obvious disadvantage in lowering plant resistance against stress as a result of ascorbate depletion^30^. Our results indicate that sequence alignment (Fig. S2b), overall protein fold (Fig. 2), copper-binding residues and active-site morphology (Fig. 3, Tables 2 and 3) of Ao are quite similar to LMCOs from cherry and litchi. Additionally, the binding affinities of numerous substrates including fruit-specific catechin to Ao are either comparable or better than cherry and litchi LMCOs (Table 2), suggesting that Ao likely acts on other substrates as well. Only few substrates other than ascorbate have been tested with Ao. It has been experimentally found that Ao can act on other substrates (chlorohydroquinone derivatives) albeit with lower (6–8%) activity but higher affinity for smaller chlorohydroquinone and lower affinity for slightly larger 2,6-dichlorohydroquinone compared to that with ascorbate^31,32^. In the future, it will be interesting to experimentally verify the substrate specificities (V_max_ and K_m_) of fruit LMCOs and compare them to Ao (Table 2).

Substrate-binding loop I has a catalytic residue (#Figs S2b and 3b). Hydrophilic Glu/Asp in fungal laccases are believed to be involved in reaction mechanism by making H-bond with the substrate and modulate substrate specificity and pH profile^33^. It is interesting that in cherry LMCOs it can be hydrophilic Asp, Asn or Gln (Fig. S1), whereas in Ao it is hydrophobic Leu (#Figs S2b and 3b). This hydrophilic *vs* hydrophobic substitution is intriguing in view of thevresults showing good affinity of substrates with Ao (Table 2)^31,32^, but low catalytic activities^31,32^. The implication of hydrophobic Leu in the low activity of Ao against common laccase substrates can be experimentally verified by replacing it with hydrophilic amino acids found in fruit laccases. Substrate-binding loop IV has a surface-exposed His (#Figs S2b, 2 and 3) involved in substrate interaction via H-bond and likely acts by accepting an electron from the substrate and relaying it to T1 Cu, which in turn passes it to T2/T3 copper center via Cys residue^27^ (Fig. 3a). While Arg in plants (Fig. S2b; R534 in cherry; Fig. 3b) and Pro in Ao protrude into the active site and likely stabilize deprotonated hydroxyl groups and hydrophobic rings of substrates respectively, Trp507/Phe454 in fungal LMCOs are shown to project away from the active-site (Figs S2b and 3b).

The redox potential of LMCOs determines which substrates can be oxidized^34–37^. Two axial residues found in substrate-binding loop IV (Fig. S2b, 1^st^ orange amino acids within the blue box; Fig. 3b) have been implicated in controlling the redox potential. The first axial residue is Ile in fungi^33^, in plants, it may be I, L, Y, F, in cherry it may be I, L, F (Fig. S1). In litchi and lacquer tree LMCOs it is an aromatic amino acid such as Tyr and Phe (Figs S2b and 3b). The higher hydrophobicity at this position can raise the redox potential of the enzyme^36^.

The second axial residue is also known to influence the redox-potential of laccase (Fig. S2b, 2^nd^ orange amino acids within the grey box, Fig. 3b). In plants, including cherry LMCOs, it is Leu or Met (Fig. S1). In fungal laccases it is Leu or Phe. Met causes a decreased redox potential. Phe has highest redox potential, followed closely by Leu and Met^34,35^. In addition, the presence of hydrophobic residues near the T1 Cu and the distance between the T1 Cu and nitrogen atom of electron abstracting His (S2, red#) can also raise the redox potential^30^, beside other factors^31^. A large distance renders T1 Cu more electron deficient thus increasing its redox potential^31^. The distances between T1 Cu and His nitrogen were determined to be 1.93, 2.03 and 2.1 Å in Ma, Tt and Ao X-ray structures respectively. Uncertainties in the homology models preclude the determination of T1 Cu-His distances in fruit laccases.

The gene expression analysis highlighted an overall higher expression of the six LMCOs in the local varieties from Tuscany, as compared to the commercial one (Figs 5 and S3). This finding is interesting if one considers that the polyphenol contents in the local varieties are higher than those found in the commercial one^11^. Particularly interesting in this respect is the expression profile in the variety ‘Benedetta’, which was previously found to possess high contents of catechins with respect to the other ancient varieties here investigated^11^.

Catechins are the monomeric units of proanthocyanidins (PAs, also referred to as condensed tannins) and this class of oligomeric/polymeric flavonoids shows great variety depending on stereochemistry and hydroxylation pattern^38^. Cherries are rich in procyanidin B2 (a B-type PA)^38^: the Phenol Explorer database (http://phenol-explorer.eu/contents/food/46) specifies that they are reported to contain 2.10 mg/100 g FW^39^. It would therefore be interesting to measure the content of PAs in the ancient Tuscan varieties to confirm whether ‘Benedetta’ has higher contents reflecting the increased quantities of catechins previously quantified^11^.

Likewise, it will be interesting to explore whether the laccases displaying the highest expression in ‘Benedetta’ (i.e. the genes coding for XP_021814580.1 and XP_021820658.1) are involved in the oxidative polymerization of catechins in sweet cherry. In this respect it should be noted that polyphenol oxidases (PPOs), also proposed to be responsible for the oxidative polymerization of flavonols, do not usually give PAs with structures found in nature^40^. Hence, LMCOs may partake *in vivo* in the polymerization of PAs, a process that is still not fully characterized nor elucidated^41^. In support of a role of LMCOs in PA polymerization is the *TRANSPARENT TESTA 10* (*TT10*) mutation in thale cress (*TT10* encodes a LMCO-type flavonoid oxidase): the *tt10* mutant shows a pale-brown seed coat phenotype (due to alterations in seed coat pigmentation), as well as accumulation of soluble PAs and more epicatechin monomers^25^.

LMCOs from plants are still undercharacterized: no 3D structures are yet resolved and only limited knowledge of their functions, even in model systems such as thale cress, is available^42^. The gene redundancy and substrate promiscuity make their study in plants difficult and complex. However, an increased knowledge of LMCOs function in higher plants would give impetus to biotechnology: being mostly secreted, cultivation in bioreactors of bacterial/yeast cells expressing the target plant LMCOs could provide a valuable tool for the production of novel oxidative enzymes that could be used as catalysts in green chemistry. Alternatively, undifferentiated plant cells could be grown in bioreactors and their LMCOs purified from the culture medium. Being enzymes that mediate the response to exogenous stresses, elicitation can be envisaged to boost their production in plant cell cultures. However, protein yield is a limiting factor making the use of engineered bacterial/yeast cells more favorable from a practical point of view.

## Conclusions

We have here characterized the sweet cherry LMCOs using *in silico* and gene expression studies. The phylogenetic analysis has provided a classification in the 6 major groups previously identified in thale cress. The modeling and substrate docking analyses of the sweet cherry LMCO displaying the highest sequence similarity with the litchi enzyme involved in anthocyanin degradation showed affinity for catechin and quercetin. The gene expression analysis highlighted a higher expression of a set of LMCO genes in the ancient non-commercial varieties from Tuscany. Ours is the only work addressing the study of LMCOs from ancient sweet cherry varieties of Tuscany. The rationale behind this study is the willingness to valorize local varieties as alternative (and better) sources of bioactive molecules, as compared to commercial ones. Since LMCOs impact fruit parameters during post-harvest storage, we deemed it interesting to study this class of oxidoreductases in sweet cherry and to measure their expression in ancient Tuscan varieties. The data presented have a dual significance. On one hand they will promote conservation and further studies of the ancient fruit trees representing the agrobiodiversity heritage of Tuscany. Additionally, the data here presented will pave the way to further research addressing the molecular analysis of LMCOs potentially intervening in important physiological processes in sweet cherry, e.g. flavonoid polymerization, stone formation, or impacting industrially-relevant aspects, like anthocyanin degradation during fruit post-harvest storage.

## Materials and Methods

### Sample collection

Sampling was carried out in 2017 on 18-year-old cherry trees (ancient local varieties of *P. avium* on P-HL-B rootstocks) grown under standard horticultural conditions at the experimental field of the CNR in Ivalsa Follonica (GR, Italy). The variety Benedetta was sampled in 2016, since no fruits were obtained in 2017. The experimental field is located at the following coordinates: 42°55′59″N, 10°45′57″E. For each variety, the corresponding trees were present in different numbers, depending on how many could be recovered across Tuscany. The total number of trees for each variety is: ‘Benedetta’ (2), ‘Maggiola’ (8), ‘Morellona’ (5), ‘Crognola’ (3), ‘Carlotta’ (4), ‘Moscatella’ (5). A total of 20 cherry fruits were taken from each tree to have enough biological replicates (4 in this study), each consisting of a pool of at least 5 fruits.

Samples were collected on May the 16^th^ 2016 and May 19^th^ 2017 from trees grown under field conditions and exposed to natural variations of temperature and solar radiation. The sample collection took place by harvesting fruits located at different places on the tree, in order to minimize the bias due to variations in solar exposition. Harvesting took place at an average temperature of 17–20 °C (min 12 °C-max 22 °C in 2016, min 13 °C-max 26 °C in 2017) and at an average of 71–74% of humidity in 2016 and 2017, respectively. All the fruit samples used for the gene expression analyses were harvested in the morning between 9:00–10.00 am and were positioned at around 1.70–1.90 m from the soil. Sweet cherry fruits were picked at the stage of commercial standard, i.e. ca. 60 dpa. Each fruit was harvested from the tree by detaching it with the stem, which was subsequently rapidly removed. After removal of the stem, the fruits were immediately plunged in liquid nitrogen, brought to the laboratory and stored at −80 °C in Ziplock bags until RNA extraction. The commercial variety ‘Durone’ was purchased at a local grocery shop in Siena.

**Figure 6:**
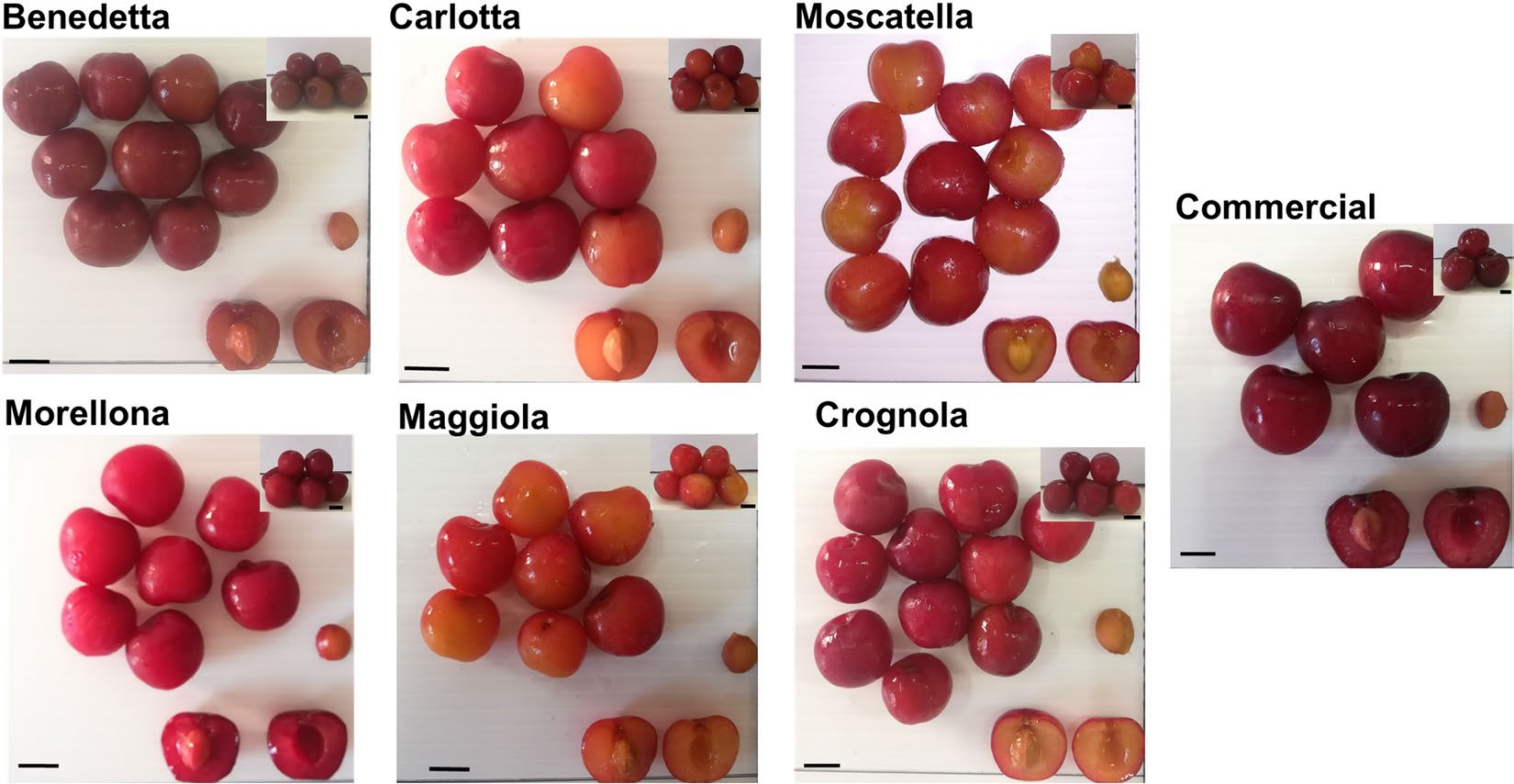
Images showing the different varieties studied. Insets show lateral views of the cherries. Scale bars: 1 cm.

The phenotypic characteristics of each variety are shown in Fig. 6. All the varieties here studied are preserved in the Regional Bank of Germplasm of Tuscany (http://germoplasma.regione.toscana.it/index.php?option=com_content&view=article&id=4&Itemid=109)^12^.

### Bioinformatic analyses

Sweet cherry LMCOs were obtained by blasting the thale cress protein sequences in NCBI, as well as by querying the Genome Database for Rosaceae (GDR; available at https://www.rosaceae.org/) and DBcherry database (available at http://cherry.kazusa.or.jp)^6^. The phylogenetic analysis was carried out by using the full length LMCOs of thale cress^1^, strawberry (downloaded from the Phytozome portal, at https://phytozome.jgi.doe.gov/pz/portal.html), the litchi enzyme reported to be involved in pericarp browning^4^ and a laccase from the polypore mushroom *Trametes versicolor* to root the tree. The FASTA sequences are given in Supplementary Information. The pair-wise multiple alignment of LMCOs for identifying conserved residues and motifs was determined by using CLUSTAL-Ω (http://www.ebi.ac.uk/Tools/msa/clustalo/)^43^ and the alignment was then used to build the maximum likelihood phylogenetic tree using the online program PhyML (bootstraps: 100), available at http://www.phylogeny.fr/one_task.cgi?task_type=phyml ^44^. The tree was visualized with iTOL (available at https://itol.embl.de/).

The presence of a signal peptide (SP, and the cleavage site) was predicted with the SignalP 4.1 server^17^ (available at http://www.cbs.dtu.dk/services/SignalP/); subcellular localization predictions and SP additional analysis were performed with the TargetP 1.1 server^45,46^ (available at http://www.cbs.dtu.dk/services/TargetP/). The online software CELLO was also used to predict subcellular localization^16^ (available at http://cello.life.nctu.edu.tw/). The occurrence of the MCO signature was verified with the ScanPROSITE tool^47^ (available at https://prosite.expasy.org/scanprosite/). Prediction of transmembrane regions war performed with TMHMM Server v. 2.0^14^ (available at http://www.cbs.dtu.dk/services/TMHMM/) and Phobius^13^ (accessible at http://phobius.sbc.su.se/index.html).

The Principal Component Analysis (PCA) was performed with ClustVis^48^ (available at https://biit.cs.ut.ee/clustvis/).

The heat map hierarchical clustering (absolute Pearson correlation, average linkage) was carried out with Cluster 3.0^49^ and visualized with Java TreeView^50^.

Data from the electronic fluorescent pictogram (eFP) browser of thale cress and strawberry^23,24^ were obtained by searching the target genes in http://bar.utoronto.ca/efp/cgi-bin/efpWeb.cgi and http://mb3.towson.edu/efp/cgi-bin/efpWeb.cgi.

Copper-interacting residues were identified in the X-ray structures of LMCOs and ascorbate oxidase using PDBsum available at http://www.ebi.ac.uk/thornton-srv/databases/cgi-bin/pdbsum/GetPage.pl?pdbcode=index.html^51^. Substrate-binding loops and copper-interacting residues were identified as described previously^37^. The 3D homology models were generated with the I-TASSER Suite (http://zhanglab.ccmb.med.umich.edu/I-TASSER/)^52^ utilizing LOMETS, SPICKER, and TM-align. The models were then refined using REMO by optimizing the backbone hydrogen-bonding networks and FG-MD by removing the steric clashes and improving the torsion angles. The quality of the models was checked by RAMPAGE^53^.

Molecular docking of substrates of various sizes with LMCOs was carried out with the online Mcule tool using reference His 514N4080 atom of ascorbate oxidase structure and equivalent atoms in all other structures^54^ based on AutoDock Vina^55^ (http://doc.mcule.com/doku.php?id=1clickdocking). The structures of substrates were imported into Mcule from PubChem using SMILES codes (https://pubchem.ncbi.nlm.nih.gov/). The final structures along with docked substrates showing copper- and substrate-interacting residues were visualized by superimposing fruit models on the fungal (*Trametes trogii*, PDB 2HRG) laccase and ascorbate oxidase from plant (*Cucurbita pepo*, PDB 1ASP) with DeepView Swiss-PdbViewer v4.1 (http://www.expasy.org/spdbv/)^56^.

### RNA extraction, cDNA synthesis, primer design and real-time PCR

Sweet cherry fruits were taken from the −80, kept frozen in liquid nitrogen and quickly cut into pieces (comprising the exocarp and ca. 5 mm of the mesocarp tissue) using a sterile liquid nitrogen-cooled scalpel. The tissue pieces were collected in a pre-sterilized frozen mortar filled with liquid nitrogen and immediately ground to a fine powder using a pestle. This procedure was necessary to ensure extra care, as previous tests showed that the RNA of sweet cherry fruits is extremely sensitive to degradation caused by even minimal tissue thawing. RNA was extracted using the modified CTAB procedure previously reported by us for textile hemp^57,58^. In case of low A260/230 nm ratios, a further precipitation/wash step with ammonium acetate/ethanol was performed^57^. The RNA integrity values (RINs) were determined using a 2100 Bioanalyzer (Agilent, Santa Clara, CA, USA); for all the samples the RINs were >7.5. The extracted RNAs were converted to cDNA with the ProtoScript II reverse transcriptase (New England Biolabs, Leiden, The Netherlands) and random primers, according to the manufacturer’s instructions. The cDNA was diluted to 2 ng/μL and used for the RT-qPCR analysis in 384-well plates which were prepared with an automated liquid handling robot (epMotion 5073, Eppendorf, Hamburg, Germany). Primers were designed with the online tool Primer3Plus (http://www.bioinformatics.nl/cgi-bin/primer3plus/primer3plus.cgi) and subsequently cross-checked using the OligoAnalyzer 3.1 tool from Integrated DNA technologies (http://eu.idtdna.com/calc/analyzer). The FASTA nucleotide sequences of the sweet cherry reference genes and LMCOs are given in Supplementary Information. The RT-qPCR reactions were set up and run as described previously^55^. A melt curve analysis was performed at the end of the amplification cycles, in order to assess the specificity of the primers. The primer efficiencies were determined using a 5-fold dilution series of 6 points (10–2–0.4–0.08–0.016–0.0032 ng/μL) and are reported in Table 4.

**Table 4:** List of primer names, sequences, amplicon size and amplification efficiencies used in this study for RT-qPCR.

Name	Sequence (5′ → 3′)	Amplicon size	Amplification efficiency
PavAP4 Fwd	CCATGATCTCGCAGTTCTTC	137	1.927
PavAP4 Rev	CTCTTGCCCATCTTCTTTCC		
PavTip41 Fwd	GAAATGGTGTTTGGGGACAG	122	1.890
PavTip41 Rev	ACTTCAACTGGTGGCAAAGC		
PavPP2A Fwd	CTTTCCCCATCTTTGGACAC	118	2.048
PavPP2A Rev	CCACAAGAGATCGCACATTG		
PavAct7 Fwd	CCATGTATGTTGCCATCCAG	149	1.973
PavAct7 Rev	AAGGTCCAGACGAAGAATGG		
PavPolyUbq Fwd	CCCTTGCGGATTACAACATC	87	2.063
PavPolyUbq Rev	GGGTCTTCACGAAAATCTGC		
PavSerThr Fwd	AACTCAATCCGCAGGCTATC	149	1.994
PavSerThr Rev	TATGGAATGAAGACCCCCAA		
XP_021824778.1 Fwd	TCGTCGTCAAAGTCACCAAC	81	1.940
XP_021824778.1 Rev	CCCATCCAGTTCTCATTTGC		
XP_021820658.1 Fwd	ACCCCAGAAAGAAGTGGTTG	91	2.013
XP_021820658.1 Rev	GGCTGAGCCAGATTTCAAAG		
XP_021814580.1 Fwd	TTCATGGACTCTCCCATTGC	88	2.093
XP_021814580.1 Rev	AGAGTTGTGGATGTGGTTGC		
XP_021834477.1 Fwd	GGAATCAACCAATGCACGAC	87	1.930
XP_021834477.1 Rev	TCCAATTTGGGGCATCAC		
XP_021809222.1 Fwd	CTTCTCCGCCTAATCAATGC	92	2.060
XP_021809222.1 Rev	TAAACGGCATCGGCTTCTAC		
XP_021833316.1 Fwd	AACTGTGAACGGGACTTTGC	83	1.911
XP_021833316.1 Rev	AGCCTTGGTTGTGGACATTC		

The expression of the LMCOs was calculated with qBase^PLUS^ (version 2.5, Biogazelle, Ghent, Belgium) by using the reference genes indicated by the geNorm^PLUS^ analysis (6 reference genes were tested for stability and *PavAP4* and *PavTIP41* were identified as sufficient for data normalization among *PavPP2A*, *PavPolyUbq*, *PavSerThr*, *PavAct7*). A one-way ANOVA with a Tukey’s post-hoc test was performed on log2 transformed NRQs (Normalized Relative Quantities) by using IBM SPSS Statistics v19.

## Supplementary information

Suppl. Info
