# Supplementary material for: Identification of the laccase-like multicopper oxidase gene family of sweet cherry (Prunus avium L.) and expression analysis in six ancient Tuscan varieties
Source: Sci Rep. 2019 Mar 5;9:3557. doi: 10.1038/s41598-019-39151-z (PMC6401077; doi:10.1038/s41598-019-39151-z)
Supplement: Supplementary file 1 — Suppl. Info [file 41598_2019_39151_MOESM1_ESM.pdf]

Identification of the laccase-like multicopper oxidase gene family of sweet cherry (*Prunus avium* L.) and expression analysis in six ancient Tuscan varieties

Roberto Berni[1,2], Emilie Piasecki[3], Sylvain Legay[3], Jean-Francois Hausman[3], Khawar Sohail Siddiqui[4,*], Giampiero Cai[1,*] and Gea Guerriero[3,*]

[1]Department of Life Sciences, University of Siena, via P.A. Mattioli 4, 53100 Siena, Italy.

[2]Trees and timber institute-National research council of Italy (CNR-IVALSA), via Aurelia 49, 58022 Follonica (GR), Italy.

[3]Research and Innovation Department, Luxembourg Institute of Science and Technology, 5 avenue des Hauts-Fourneaux, L-4362 Esch/Alzette, Luxembourg.

[4]Life Sciences Department, King Fahd University of Petroleum and Minerals (KFUPM), 31261 Dhahran, Saudi Arabia.

[*]Authors to whom correspondence should be addressed: sohailsiddiqui1995@yahoo.com; giampiero.cai@unisi.it; gea.guerriero@list.lu

FASTA sequences of the genes used for RT-qPCR

```
>XM_021971657.1 TIP41-like protein
AATAAATAATTGTATAAACCGTTAACACAAACCCGGAAAAAGTTTGAGGCAGTATCAGCTCCGAAGTCCG
AGTCTCGTTTAAGAGACCCGCCCAAGTCGTCGACACTCACAGTAGTATAGAGAAGCCAGAACAAAAACGC
GAAGCGACGGAAGAAGAAAGAAGAGAAGCAAGGGAAAACCAGGAGAATCGGTGAGCAGAAAAATGGAAGT
GGAGGTTGACGAGAAGGAGCTGAAAGCCGCCGGAGCTGAGCCGTTGACCGACGGACGCCATGGCCTCCGT
ATCCATGGTTGGGAAATCGAGTCTCGCAAGCGCTCCATTCTCAAGTCCTCCACCCTCGAACTGTGGGAGA
AAAAGCTTGAGACATATCACATGCCAGAAATGGTGTTTGGGGACAGTTGTTTGGCTCTGAAGCATTTTAA
GAGTGGCTTTACAATTCATTTCAATGCATTTGATGCTCTAGTTGGGTGGAAGAAGGAAGCTTTGCCACCA
GTTGAAGTCCCAGCTGCTGCTCAATGGAAATTCCGAAGTAAACCCTCCCAGCAAATAATACTGGACTATG
ATTATACATTCACAACGCCATACTGCGGAAGTGAAACTGTTAAGTTTGATGCAGACAAGCTTGAAGGAGG
AGAGATCTCTAAGGATACCTGCAATCTCCATTGGGAAGACTGCAACGAAAAAATTGATGTAGTTGCTTTG
GCTTCGAAAGAGCCTATTCTCTTTTATGATGAGGTAGTCTTGTATGAAGATGAATTGGCTGATAGTGGAG
TGTCACTTTTAACTGTAAAAGTGAGAGTCATGCCAAGTTGTTGGTTTCTCCTGTTGCGATTTTGGCTTAG
AGTTGATGGAGCACTAATGAGACTAAGGGACACTCGAATGCATTGTGCTTTTAATGACAATGCAAGTCCC
ACTATTCTTCGAGAAAGCTGCTGGAGAGAAGCCACATTTCAGGCTTTATCTGCAAAGGGATACCCTTCTG
AAGATGCTGCCTATAATGATCCAAGCATCATCAGCCAAAGGCTTCCTGTCATCATGCATAAGACCCAAAA
GCTAAAGGTACCTGATAATGTGTAAGGCTGTAAATTGTCTCCATGCAAGAGTTTCTGTTTTGCTGGATTC
TATTGAATGTAAACTTTTCTTTTCACATTCGCATATTCTCTTACTCTTTCTCATGTTTATTAAGTT

>XM_021952533.1 AP-4
GTTGGGTCTAATATACGAACAAAAGCAATCGGGGCCTCAACTCAACAGCTGAGCTTGAGGAACACCCACC
GCATTTCGGACACAGGGAGGAGAAAGAAGTAAGCAAAGCCATGATCTCGCAGTTCTTCGTGCTCTCACAG
CGTGGCGATAACATCGTCTTCCGCGACTATCGCGGTGAAGTACCAAAAGGAAGTGCGGAGATATTTTTCC
GGAAGGTGAAGTTTTGGAAAGAAGATGGGCAAGAGGAAGCACCACCTGTCTTTAATGTGGATGGTGTCAA
CTACTTTCATGTGAAGGTTGTTGGACTATTATTTGTTGCAACCACAAGAGCTAATGTATCGCCATCTCTT
GTCTTGGAACTTCTTCAAAGAATTGCCCGTGTCATCAAAGATTATGTTGGAGTTCTCAATGAAGATTCAA
TAAGGAAGAATTTTGTGCTTGTGTACGAGTTGCTTGATGAAGTCATGGATTTTGGCTTTGTGCAAACAAC
ATCAACTGAACTGTTGAAGTCTTATATCTTTAATGAGCCAATTATTCTTGATTCTGCACGTTTATCATCT
ATTGGCCCTACTGGCCTTTTTATGCAAGGGACAAAGAGAATGCCTGGAACAGCTGTCACAAAATCAGTTG
TTGCAAGTGAGCCTGGCGGTAGGAAGAGGGAGGAAATTTTTGTGGATATAATCGAGAAGATCAGTGTTAC
TTTTAGCTCTAGTGGGTACATACTGACTTCTGAGATTGATGGCACCATTCAAATGAAGAGCTATCTCACT
GGCAATCCAGAAATCCGATTAGCTCTTAATGAGGACCTGAGCATAGGAAGAGGTGGAGGATCGGCATATG
ACTATAGGAGTTCCTTTGGCTCAGGAGCAGTGATACTTGATGATTGTAATTTTCACGAATCTGTACGTCT
TGATAATTTTGAAGTAGACAAAACTCTGACCCTGGTACCCCCTGATGGTGAATTTCCTGTCATGAACTAC
CGTATGACTCAGGAGTTCAAGCCTCCTTTTCGTATTAATGCTTTGATTGAAGAAGCAGGAGCGCTTAAGG
```

CCGAAGTGATTCTGAAAATATTTGCTGAGTTCCCATCAAACATTACTTCAAATACAATTGCTGTACAAAT
GCCGCTACCTAAATATACAATCAGAGCCAGTTTTGAGTTGGAACCGGGAGCAGTTGGGCAAACAACAGAT
TTCAAGGAAGCAAATAAGAGACTTGAATGGGGTTTAAAGAAGATTGTTGGTGGATCTGAACATACACTGC
GTGCCAGACTGACCTTTTCACCGGAAGCACATGGAAATATAACAAAAGAATCCGGTCCAGTTAGCATGAC
GTTTACAATACCTATGTATAATTCTTCAAGACTTCAGGTAAAGTACTTGCAGATAGCAAAGAAATCTGGA
ACTTATAATCCATATCGGTGGGTTAGATATGTCACGCTCGCCAATTCATATGTTGCTCGTATATGATACA
ATAAAGCTTCATATGTTAATGGTATTTCACGGTGGAGGAATTGGAAAGAAGGGTTCTAGTTGCAAGATGT
CTTATGAAACCCTCACTCTTTTCCTTCTTTGGTGGGTCTGGATATTTCTCTATTCTTTTTCAGTGGTTTC
ATTTCTTGACTAACATTAGGCTGTATCGTTGGATTTTTAGTAGGACAATTGAATTATTAAATTGCTCCTT
CTGCAGTGCTTGTTCAATAACTATATAAATGCACTCAAA

>XM_021963054.1 Serine/threonine-protein phosphatase PP2A catalytic subunit
TTCCATTGAACAAAAACATCGCAAAATTCTAATCTGCTTTTTTCATTTGCATCTGGGTTTCATTTGCATT
TCATTTCGCAGCCTGCCTGTCTGCCTGCCTCTCTTCATTATTTCCCTCCCCGCATGTCATTTCCCTCGTC
TTTTCCTACATTCACATAAATACAATTTCAATTTAATTTCCCTTTTTTTCTCCTTTTTTCTTTTAAAACA
AATAAAAGAACACAGAGCGAGGAATGATAGAAGAAAGGTCGAAACTCGACCGATCTCCAAAACGACTCGT
TTCGCTCCACGCGTCGCGATCCGGAACCCCGACCCCAGCGCCGCTTCACAAATCGCTCTCTGCGTCGAGA
TCGCTCCGCCGGCGTTGAGTTCGCGTGCATCGGATTGGATCTGAGAAATCGGCGATCGGATCTGAGGGCG
GCGGTGGTGGAACGATGCCGGCTCACGCGGATCTGGACCGTCAGATCGAGCATCTGATGGAGTGCAAGAC
GTTGCCGGAGGCAGAGGTGAAGACGCTGTGCGAGCAAGCCAGGGCGATCCTTGTCGAGGAGTGGAACGTA
CAGCCGGTGAAGTGCCCCGTTACGGTGTGTGGAGATATTCACGGCCAGTTCTACGACCTCATTGAGCTCT
TTCGGATAGGAGGCAACGCCCCCGACACTAATTACCTTTTTATGGGTGATTATGTAGATCGTGGGTACTA
TTCTGTGGAGACTGTCACACTTCTGGTGGCCCTGAAAGTCCGTTATAGAGATAGAATTACAATTCTCAGA
GGAAATCACGAGAGTCGGCAAATTACTCAAGTGTATGGTTTTTATGATGAGTGCTTGAGAAAATATGGGA
ATGCCAATGTCTGGAAGTTCTTTACCGATTTATTTGATTATCTTCCCCTCACAGCCCTTATTGAGAGTCA
GATTTTCTGTTTGCATGGGGGCCTTTCCCCATCTTTGGACACATTGGACAATATCCGAGCCTTGGATCGT
ATACAGGAGGTTCCACACGAAGGACCAATGTGCGATCTCTTGTGGTCTGATCCAGATGACCGCTGTGGAT
GGGGAATATCTCCACGTGGTGCTGGTTATACATTTGGACAGGATATAGCTGCTCAGTTCAACCATACCAA
TGGACTAAGTCTCATTTCAAGAGCTCATCAGCTTGTCATGGAAGGATACAATTGGTGTCAGGACAAGAAT
GTGGTGACTGTTTTTAGCGCTCCAAACTATTGTTATAGGTGTGGGAATATGGCTGCAATATTGGAAATTG
GGGAGAACATGGACCAGAATTTCCTGCAGTTTGACCCGGCCCCTCGTCAGATTGAGCCTGACAACACACG
CAAGACTCCAGATTATTTTTTGTAATTTCAGTATCTTACAGGGGGCTGTCTGTAATCCATGTTTTCTGCC
ATGTTACTGTAGATGTGTCTTTAAGAGGAGTTGAGCTCCGTTGTTTAAGTTGAGGGTTATGGTCAACATG
ATTCTTTGTTTGGAGTTTTTGTCCCTGCTGCTGCTGCTTTGTTGAATTTCTGAAGAAACTCGTTAGGAGA
ACTGACATTAGTCACCAATGGCCTGTGGCTCTTGTCTTTGTATATTTTTCTGAGAGCAAACGTCAATAAC
ATGGTATATCTCGTTCTGTAATTTTTCCAACTTCAACATAGAAAAGCTGATTTTTCATACATTTTGA

>XM_021976055.1 Actin-7
ATGGCTGATGCTGAGGACATTCAACCCCTTGTCTGTGACAATGGAACTGGAATGGTGAAGGCTGGATTTG
CTGGTGACGATGCACCCAGGGCTGTGTTTCCTTCTATTGTTGGTCGACCACGACACACTGGTGTTATGGT
TGGTATGGGTCAGAAGGATGCCTATGTAGGTGATGAAGCCCAATCTAAGAGAGGTATCCTTACCTTGAAA
TATCCCATTGAACATGGTATAGTAAGCAACTGGGATGACATGGAGAAGATCTGGCATCACACTTTCTACA
ATGAGCTTCGTGTTGCTCCTGAAGAGCACCCAGTTCTTCTTACTGAGGCTCCTCTCAACCCTAAGGCTAA
CAGAGAAAAGATGACGCAGATCATGTTTGAGACATTCAATGTGCCTGCCATGTATGTTGCCATCCAGGCC
GTTCTCTCTCTGTATGCCAGTGGTCGTACAACTGGTATTGTGCTGGACTCTGGTGATGGTGTGAGTCACA
CTGTGCCAATCTATGAAGGTTATGCCCTCCCTCATGCCATTCTTCGTCTGGACCTTGCTGGTCGTGACCT
CACAGATGCCTTGATGAAGATTCTCACTGAAAGAGGGTACATGTTCACCACCACTGCTGAGCGGGAAATT
GTCCGTGATATGAAGGAGAAGCTCGCATATGTTGCCCTGGACTATGAGCAAGAACTTGAGACTGCTAAGA
GCAGCTCTTCGGTAGAGAAGAACTATGAGCTTCCCGATGGCCAAGTAATCACAATTGGAGCTGAGAGATT
CAGGTGCCCAGAAGTTCTCTTCCAACCATCACTCATTGGAATGGAAGCTGCTGGAATTCATGAGACTACC
TACAACTCTATCATGAAGTGTGATGTGGATATTAGGAAAGACCTATATGGAAACATTGTGCTCAGTGGTG
GGTCAACTATGTTCCCCGGTATTGCAGACAGGATGAGCAAGGAGATTACTGCTCTTGCTCCAAGCAGCAT
GAAGATTAAGGTTGTGGCTCCACCAGAGAGAAAGTACAGTGTCTGGATTGGAGGGTCCATCCTTGCATCC
CTCAGTACCTTCCAGCAGATGTGGATTTCCAAGGGTGAGTACGATGAGTCTGGTCCATCCATTGTCCACA
GGAAGTGCTTCTGA

>XM_021951078.1 Polyubiquitin

ATGCAGGTCTTCGTCAAAACCCTAACCGGCAAGACAATCACTCTCGAGGTCGAGAGCTCCGATACCATCG
ACAATGTCAAGGCCAAGATCCAAGACAAGGAGGGAATTCCCCCCGATCAGCAGAGGCTCATCTTCGCCGG
CAAGCAATTGGAGGACGGCAGAACCCTAGCCGACTACAACATCCAGAAGGAATCGACTCTTCACTTGGTT
CTGCGATTGAGGGGTGGTATGCAGATCTTCGTCAAAACCCTAACCGGGAAGACCATAACCTTAGAAGTCG
AGAGCTCTGATACGATCGACAACGTAAAGGCCAAAATCCAAGACAAGGAGGGCATCCCCCCAGACCAGCA
GAGGTTGATCTTCGCCGGCAAGCAATTGGAGGACGGCCGCACCCTTGCCGACTACAATATCCAAAAGGAA
TCGACTTTGCACTTGGTTCTCCGATTGAGAGGCGGTATGCAAATCTTCGTCAAAACCCTAACCGGGAAGA
CAATAACCCTAGAAGTCGAGAGCTCTGATACCATCGACAACGTGAAGGCCAAGATCCAAGATAAGGAGGG
CATCCCCCCAGACCAGCAGAGGTTGATTTTCGCAGGAAAGCAATTGGAGGACGGCCGAACCCTCGCCGAC
TACAACATCCAAAAGGAATCAACTTTGCACTTAGTTCTTCGATTGAGAGGTGGTATGCAGATTTTCGTCA
AAACCCTAACCGGGAAAACAATTACTCTGGAGGTCGAGAGCTCCGATACCATTGATAACGTGAAGGCCAA
GATCCAAGACAAGGAGGGTATCCCCCCAGATCAGCAGAGGCTTATCTTCGCCGGTAAGCAATTAGAGGAC
GGTAGGACCCTTGCGGATTACAACATCCAAAAGGAGTCAACTTTGCACTTGGTTCTGCGTCTGAGAGGTG
GTATGCAGATTTTCGTGAAGACCCTTACAGGGAAGACGATAACTTTGGAGGTCGAGAGCTCTGATACGAT
TGACAATGTGAAGGCGAAGATTCAGGACAAGGAAGGGATTCCTCCAGATCAGCAGAGGCTGATCTTTGCT
GGGAAGCAATTGGAGGATGGAAGGACTCTGGCGGATTACAACATCCAGAAAGAGTCGACTCTTCATCTGG
TGCTCAGGCTTCGTGGTGGCATGCAGATTTTTGTGAAGACCCTGACTGGGAAGACCATTACTCTGGAGGT
TGAGAGCTCTGACACTATTGACAATGTCAAAGCTAAGATTCAAGACAAGGAGGGGATCCCACCGGACCAG
CAGAGGTTGATCTTTGCCGGGAAACAGCTTGAGGATGGTCGTACCCTTGCAGATTATAATATCCAGAACG
AGTCCACTCTCCATCTTGTCCTTCGTCTGCGTGGTGGTGATTTCTGA

>XM_021958703.1 Serine/threonine-protein phosphatase 2A 65 kDa regulatory subunit A beta isoform
ATGTCTATGGTTGATGAACCGCTATACCCAATAGCCGTTCTCATAGACGAGCTGAAAAATGACGACATCC
AGCTACGGTTGAACTCAATCCGCAGGCTATCTACCATTGCACGTGCTCTTGGGGAAGAACGAACCCGTAA
GGAGTTAATCCCCTTTCTGAGCGAAAACAATGATGACGATGATGAGGTACTTCTTGCAATGGCAGAAGAG
TTGGGGGTCTTCATTCCATATGTTGGGGGAGTGGAGCATGCACATGTTTTGCTCCCACCCTTGGAAACCC
TTTGCACTGTCGAGGAAACCTGTGTGAGGGACAAGGCTGTGGAGTCATTATGTAGGATTGGGTCTCAGAT
GAGGGAGGGTGATTTAGTAAACTGGTTTATTCCTCTTGTGAAGAGGTTGGCAGCTGGTGAATGGTTTACA
GCTCGAGTATCTGCATGTGGGCTTTTTCATATTGCCTACCCTAGTGCACCAGAGACATTGAAGACGGAAC
TGCGGTCAATTTACAGTCAGTTGTGTCAAGATGACATGCCTATGGTTAGAAGGTCTGCTGCAACAAACCT
GGGGAAATTTGCAGCAACTGTCGAACCTGCTCATCTGAAGACTGATATCATGTCCATATTTGAGGATCTT
ACACAAGATGATCAAGATTCTGTTCGACTATTGGCTGTTGAGGGCTGTGCTGCTCTTGGAAAGTTGTTGG
AGCCCCAAGACTGTGTTGCACATATCCTCCCTGTTATTGTTAACTTCTCTCAGGATAAGTCTTGGCGTGT
GCGTTACATGGTTGCAAATCAACTATATGAGCTTTGTGAAGCTGTGGGGCCTGAGCCTACGAGGACGGAC
TTGGTTCCTGCTTATGTGCGGTTGCTTCGAGATAATGAGGCTGAAGTACGTATAGCAGCTGCTGGCAAAG
TTACTAAGTTTTGTCGGATTTTAAGCCCAGAACTTGCAATTCAGCATATTCTTCCATGTGTGAAGGAGCT
GTCATCAGATTCTTCCCAACATGTCCGGTCTGCTTTGGCTTCAGTTATAATGGGAATGGCTCTTGTATTA
GGAAAGGATGCTACAATTGAGCAGCTCCTTCCCATTTTTCTTTCGCTTCTGAAGGATGAATTTCCGGATG
TGCGCCTTAATATCATTAGCAAGCTTGATCAAGTGAATCAGGTTATTGGAATCGATTTGCTATCTCAATC
CTTATTACCAGCCATAGTTGAGCTTGCGGAGGATAGACATTGGAGGGTTCGGCTCGCAATTATAGAGTAC
ATACCTTTGTTGGCAAGTCAGCTGGGCGTAGGGTTCTTTGATGATAAGCTTGGTGCCCTTTGCATGCAGT
GGCTACAGGATAAGGTCTACTCAATTCGTGATGCTGCTGCTAACAATTTGAAACGTCTTGCAGAAGAGTT
TGGCCCAGAATGGGCAATGCAGCATATTATTCCACAGGTTCTGGAGATGATTGACAATCCACACTATTTG
TATCGAATGACAATTCTACGTGCAGTCTGTCTCCTTGCCCCGGTTATGGGTTCAGAAATCACATGTTCAA
AACTGCTGCCAGTGGTTGTCAATGCGTCAAAGGACAGAGTTCCCAACATCAAATTTAATGTGGCAAAGGT
GCTGCAGTCCCTTATCCCCATAGTTGATCAGTCTGTGGTGGAGAAGACAATTCGGCCCTCTTTGGTTGAG
CTGAGTGAGGACCCAGATGTTGACGTCCGATTTTTCGCTAACCAGGCACTTCAGGCAATTGATCATGTCA
TGATGTCAAGCTAG

>XM_021953530.1
CTATAAATACACAACCCAAAAGCTCCATTCAACACACACTCAATTGGAGAAGCTTCAGTTTCTCCCCCAT
TTGAAATCTCTCTCACTTTCTTCCAGGAAAAAAACAAAACAAAACAAAAGGAGAGAGATACACATAGTGA
AAATGGGTGTTCCTCTTCTTTCATCACCAGCATTTCCTACACTCTTTCTCTTCTCTCTCATAGCATTGTG
TCTCATTCCTCAGCATGCTCTTGCAAGCATAACGAGGCACTACAAGTTTGAAATCAAACAGCAAAATGTG

ACCAGATTGTGCCACACAAAAAGCATTGTAACAGTAAACGGCCAGTTTCCTGGGCCTCGAATTGTTGCTA
GGGAGGGTGATCGCCTCCTTATTAAAGTGGTTAACCATGTCCAACACAACGTCTCTATCCATTGGCACGG
CGTTCGACAGCTTCGAACAGGGTGGGCTGATGGGCCTGCATATGTGACCCAATGTCCCATACAAACAGGC
AACAGCTATGTGTACAACTTCACCATTGTAGGCCAAACAGGCACTCTTTGGTGGCATGCTCACATTTCAT
GGCTAAGATCAACTCTTTATGGCCCCCTAGTCATCCTTCCCAAGCTTGGTGTGCCTTACCCATTCACTAA
ACCCTACACGGAGGTTCCGATCATCTTCGGAGAGTGGTGGAATGCAGACCCTGAGGCAGTCATTAACCAG
GCCTTGCAAACAGGTGGAGGGCCAAATGTTTCCGATGCTTATACCTTTAATGGACTTCCAGGGCCCTTGT
ACAACTGTTCTGCCAAAGATGCATTCAAGCTAAAGGTGAAATCTGGGAAGACCTACCTTCTCCGCCTAAT
CAATGCAGCACTCAATGATGAGCTCTTCTTCAGCATTGCAAACCACACCCTCAAAGTGGTAGAAGCCGAT
GCCGTTTATGTCAAACCCTTTGAGACTGACACAATTCTCATTACCCCTGGGCAGACTACAAATGTTCTGC
TGAAGACCAAGCCCCATTTCCCAAATGCCACATTTTTCATGACTGCCAGGCCATACGTGACTGGACTAGG
CACCTTTGACAACTCCACTGTTGCTGGTATTTTAGAATACGAATCACCCTCCAAAACCCTCCACTCAACC
CTCCCAATGAAGAAACTTCCACTATTTAAACCAATTCTGCCTGCTCTAAACGACACATCCTTTGCAACAA
AGTTCAGCAACAAACTCCGTAGTTTAGCCAATGCACAATTCCCAGCTAATGTCCCCCAAAAGGTTGACAA
GCACTTTTTCTTCACAGTAGGCCTTGGAACCAACCCCTGCAACAAGCATAACCAAACCTGCCAGGGACCT
AATGGAACAATGTTTGCTGCTTCAGTGAATAACGTTTCTTTTGTAATGCCAACCACTGCTCTACTCCAAG
CCCACTTCTCCGGGCAATCAAATCGGGTTTACAGCTCTAACTTCCCAATCAGTCCAGTGATCCCTTTTAA
CTATACAGGAACCCCACCAAACAATACTATGGTGAGTAATGGTACAAAGCTAGTGGTACTTCCATTTAAC
ACTAGCGTGGAGTTGATCATGCAGGACACAAGCATTCTTGGTGCCGAAAGCCACCCTCTTCATCTGCATG
GATATAATTTCTTTGTTATTGGCCAAGGGTTTGGGAACTTTGACCAAAATAAGGACCCTGCAAACTTCAA
TTTGGTCGACCCTGTTGAAAGAAATACCGTGGGTGTGCCCTCTGGTGGATGGGTCGCTATACGATTTCTA
GCAGATAATCCCGGTGTATGGTTCATGCATTGCCACTTGGAAATCCATACTAGTTGGGGCTTGAAGATGG
CTTGGGTAGTCTTAGATGGAAAGCTTCCCAACCAGAAGTTGCTTCCTCCACCGGCTGATCTTCCAACATG
TTAATGTTGGAATCAACTCAAGCTACATTTTTTGGAAGAGAAACTGCTGTCATTATTTTGTGAACCATTT
TTTATCGCTGACAACTTAAATTTGTCTTATGTATTGGCCAATTAATTAAGTTGAAATAATTCTTGTACTG
ATTCATATGGATTTTTTTTGTTTATTCAAATTTACCCATATGGAGTTGAGTGAAGAAAAAGGGTGAGACC
ACATTCTGTAAAAATTTCAAGTATCCTAATGTAAGAGGCTCAAAATGAAAGTATGGATTCTGTACAATTT
TTCTCCAGGCATATCTGATAGATACTTATCTTTGATTTCTGGGTTATTCTATTTCAACCAATACAAGAAG
TTACAATTTTAAAAAACTCTGCAACAAACAGTTTATACTTTCATAATAACAATGCCTATTTAAAGAAGAA
GAAGAAATATTAATTCATCTCATTGATGCATCTTTAGATGCACTAACATAGGTGTCTATCTACAACCGTA
TCCAAAACCAAACAAAGAAATTAACAAAAA

>XM_021969086.1
CTCACCACCTTCAGCACTAATATCATTCAAACAAAGGTTAAGGGGTTGATTAGCAGCTAATAGCTAGCTT
CGCTAACACAGATATGGAGGCACTCGACAGCATTTTCGGCAACAAAATTCGATGCTCGTTACTCTTAGGC
CTTTGCCTTCTGTTTGCTTCATCAGCAATGTCCTTGGCAGAGCCCAAAACTCACCAACATGACTTTGTTA
TTCAAGCAACACCAGTGAAGAGGCTGTGCAAAATCCAAAACTCCATCACAGTCAATGGGCAATTCCCTGG
ACCAACCTTGGAAGTTAACAATGGTGACACTCTCGTCGTCAAAGTCACCAACAAAGCTCGTTACAACGTC
ACCATCCATTGGCATGGAATTAGGCAAATGAGAACTGGATGGGCAGATGGACCTGAATTTGTGACTCAGT
GCCCAATTAGGCCAGGAGGGAGCTACACCTACAGATTTACAATTCAAGGCCAAGAAGGTACTTTGTGGTG
GCATGCTCACAGCTCATGGCTTAGAGCCACTGTCTATGGAGCACTTATCATTCATCCAAAACAAGGAGAC
TCCTACCCATTCACTAAGCCGAAACGCGAAACTGCCCTTCTTCTTGGTGAATGGTGGAACGCGAACCCCA
TCAACGTTTTGAGGCAGTCAACCCGGACTGGAGGAGCTCCTAATGTGTCTGATGCATACACCATCAATGG
TCAACCTGGTGATCTTTACAATTGCTCGAGCCAAGACACAATCATAGTTCCTATAGACTCCGGTGAGACC
AACCTTCTTAGAGTCATCAACGCTGCCCTCAACCAACCTCTTTTCTTCACTGTGGCAAACCACAAACTGA
CTGTTGTTAGTGCTGACGCCTCCTACACCAAACCTTTCACCACCACAGTCCTCATGCTAGGACCCGGCCA
GACAACCGATGTTTTGATCACCGGTGACCAGCCACCAGCCCGATACTACTTGGCGGCACGTGCCTATTTC
AGTGCACAAAATGCAGCATTTGACAACACCACCACCACGGCCATTCTTGAATACAAGTCTGCCCCTTGCA
GCCCCAACTGCACAAACGGTCCAACAGTTAAACCAATTATGCCACCACTTCCTGCTTTCAATGACACAGC
CACTGCCTCTGCTTTCACCACAAGCTTCAGAAGTCCTAGAAAAGTTGAAGTCCCAACTGAAATCGACGAA
AACCTTTTCTTCACAATCGGCCTTGGACTCAACAACTGCCCAAAGCATTTCAACAAAACCAGAAGGTGCC
AAGGCCCCAATGGAACACGCTTCACAGCCAGCATGAACAATGTGTCCTTTGTGCTTCCAAACAACATCTC
AATCTTGCAGGCCTATCAACAAAACATACCTGGAGTTTTCACTGCTGATTTTCCAGCAAACCCACCGCTG
AAATTCGATTACACGGGCAACGTGAGCCGCTCCCTCTGGCAGCCTCTTTCTGGAACCAGGGGATACAAGT
TGAAGTATGGATCAAGGGTGCAGGTTGTGCTACAAGACACAAGTATCGTCACACCAGAGAACCACCCTAT
TCATCTCCACGGATACGATTTCTACATCCTTGCAGAGGGTTTTGGAAATTTCAATGCCCAGACTGACACC

AAAAAATTCAACCTTGTTGATCCACCTATGAGGAACACAGTGGCAGTGCCTGCGAATGGATGGGCAGTCA
TCAGATTCGTAGCTGACAATCCAGGGGCCTGGATAATGCATTGTCACTTGGATGTTCATATCAACTGGGG
TTTGGCCATGGTCTTCTTGGTTGACAATGGAGTTGGGACACTGCAGTCGATTGAGGCTCCTCCGGCGGAT
CTGCCTCTGTGTTAAGAATCTAAAGATCACCATAGATATGCTACCAATGCTAGCAACTCAGAAACCCAAC
TATGGGGTTTTCATTTTTCCCGTCTCAGTTATATGATTCCATTGTTGTTTGTATTTCCCCTGCATTTGGG
GGTCTAGTTCTTGCTCTGTTTCATTGTTCTTTTTTGATACATAAAGCGGGCTTAGAGGAGATGGATGGCG
CTGCGGTTGCAAAACCGAGTACCATCTGATTTAATCACAGGCAGGCAGACAGACATGTTTCTTGGTTCAT
AGATGTTTTATATTTCGTAAGCAAGTGTTAAAGAATTGTTTTGAAGTTAATGAAATGTTTTTTCTTATTA
CTCCTCTGTTTCTTAGAATCCCCTGTTTCTTAGATTCCCCTGTTTCTTGTATTCCTCTGTTTCATCTATG
AAAAAGATA

>XM_021964966.1
CACAGCATTACGACATAGAACACACAAAAAAAACTGAGGGTCTCAGAGAGAGAGAGAGAGAGAGAGAGAG
AGAGAGAGAGAGAGAGAGAGCTATAAGGATCAAGAGAGAGAAAAATGGAGATGGTGCCTTCGATTCGTGC
CTTGCTTTTGGTGGCTTGTTTGTTGTTTCCAGCATCAGTGGAATGCATGGTTCGACATTACAAGTTCAAT
GTGGTGCAAAAGAAAACCTCAAGATTATGTTCAAGCAAGCCCATTGTCACAGTCAATGGAAGGTACCCTG
GGCCCACCATATATGCTAGGGAAGATGACACAGTGCTGATCAAGGTGGTCAACCACGTCAAATACAATGT
TAGCATCCATTGGCATGGAGTCCGACAACTGCGGACGGGTTGGGCCGATGGGCCGGCGTACATAACCCAA
TGTCCAATTCAACCGGGTCAAAACTATGTGTACAACTTCACCATCACTGGGCAAAGGGGCACACTTTGGT
GGCATGCACACATCCTGTGGCTCAGGGCCACAGTCCATGGCGCCTTGGTCATCTTACCCAAGCTTGGGGT
CCCATACCCATTTCCTGCACCCCAGAAAGAAGTGGTTGTAATCTTAGGTGAATGGTGGAAATCAGATGTG
GAATCTGTCATTAATCAGGCTTTGAAATCTGGCTCAGCCCCGAATGTCTCTGATGCTCACACAATTAATG
GCCACCCGGGCCCTCTCTCCACCTGCTCCTCACAAGGAGGGTTTAAATTACCAGTAAGACCTGGCAAGAC
ATACATGCTAAGAATCATCAATGCTGCGCTCAATGAAGAGCTCTTCTTCAAGATTGCCAGCCACAAACTA
ACCGTGGTTGAGGTTGATGCTGTGTACACAAAACCCTTCAAAACTGACACCGTTCTGATAGCCCCAGGGC
AGACCACAAACGTTCTTCTGTCAACCAACCATGGCACAGGCAAGTACTTGGTTGCAGCCTCACCTTTCAT
GGACAATCCAAACATCCTTGTAGACAACAAAACTGGCACTGCTACTTTGCACTATACGGGCACCCTCGAA
AGCACTAGGACCACCCTCACAGCCCCACCTCCTCAAAATGCCACACTAGTTGCAACAAAGTTTACAAATT
CCCTCAGAAGCCTGAATTCAGTCAAGTTTCCTGCTAGGGTTCCATTGAAAATTGACCACTCTCTTTTGTT
CACTGTTGGCCTTGGCCTTCACACTTGTGCTTCATGTGCCAATGGCAACAGATTGGTGGCAAATATCAAT
AATGTCACATTTGTGATGCCCAAAATCTCACTTCTTCAAGCACATTTCTTCAACATAAGTGGAGTTTTTA
CCGATGATTTTCCCGGAAACCCTCTGAATCCTTATAATTATACGGGTCCACAGCCGACGAATATGCAGAC
CATGAAGGGGACTAGGCTTTATAGACTTGCTTATAACTCTACAGTGCAGCTTGTGTTGCAAGACACTGGG
ATGATTGCCCCTGAAAACCATCCAGTTCATCTTCATGGGTTTAATTTCTTTGAGGTTGGCAGGGGATTGG
GGAATTTCAACCCAAAGAAGGATCCAAAAAGATTTAATCTTATTGACCCTGTTGAGAGGAACACAATAGG
AGTGCCATCTGGTGGATGGACTGCTATTAGATTCAGGGCTGATAATCCAGGAGTTTGGTTTATGCATTGC
CATCTTGAAGTGCACACAACATGGGGGCTTAAGATGGCATTTGTGGTGGACAATGGCAAAGGCCCAAATG
AATCTGTTCTACCACCTCCAAGCGACCTTCCCAAGTGTTAGCTTTATATGTAAAGAATTCCACCACAAAT
TGTTTGCCAACTCAATTCTCAACTCATAGCAATTCAAGGAACAAGAGCTAATAGGAAGAATTCTTTGGAG
CAAAGCTAAAGGGAAAGCTGCTTCTACATGTTGAATAAAAGGGAATTGGGCTTCTTTTTAAGATTAGCTC
GCTGGGTCGTATATCTTTTTC

>XM_021958888.1
AACAACAACAACAACAATACCAAGACAAACAATTAAGAACAGAGAGATCTTTCAGTAGTGTTACTATTGC
AGAGAGAAACACAGAGAGAGAGAGAGAGAGAGAGAGAGAGAGCAGTGATAAATGGAGTCGTGGGTTCGATTT
CTCGTGCTTTTTCTGGCGTGCCTTCTTCCGGGTTTGGTTGAGAGCAGAATCCGACACTACAAGTTTAATG
TGGTCTTGAAAAATACTACCAGACTATGCTCCACCAAGCCAATCGTAACCATTAACGGTCGGTTCCCGGG
GCCAACCCTCTATGCCAGGGAAGACGACACGGTACTCGTCAAAGTTACCAACCATGTCAAGTATAATGTT
TCCGTTCACTGGCATGGTGTCCGGCAACTGCAAACCGGTTGGGCAGATGGTCCAGCATACATCACACAGT
GCCCAATTCCCCCAGGCCAAAGCTATGTGTACAACTTCACCATCACAGGGCAAAGAGGCACACTTCTTTG
GCATGCACATATTCTCTGGCTGAGGGCCACAGTCCATGGTGCCCTTGTAATTTTGCCCAAGCGTGGTGTG
CCTTATCCATTCCCAGCACCCCACAAGGAAGTAAATGTCATATTAGCTGAATGGTGGAAATCAGATACTG
AATCAGTGATCAATCAAGCCATGAGTTCTGGTTTAGCACCAAATGTCTCGGATGCTCACACCATTAACGG
CCATCCAGGCCCCGTTGCAAATTGTTCTTCCCAAGGCGGCTTTACATTACCTGTGGAAAGCGGCAAGACC
TACCTGCTACGAATCATCAATGCTGCGCTCAATGAGGAGCTCTTCTTCAAAATTGCGGGACACAAATTGA
CCATAGTGGAAGTAGATGCTACCTATGTGAAACCATTCAAGACAGACACCATTGTGATTGCCCCAGGGCA

AACCACCAATGCCCTCATCACGGCGAATCAAAAGTCCGGCAAGTACTTGGTTGCAGCCTCACCATTCATG
GACTCTCCCATTGCTGTCGACAACCTCACTGCCACTGCAACTCTGCATTATTCAGGCACACTTGCAACCA
CATCCACAACTCTCACCAACCCACCTCCCCAAAACGCAACCCAAGTTGCTAACAATTTTATCAACTCCCT
CAAAAGCTTAAATTCCAAGAAATTTCCAGCCAAAGTCCCATTGAAAATTGACCACAACCTCTTGTTCACA
GTTGGGCTCGGGATCAACCCGTGCCCTACTTGCAAAGCAGGTAATGGGAGCAGAGTTGTGGCAAGTGTCA
ACAATGTCACATTTGTTATGCCAACAACTGCTCTTCTTCAAGCTCATGTTTTCAACATCAGTGGGGTTTT
TACTACCGATTTTCCCGGAAACCCGCCAAATACCTTCAACTTTTCTGGGGGTCCACCTGCAAATGCGAGC
ATGGCAACCACAAATGGGACAAAGCTTTATAGGCTGGCTTATAACTCGACTGTTCAACTTGTTTTGCAAG
ACACTGGGACTATAGCCCCTGAGAACCACCCTGTCCATTTACATGGATACAATTTCTATTCTGTGGGCAG
GGGAGTGGGGAATTACAATCCCAAGAAGGATCCTAAGAAGTTTAATCTTGTTGATCCTGTTGAAAGGAAC
ACAGTTGGAGTGCCATCCGGCGGATGGGCAGCTATCCGATTTCAAGCAGATAATCCAGGGGTGTGGTTCT
TGCATTGCCATCTTGAAGTGCACACGACATGGGGGCTCAAGATGGCATTCCTGGTGGACAATGGAAAGG
ACCGAATGAATCAGTTCTACCACCTCCGAGCGATCTCCCAAAATGTTAATGTAGCCAAAAGGGGCCTCTA
GTCTTGCAAGTAGAGAGAACTAACAAAAAAGGAGACGCGCGGAATCAAAGGCACATGATCAAGAAGAAGA
AGAAAGAGCGGATGAATAAACAAACAAATTTATATATATATATATTTCTAGATTCAAACTTATTTCTTGA
GTGACTGAAAAAATTTCCTCGAGCAAAGTTTGAGTTAATTTTTGGTTGTTGAAGTGAAGCATACCACATT
TTTTTTTAAAGAATTAATATATGTATTGGATCTTGGATGTGCAAGTCTTTGTTTATCTATTATAAAGTTT
GTGAAATGGATTTTTAAATTTCTTTTTCA

>XM_021978785.1
TGGGGTTCTTCTGCACTTCTCTTCTTATGCTTAGTTGGGTTCTTTTCCACTCTGGCAAAAGCCTCTTTGA
AGACATACCAATTCGATGTTCAAGTGAAGAATGTGAGCAGGTTGTGCCATTCTAAGCCAATTGTTACAGT
AAATGGGATGTTCCCTGGACCTACAGTTTATGCTAGAGAAGGAGATACGCTTCTCGTCAATGTTACCAAC
CACGCACAATACAATATGTCAATTCATTGGCATGGATTAAAGCAATATAGAAATGGGTGGGCAGATGGGC
CTGCCTATATCACCCAATGTCCTATCAAGACAGGACATAGTTACACCTACAATATCACTATAACAGGGCA
AAGAGGAACTCTATGGTGGCATGCACATATTTTCTGGCTAAGAGCCACTGTGTATGGAGCAATTGTCATC
TTGCCTAAACAAGGGACTGGTTTTCCATTTCCTCAGCCATACAAGGAGGCTAATATTGTCTTAGGAGAAT
GGTGGAATAACGACGTCGAAGAGGTTGTTAAACAAGGAAACAAACTGGGATTGCCACCAAATATGTCAGA
TGCACACACCATCAATGGGAAGCCAGGGCCTCTCTTTCCCTGTTCTGAAAAACATACCTATGCATTGGAG
GTTGAACAGGGAAAGACGTACCTGCTACGAATCATCAATGCTGCACTCAATGATGAGCTATTCTTTGCCA
TTGCTGGTCACAACTTGACAGTGGTAGAAATTGATGCAGTCTACACCAAACCATTCACATCTCAAGCAAT
ACTAATAGCACCAGGCCAGACCACGAATGTTCTCGTTCAAGCCAACCAAGCGCCCGGAAGATATTTCATG
GCGTCTAGGCCATTCATGGACGCACCGATTTCCATTGACAACAAAACTGCCACTGGAATACTGCAGTACA
AAGGCATCCCAAATACTGTGCAGCCAGTTCTTCCCCAACTTCCGGCACTCAATGACACAGCTTTTGCATT
GAGCTTCAATGCCAAGCTAAGAAGTTTAAACACAGCACAGTTCCCGGCCAGTGTACCTCTTAAAGTTGAT
CGACATCTCTTTTACACGATCGGGTTGGGAATCAACCAATGCACGACTTGCCTCAATGGAACACAGCTCA
CTGCTTCTTTAAACAATATCACATTTGTGATGCCCCAAATTGGGCTGCTTCAAGCTCATTATTTCAACAC
CAAAGGAGTGTTTACCACAGATTTCCCAGACCGCCCTCCAACACCTTTCAATTACACTGGTGCACCACTC
ACTGCCAACCTTGGCACTAAAAGAGGCACCCGGCTTAGCAAACTTGCCTTCAATTCAACAGTTGAGTTGG
TGCTACAAGACACCAATCTTCTCACAGTGGAGTCCCATCCTTTCCACCTTCATGGTTACAATTTCTTTGT
TGTTGGGACCGGAGTTGGGAACTTCGACCCCAAAAAGGACCCGGCAAAGTATAACTTGGTGGATCCTCCC
GAAAGAAACACAATTGGAGTTCCTACCGGTGGTTGGGTTGCCATAAGGTTCAGGGCTGATAATCCAGGTG
TCTGGTTCATGCACTGTCATTTGGAGCTCCATACCAGCTGGGGTTTGAAGACTGCATTCGTGGTGGAAAA
TGGGAAAGACTCAGATCACTCTATCTTGCCTCCACCTACAGACCTTCCACCTTGTTAGTCCCTCGAACAG
CAGT

>XM_021977624.1
AGGTCTCTACCTGAGACCGAAAACCGCACAACACTTCAAGGGCGACCTGAAGAACACACTAGGAAAAATC
GAGATATTTTCTGGCCAAGACCCGAAAATGGAGTTTAAGAAAATGAGTTGGGTCTTAGCCTTTGTAGGGT
TTATGTTCTTGGGTGGGATTTGCAAGGCTAATGGACATACCCATTATTATGATTTTGTTCTGACAGAATC
GAATTTTACAAGGCTTTGCGAAACAAAGAGCATCTTAACTGTGAACGGGACTTTGCCGGGGCCGACCATC
ACTGTCCGGAAGGGAGACACTGCTTATGTGAATGTCCACAACCAAGGCTCATACGGTCTCACCCCTTCATT
GGCATGGAGTGAAGCAGCCACGAAATCCATGGTATGATGGACCAGAGAACGTAACGCAGTGCCCTATTCA
AGCAGGGTCAAACTTCACTTACTGGATTATCTTCTCCACTGAAGAAGGAACATTATGGTGGCATGCTCAT
AGCGATTGGACACGTGCCACCGTCTATGGTGCCATCATTATTCTACCTGCTCTCAACACAACTTATCCTT
TTACAACACCAGATGCAGAGGAAACACTGGTTCTAGGATCCTGGTACAAGGGAGATGTGAATGAAATTAT
TGAAAATGCCCTCGCAACAGGTGGCGATCCCAACGTTTCAGATGCCTTCACCATCAACGGAGAGCCTGGA

GATTTGTATAAGTGTTCCAATGCAACAACATACCGTTGGGTGGTTGATTATGGCAAGACGTATCTTCTCC
GCTTAATCAACGCGGTGTTGAATGAAGAAATGTTCTTCGCCATTGCCAACCACAACCTCACAGTGGTAGC
TCAAGACGCTGCATACATAAAACCTATAACCACCTCCTACCTCATGATAACTCCAGGCCAAACCATGGAC
ATTCTGGTAGTTGCAAACCAGTCTCCCAGCTCCTATCACGCAGCTTCCGCTCCTTTTGTTGATGGGGATG
TTGCATTCAACAACAGCACCACCACTGCCATTCTTCAATACAACGGCAGCACCACCCCCTCAACCATTCC
CACTCCGACTTTTCCCAATCCCGCCGACGAAACGGCTGCCTCAAACTTCACTACGCAAGTGAGGGCCTTG
GCAAGCAAAGACTACCCAATTAGTGTCCCGTTAAACATCACACACACGCTCTTCATCTCTGTTTCCGTAA
ATGAAAGAATTTGTCCCAATTCTTCTTGTGATGGGCCAGACGGAAATGCACTTGCTGCAAGCTTAAACAA
CATCAGTTTTGTGACTCCATCGATTGATATATTGCAAGCCTATTCCGGGGCAATTTATGGAGTTTACTCA
GCTAATTTCCCACACAAACCTTATATTTTCAACTTCACGGGACACGTGCGAAACGACACAATATATCCTA
ACTTTGGGACGAAGGTGAGGACTATTGAATATGGTGAAGAAGTTGAGATAATCTATCAAGGGACCAACAT
GATTGCCGCTGAGAACCATCCGATGCATCTCCATGGTTTTAGCTTCTATTTGGTTGGAACTGGTTCTGGG
AATTTCGACCCTAATCAGGCCTATAAGACCTACAATTTGGTTGATCCACCAGAAGTAAACACCATTGGTG
TACCAAAGAATGGATGGGCAACCGTCAGATTTAAAGCTGATAATCCTGGAGTATGGTTTATGCACTGTCA
TTTGGAAAGGCACGCCAGCTGGGGAATGGCCACTGTACTCATTGTGACAAATGGAAACACCAATGAAACC
AGCATGCTCCCAGCACCTGCTTATATGCCTCCTTGTACTTAATAAAGTTTGTAATTCACTTTCTCTTTCA
TGGATCTCAGTTTATATATTTATACTGAGACTTTGATTTTATTAAGAATAATTGTAGCATCCAAGTTTCT
TTAATGTTTGATTATCTTTTTTAAGTGAATATAGTATTGGGGGCTTCTTTTTTTAG

FASTA sequences of the LCMO protein sequences used for the phylogenetic analysis

>Tver-AAL07440.1
MSRFHSLFAFVVASLTAVAHAGIGPVADLTITNAAVSPDGFSRQAVVVNGGTPGPLITGNMGDRFQLNVI
DNLTNHTMLKSTSIHWHGFFQKGTNWADGPAFINQCPISSGHSFLYDFQVPDQAGTFWYHSHLSTQYCDG
LRGPFVVYDPNDPAADLYDVDNDDTVITLVDWYHVAAKLGPAFPLGADATLINGKGRSPSTTTADLSVIS
VTPGKRYRFRLVSLSCDPNYTFSIDGHNMTIIETDSINTAPLVVDSIQIFAAQRYSFVLEANQAVDNYWI
RANPNFGNVGFTGGINSAILRYDGAAAVEPTTTQTTSTAPLNEVNLHPLVATAVPGSPVAGGVDLAINMA
FNFNGTNFFINGASFTPPTVPVLLQIISGAQNAQDLLPSGSVYSLPSNADIEISFPATAAAPGAPHPFHL
HGHAFAVVRSAGSTVYNYDNPIFRDVVSTGTPAAGDNVTIRFRTDNPGPWFLHCHIDFHLEAGFAVVFAE
DIPDVASANPVPQAWSDLCPTYDARDPSDQ

>Lch-ADB97327.1
MGLVSRFLRLMFLGCLLFCEAEGAVHYYDFVVKESNFTRLCNTKSMLTVNDSFPGPEIRVQKGDTAFVTV
YNQGPYGITIHWHGVKMPRNPWSDGPEYVTQCKIAPRTNFTQEINFSIEEGTIWWHAHSDWSRATVHGAI
IVYPASGTTYPYPTPDGEQTIVLASWYNSDVMEVYEEAVASGEEFNTSDAFTINGQPGALYDCSTGTTFR
MNVTSGKTYLLRIINAILNEEMFFGIANHNLTVVGTDGFYTKPINAEYIFITPGQTIDVLVTANQTPSYY
YMAASPFSDSEADFDNSTTVAYFQYIGNETVPDPDPIPFPSLPGTNDSGPPFGFIRQLRSLADAAXVSVP
TNIPKHIYMTASVNVIYCPNDSCSANLGGDKLGASLNNQSFQFPSIDILQAYYNNISGVFTTDFPLEPPS
FFNFTAVEPNVTVYAGQGTKVIELDYGDEVELVFQGTNLGNAQNHPMHLHGYSFYMVGLGFGNFNSSTDP
SSYNLVDPPEVNTIQLSKRGWVAIRFKADNPGVWLMHCHYERHTAWGMAAVFIVKDGGTTNTSMRAPPAY
MPSCSSS

>ATLAC1-At1g18140
MENLGFLIISTFLLLFTTLLPYSSASTTRRFHFNVEWKKVTRLCHTKQLLTVNGQYPGPTVAVHEGDIVE
IKVTNRIAHNTTIHWHGLRQYRTGWADGPAYITQCPIRSKQSYTYRFKVEDQRGTLLWHAHHSWQRASVY
GAFIIYPRQPYPFSGSHIQSEIPIILGEWWNDDVDNVEKAMMKTGAGAKVSDAYTLNGLPGPLYPCSTKD
TFTATVDAGKTYILRIINAALNNELFVAVANHTLTVVEVDAVYTKPVHTKAIMIAPGQTTTLLLRADQLS
GGEFLIAATPYVTSVFPFNNSTTVGFIRYTGKTKPENSVNTRRRRRLTAMSTVVALPNMLDTKFATKFSD
SIKSLGSAKYPCKVPTKIDKRVITTISLNLQDCPLNQTCDGYAGKRFFASMNNISFVRPPISILESYYKK
QSKGVFSLDFPEKPPNRFDFTGVDPVSENMNTEFGTKLFEVEFGSRLEIVFQGTSFLNIENHPLHVHGHN
FFVVGRGFGNFDPEKDPKRYNLVDPPERNTFAVPTGGWAAIRINADNPGVWFIHCHLEQHTSWGLAMGFI
VKDGPLPSQTLLPPPHDLPQC

>ATLAC2-At2g29130
MVTWVLNYLLVAFLFAISYNIDAASAGITRHYQFDIQLKNITRLCKTKTIVTVNGKFPGPRVTAREGDNL
QIKVVNHVSNNISIHWHGIRQLRSGWADGPSYVTQCPIRMGQSYVYNFTVTGQRGTLWWHAHIQWMRATV

YGPLIIILPKLHQPYPFPKPYKQVPILFGEWFNADPQAVVQQALQTGAGPNASDAHTFNGLPGPLYNCSTK
DTYKLMVKPGKTYLLRLINAALNDELFFTIANHTLTVVEADACYVKPFQTNIVLLGPGQTTNVLLKTKPI
YPNATFYMLARPYFTGQGTIDNTTVAGILQYQHHTKSSKNLSIIKPSLPPINSTSYAANFTKMFRSLASS
TFPANVPKVVDKQYFFAIGLGTNPCPKNQTCQGPTNTTKFAASINNVSFILPNKTSLLQSYFVGKSKNVF
MTDFPTAPIIPFNYTGTPPNNTMVSRGTKVVVLKYKTTVELVLQGTSILGIEAHPIHLHGFNFYVVGQGF
GNFNPARDPKHYNLVDPVERNTINIPSGGWVAIRFLADNPGVWLMHCHIEIHLSWGLTMAWVVLDGDLPN
QKLLPPPSDFPKC

>ATLAC3-At2g30210
MESFRRFSLLSFIALLAYFAFLASAEHHVHQFVITPTPVKRLCRTHQSITVNGQYPGPTLVVRNGDSLAI
TVINRARYNISIHWHGIRQLRNPWADGPEYITQCPIRPGQTYTYRFKIEDQEGTLWWHAHSRWLRATVYG
ALIIYPRLGSPYPFSMPKRDIPILLGEWWDRNPMDVLKQAQFTGAAANVSDAYTINGQPGDLYRCSRAGT
IRFPIFPGETVQLRVINAGMNQELFFSVANHQFTVVETDSAYTKPFTTNVIMIGPGQTTNVLLTANQRPG
RYYMAARAYNSANAPFDNTTTTAILQYVNAPTRRGRGRGQIAPVFPVLPGFNDTATATAFTNRLRYWKRA
PVPQQVDENLFFTVGLGLINCANPNSPRCQGPNGTRFAASMNNMSFVLPRSNSVMQAYYQGTPGIFTTDF
PPVPPVQFDYTGNVSRGLWQPIKGTKAYKLKYKSNVQIVLQDTSIVTPENHPMHLHGYQFYVVGSGFGNF
NPRTDPARFNLFDPPERNTIGTPPGGWVAIRFVADNPGAWFMHCHIDSHLGWGLAMVFLVENGRGQLQSV
QAPPLDLPRC

>ATLAC4-At2g38080
MGSHMVWFLFLVSFFSVFPAPSESMVRHYKFNVVMKNVTRLCSSKPTVTVNGRYPGPTIYAREDDTLLIK
VVNHVKYNVSIHWHGVRQVRTGWADGPAYITQCPIQPGQVYTYNYTLTGQRGTLWWHAHILWLRATVYGA
LVILPKRGVPYPFPKPDNEKVIVLGEWWKSDTENIINEALKSGLAPNVSDSHMINGHPGPVRNCPSQGYK
LSVENGKTYLLRLVNAALNEELFFKVAGHIFTVVEVDAVYVKPFKTDTVLIAPGQTTNVLLTASKSAGKY
LVTASPFMDAPIAVDNVTATATVHYSGTLSSSPTILTLPPPQNATSIANNFTNSLRSLNSKKYPALVPTT
IDHHLFFTVGLGLNACPTCKAGNGSRVVASINNVTFIMPKTALLPAHYFNTSGVFTTDFPKNPPHVFNYS
GGSVTNMATETGTRLYKLPYNATVQLVLQDTGVIAPENHPVHLHGFNFFEVGRGLGNFNSTKDPKNFNLV
DPVERNTIGVPSGGWVVIRFRADNPGVWFMHCHLEVHTTWGLKMAFLVENGKGPNQSILPPPKDLPKC

>ATLAC5-At2g40370
MDVTKSLLCFISFVAFLLFSSVAEANKAHHHEFIIQATKVKRLCETHNSITVNGMFPGPMLVVNNGDTLV
VKVINRARYNITIHWHGVRQMRTGWADGPEFVTQCPIRPGSSYTYRFTIQGQEGTLWWHAHSSWLRATVY
GSLLVFPPAGSSYPFTKPHRNVPLLLGEWWDANPVDVLRESIRTGGAPNNSDAYTINGQPGDLYKCSSQD
TTVVPINVGETILLRVINSALNQPLFFTVANHKLTVVGADASYLKPFTTNVIVLGPGQTTDVLITGDQPP
NRYYMAARAYQSAQNAPFGNTTTTAILQYKSAPCCGVGGGSGTKKGNSFKPIMPILPAYNDTNTVTRFSQ
SFRSLRRAEVPTEIDENLFVTIGLGLNNCPKNFRSRRCQGPNGTRFTASMNNVSFALPSNYSLLQAHHHG
IPGVFTTDFPAKPPVKFDYTGNNISRSLYQPDRGTKLYKLKYGSRVQIVLQDTGIVTPENHPIHLHGYDF
YIIAEGFGNFNPKKDTAKFNLEDPPLRNTVGVPVNGWAVIRFIADNPGVWIMHCHLDAHISWGLAMAFLV
ENGNGVLQTIEQPPHDLPVC

>ATLAC6-At2g46570
MTSSAVPSLFRLSFLLFTLQVMNIGRIGAATRFYQFKVQTIRLTRLCQTNEIVTVNKKFPGPAISAQEDD
RIVIKVINMTPYNTTIHWHGIKQKRSCWYDGPSYITQCPIQSGQSFTYNFKVAQQKGTFLWHAHFSWLRA
TVYGPLIVYPKASVPYPFKKPFNEHTILLGEYWLKNVVELEQHVLESGGPPPPADAFTINGQPGPNYNCS
SKDVYEIQIVPRKIYLLRLINAGINMETFFTIANHRLTIVEVDGEYTKPYTTERVMLVPGQTMNILVTAD
QTVGRYSMAMGPYESAKNVKFQNTSAIANFQYIGALPNNVTVPAKLPIFNDNIAVKTVMDGLRSLNAVDV
PRNIDAHLFITIGLNVNKCNSENPNNKCQGPRKGRLAASMNNISFIEPKVSILEAYYKQLEGYFTLDFPT
TPEKAYDFVNGAPNDIANDTQAANGTRAIVFEYGSRIQIIFQNTGTLTTENHPIHLHGHSFYVIGYGTGN
YDQQTAKFNLEDPPYLNTIGVPVGGWAAIRFVANNPGLWLLHCHFDIHQTWGMSTMFIVKNGKKVQESLP
HPPADLPKC

>ATLAC7-At3g09220
MEGVRVPIACALILLAISSITSASIVEHTFNVQNLTVSRLCKRQVITVVNGSLPGPTIRVKEGDSLVIHV
LNHSPHNITIHWHGIFHKLTVWADGPSMITQCPIQPGQRYAYRFNITGQEGTLWWHAHASFLRATVYGAL
VIRPKSGHSYPFPKPHKEVPILFGEWWNTDVVALEEAAIATGVPPNNSDAYTINGRPGNLYPCSKDRMFS

LNVVKGKRYLLRIINAAMNIQLFFKIANHRLTVVAADAVYTAPYVTDVIVIAPGQTIDALLFADQSVDTS
YYMAAHPYASAPAVPFPNTTTRGVIHYGGASKTGRSKPVLMPKLPSFFDTLTAYRFYSNLTALVNGPHWV
PVPRYVDEEMLVTIGLGLEACADNTTCPKFSASMSNHSFVLPKKLSILEAVFHDVKGIFTADFPDQPPVK
FDYTNPNVTQTNPGLLFTQKSTSAKILKFNTTVEVVLQNHALIAAESHPMHLHGFNFHVLAQGFGNYDPS
RDRSKLNLVDPQSRNTLAVPVGGWAVIRFTANNPGAWIFHCHIDVHLPFGLGMIFVVKNGPTKSTTLPPP
PPDLPKC

>ATLAC8-At5g01040
MPRLHHYLSNQAFLVLLLFSSIASAAVVEHVLHIQDVVVKPLCKEQIIPAANGSLPGPTINVREGDTLVV
NVINNSTYNVTIHWHGVFQLKSVWMDGANMITQCPIQPGYNFTYQFDITGQEGTLLWHAHVVNLRATLHG
ALVIRPRSGRPYPFPKPYKEVPIVFQQWWDTDVRLLQLRPAPVSDAYLINGLAGDSYPCSENRMFNLKVV
QGKTYLLRIVNAALNTHLFFKIANHNVTVVAVDAVYSTPYLTDVMILTPGQTVDALLTADQAIGKYYMAT
LPYISAIGIPTPDIKPTRGLIVYQGATSSSSPAEPLMPVPNDMSTAHRFTSNITSLVGGPHWTPVPRHVD
EKMFITMGLGLDPCPAGTKCIGPLGQRYAGSLNNRTFMIPERISMQEAYFYNISGIYTDDFPNQPPLKFD
YTKFEQRTNNDMKMMFPERKTSVKKIRFNSTVEIVLQNTAIISPESHPMHLHGFNFYVLGYGFGNYDPIR
DARKLNLFNPQMHNTVGVPPGGWVVLRFIANNPGVWLFHCHMDAHLPYGIMSAFIVQNGPTPETSLPSPP
SNLPQCTRDPTIYDSRTTNIDLSY

>ATLAC9-At5g01050
MPRVHHSLSNQAFLVLLLFSSIASAAIVEHVLHVKDVVVTPLCKEQMIPIVNGSLPGPTINVREGDTLVV
HVINKSTYNVTIHWHGVFQLKSVWMDGANMITQCPIQPSNNFTYQFDITGQEGTLLWHAHVVNLRATIHG
ALIIRPRSGRPYPFPKPYKEVPLIFQQWWDTDVRLLELRPAPVSDAYLINGLAGDSYPCSKNRMFNLKVV
QGKTYLLRIINAALNTHLFFKIANHNVTVVAVDAVYTTPYLTDVMILTPGQTIDAILTADQPIGTYYMAI
IPYFSAIGVPASPDTKPTRGLIVYEGATSSSSPTKPWMPPANDIPTAHRFSSNITSLVGGPHWTPVPRHV
DEKMFITMGLGLDPCPSNAKCVGPLDQRLAGSLNNRTFMIPERISMQEAYFYNITGVYTDDFPDQPPLKF
DFTKFEQHPTNSDMEMMFPERKTSVKTIRFNSTVEIVLQNTGILTPESHPMHLHGFNFYVLGYGFGNYDP
IRDARKLNLFNPQMHNTVGVPPGGWVVLRFIANNPGIWLFHCHMDAHLPLGIMMAFIVQNGPTRETSLPS
PPSNLPQCTRDPTIYDSRTTNVDMSY

>ATLAC10-At5g01190
MVFPIRILVLFALLAFPACVHGAIRKYTFNVVTKQVTRICSTKQIVTVNGKFPGPTIYANEDDTILVNVV
NNVKYNVSIHWHGIRQLRTGWADGPAYITQCPIKPGHSYVYNFTVTGQRGTLWWHAHVLWLRATVHGAIV
ILPKLGLPYPFPKPHREEVIILGEWWKSDTETVVNEALKSGLAPNVSDAHVINGHPGFVPNCPSQGNFKL
AVESGKTYMLRLINAALNEELFFKIAGHRFTVVEVDAVYVKPFNTDTILIAPGQTTTALVSAARPSGQYL
IAAAPFQDSAVVAVDNRTATATVHYSGTLSATPTKTTSPPPQNATSVANTFVNSLRSLNSKTYPANVPIT
VDHDLLFTVGLGINRCHSCKAGNFSRVVAAINNITFKMPKTALLQAHYFNLTGIYTTDFPAKPRRVFDFT
GKPPSNLATMKATKLYKLPYNSTVQVVLQDTGNVAPENHPIHLHGFNFFVVGLGTGNYNSKKDSNKFNLV
DPVERNTVGVPSGGWAAIRFRADNPGVWFMHCHLEVHTTWGLKMAFLVENGKGPNQSIRPPPSDLPKC

>ATLAC11-At5g03260
MKMGFLFLFCYLLAFLGYSPVDAAVKKYQFDVQVKNISRICNAKPIVTVNGMFPGPTVYAREGDRVIINV
TNHVQYNMSIHWHGLKQYRNGWADGPAYITQCPIQTGQSYLYDFNVTGQRGTLWWHAHILWLRATVYGAI
VILPAPGKPYPFPQPYQESNIILGEWWNKDVETAVNQANQLGAPPPMSDAHTINGKPGPLFPCSEKHTFV
IEAEAGKTYLLRIINAALNDELFFGIAGHNMTVVEIDAVYTKPFTTKAILLGPGQTTNVLVKTDRSPNRY
FMAASPFMDAPVSVDNKTVTAILQYKGVPNTVLPILPKLPLPNDTSFALDYNGKLKSLNTPNFPALVPLK
VDRRLFYTIGLGINACPTCVNGTNLAASINNITFIMPKTALLKAHYSNISGVFRTDFPDRPPKAFNYTGV
PLTANLGTSTGTRLSRVKFNTTIELVLQDTNLLTVESHPFHLHGYNFFVVGTGVGNFDPKKDPAKFNLVD
PPERNTVGVPTGGWAAIRFRADNPGVWFMHCHLEVHTMWGLKMAFVVENGETPELSVLPPPKDYPSC

>ATLAC12-At5g05390
MTTVHTFSILLFFCSLFSASLIIAKVQHHDFVIQETPVKRLCKTRNAITVNGMFPGPTLEVNNGDTLEVK
VHNRARYNITIHWHGVRQIRTGWADGPEFVTQCPIRPGKSYTYRFTIQGQEGTLWWHAHSSWLRATVYGA
LIIHPTPGSSFPFPKPDRQTALMLGEWWNANPVDVINQATRTGAAPNISDAYTINGQPGDLYNCSTKETV
VVPINSGETSLLRVINAALNQPLFFTVANHKLTVVGADASYLKPFTTKVLMLGPGQTTDVLLTADQPPKR
YYIAARAYQSAQNAPFDNTTTTAILQYKKTTTTSKPIMPVLPAFNDTNTVTSFSRKFKSLRNVVVPKTID
DNLFFTIGLGLDNCPKKFPKSRCQGLNGTRFTASMNNVSFVLPSNFSLLQAHSNGIPGVFTTDFPSKPPV

KFDYTGNNISRALFQPVKGTKLYKLKYGSRVQVVLQDTNIVTSENHPIHLHGYDFYIVGEGFGNFNPKKD
TSKFNLVDPPLRNTVAVPVNGWAVIRFVADNPGVWLMHCHLDVHIKWGLAMAFLVDNGVGELETLEAPPH
DLPIC

>ATLAC13-At5g07130
MEQLRPFFLLLAIFVASLVNAEVHFHEFVIQETPVKRLCRVHNSITVNGQFPGPTLEVRNGDSLVITAIN
KARYNISLHWHGIRQMRNPWADGPEYITQCPIQPGGSYTYRFTMEDQEGTLWWHAHSRWLRATVYGALII
RPPLSSPHYPFPVIPKREITLLLGEWWDRNPMDVLNLAQFTGAAPNISDAFTINGQPGDLYRCSSQETLR
FLVGSGEIVLLRVINSALNQELFFGVANHKLTVVAADASYTKPFSTNVIMLGPGQTTDVLLTADQPPAHY
YMAAHAYNSANAAFDNTTTTAILKYKDASCVTLQAKSQARAIPAQLPGFNDTATAAAFTAQMKSPSKVKV
PLEIDENLFFTVGLGLFNCPTPNTQRCQGPNGTRFTASINNVSFVFPKQNSIMQAYYQGTPTGVFTTDFP
PTPPVTFDYTGNVSRGLWQPTRGTKAYKLKFNSQVQIILQDTSIVTTENHPMHLHGYEFYVVGTGVGNFN
PNTDTSSFNLIDPPRRNTIGTPPGGWVAIRFVANNPGAWLMHCHIDSHIFWGLAMVFLVENGEGHLQSVQ
SPPLDLPQC

>ATLAC14-At5g09360
MEFKLNIPNTIIKTLQTIVFFLFVLLAFQIAEAEIHHHTFKIKSKAYTRLCNTNKILTVNGEFPGPTLKA
YRGDKLIVNVINNANYNITLHWHGARQIRNPWSDGPEYVTQCPIRPGESYVYRIDLKVEEGTIWWHAHSQ
WARATVHGAFIVYPKRGSSYPFPKPHREIPLILGEWWKKENIMHIPGKANKTGGEPAISDSYTINGQPGY
LYPCSKPETFKITVVRGRRYLLRIINAVMDEELFFAIANHTLTVVAKDGFYLKHFKSDYLMITPGQSMDV
LLHANQRPNHYFVAARAYSSAFGAGFDKTTTTAILQYKGDTLNRIKPILPYLPPYNRTEASTRFTNQFRS
QRPVNVPVKINTRLLYAISVNLMNCSDDRPCTGPFGKRFSSSINNISFVNPSVDILRAYYRHIGGVFQED
FPRNPPTKFNYTGENLPFPTRFGTKVVVLDYNSSVELILQGTTVWASNIHPIHLHGYNFYVVGSGFGNFD
RRKDPLRYNLVDPPEETTVGVPRNGWTAVRFVANNPGVWLLHCHIERHATWGMNTVFIVKDGPTKSSRMV
KPPPDLPSC

>ATLAC15-At5g48100
MSHSFFNLFLISLFLYNNCIAHHYTFTVREVPYTKLCSTKAILTVNSQFPGPIIKVHKGDTIYVNVQNRA
SENITMHWHGVEQPRNPWSDGPEYITQCPIRPGSDFLYKVIFSIEDTTVWWHAHSSWTRATVHGLIFVYP
RPPQILPFPKADHEVPIILGEWWKRDVREVVEEFVRTGGAPNVSDALTINGHPGFLYPCSKSDTFHLTVE
KGKTYRIRMVNAAMNLPLFFAIANHSLTVVSADGHYIKPIKATYITISPGETLDMLLHADQDPERTYYMA
ARAYQSGNIDFNNSTTIGILSYTSSCKAKTSSFSGYYPTLPFYNDTSAAFGFFTKIKCLFSGQVPVQISR
RIITTVSINLRMCPQNSCEGPNGSRLAASMNNISFVTPSHVDILKAYYYHIKGVYGTRFPEFPPLIFNFT
AENQPLFLETPRLATEVKVIEFGQVVELVIQGTSLVGGGLDHPMHLHGFSFYVVGVGFGNYNISEEDPSS
RYNLYDPPYKNTMTVPRNGWIAIRFVADNPGVWFMHCHLDRHQTWGMNVVFIVKNGREPNQQILPPPDDL
PPCYE

>ATLAC16-At5g58910
MEIPRRFCICVLTFFVFVLLSPTTVHSIIRHYKFNVMTNTTKLCSSKPIVTVNGQFPGPTIVAREGDTIL
IKVVNHVKYNVSIHWHGIRQLRTGWADGPAYITQCPIQPGQNYLHNFTLTGQRGTLWWHAHILWLRATVH
GAIVILPKLGVPYPFPKPYKEKTIVLSEWWKSDVEELINEASRIGTAPSASDAHTINGHSGSISNCPSQS
SYGLPVRAGKTYMLRIINAALNEELFFKIAGHVLTVVEVDAVYTKPYKTDTVFIAPGQTTNVLLTANANA
GSNYMVAATTFTDAHIPYDNVTATATLHYIGHTSTVSTSKKTVLASLPPQNATWVATKFTRSLRSLNSLE
YPARVPTTVEHSLFFTVGLGANPCQSCNNGVRLVAGINNVTFTMPKTALLQAHFFNISGVFTDDFPAKPS
NPYDYTAPVKLGVNAATMKGTKLYRLPYNATVQIVLQNTAMILSDNHPFHLHGFNFFEVGRGLGNFNPEK
DPKAFNLVDPVERNTVGVPAGGWTAIRFIADNPGVWFMHCHLELHTTWGLKMAFVVDNGHGPDQSLLPPP
ADLPKC

>ATLAC17-At5g60020
MALQLLLAVFSCVLLLPQPAFGITRHYTLEIKMQNVTRLCHTKSLVSVNGQFPGPKLIAREGDQVLIKVV
NQVPNNISLHWHGIRQLRSGWADGPAYITQCPIQTGQSYVYNYTIVGQRGTLWYHAHISWLRSTVYGPLI
ILPKRGVPYPFAKPHKEVPMIFGEWFNADTEAIIRQATQTGGGPNVSDAYTINGLPGPLYNCSAKDTFRL
RVKPGKTYLLRLINAALNDELFFSIANHTVTVVEADAIYVKPFETETILIAPGQTTNVLLKTKSSYPSAS
FFMTARPYVTGQGTFDNSTVAGILEYEPPKQTKGAHSRTSIKNLQLFKPILPALNDTNFATKFSNKLRSL
NSKNFPANVPLNVDRKFFFTVGLGTNPCNHKNNQTCQGPTNTTMFAASISNISFTMPTKALLQSHYSGQS
HGVYSPKFPWSPIVPFNYTGTPPNNTMVSNGTNLMVLPYNTSVELVMQDTSILGAESHPLHLHGFNFFVV
GQGFGNFDPNKDPRNFNLVDPIERNTVGVPSGGWAAIRFLADNPGVWFMHCHLEVHTSWGLRMAWLVLDG

DKPDQKLLPPPADLPKC

>XP_021803024.1
MECLIQHYGLFVVMLMILAGALPSGFSQTTRRFQFNVEWKNVTRLCSTKPLLTVNGEYPGPTIAVHEGDN
VEIKVTNHIADNTTIHWHGIRQLRTGWADGPAYITQCPIRGGKSYTYKFTVQYQRGTLWWHAHYAWQRAS
VYGAFIIHPRMPFPFSAPIQDEFPIIFGEWWNGDVDEVENEMMKYGGGPNSSDAYTINGLPGPFYPCSNK
DTFIKTVERGKTYLLRIINAALNDELFFAVVNHTLTVVEIDAVYTKPFTATAIMVAPGQTTNVLLTANQV
PDSSGTFLMAAWPYLTSIFPFDNSTTAGFLRYKNTVTENIKHLQKMPSTFEPNKYNLPKMEDTKFVTKFS
DKLRSLASPQYPCTVPKTIDKRVVTVVSLNLQDCPANKTCKGYEGKRFFASMNNLSFVRPSMSILESYYN
KLRNNEYSTDFPEKPLKPFDYTGVDTVSENMNTQFGTKILEVPYGTNLEIVLQGTSFLNVENHPIHIHGH
NFFIVGRGFGNFNVAKDPAKYNLIDPPERNTVAVPTGGWAAIRFKADNPGVWFIHCHLEEHTSWGLASGL
IVQNGPRASQCVLPPPKDLPSC

>XP_021833014.1
MESCRLHLRAMLLLVAFLYPAFVQSLVRHYKFSVVFKNTTKLCSSKPTITVNGKFPGPTLYAREDDTVIV
RVINHVNHNLTIHWHGVKQLGTVWADGPAYITQCPIQPGQNFIYNFTFTGQRGTLLWHAHTSWLRATLHG
AIVILPKRGTPYPFPTPDEEKTVILAEWWKSDVEAVVNQSVQSGLPPNVSDAHTINGHAGPVPGCSSPGG
YTLHVESGKTYLLRIINAALNDDLFFKIAGHNLTVVEVDASYTKPFQTDTIFISPGQTTNAILTANQGIG
KYLIAASPFMDAPVGFDNLTSIASLRYKGTPANPKAFLTSIPPQNATPVTNTFMNALRSLNSKQYPANVP
LTIDHSLFFTIAVGVNPCATCVNGSKLVAAFNNVSFVMPTIALLQAYYYNKKGVYTLDFPANPPIPFNYT
GNSTANMQTTNGTRLYRLGYNSTVQIVLQGTAIIAPESHPTHLHGFNFFVVGKGIGNFDPKKDPKTFNLV
DPIERNTISVPTGGWTAIRFRADNPGIWFLHCHLEVHTTWGLKMAFLVENGKGPNETLRPPPADLPTC

>XP_021829870.1
LQTCHCITIQSRKMVRTKSFCWGSSALLFLCLVGFFSTPAKAALKTYRFDVQVKNVSRLCHSKPIVTVNGMFPGPTV
YAREGDTLLVNVTNHAQYNMSIHWHGLKQYRNGWADGPAYITQCPIKTGHSYTYNITITGQRGTLWWHAHIFWLRAT
VYGAIVILPKQGTGFPFLQPYKEANIVLGEWWNNDVEEVVKQGNKLGLPPNMSDAHTINGKPGPLFPCSEKHTYALE
VEQGKTYLLRIINAALNDELFFAIAGHNLTVVEIDAVYTKPFTSQAILIAPGQTTNVLVQANQVPGRYFMAARPFMD
APVSIDNKTATGILQYKGIPNTVQPVLPQLPALNNTAFALSFNAKLRSLNTAQFPASVPLKVDRHLFYTIGLGINQC
TTCHNGTQLTASLNNITFVMPQIGLLQAHYFNTKGVFTTDFPDRPPTPFNYTGAPLTANLGTKLGTRLSKLAFNSTV
ELVLQDTNLLTVESHPFHLHGYNFFVVGTGVGNFDPKKDPAKYNLVDPPERNTIGVPTGGWVALRFRADNPGVWFMH
CHLELHTSWGLKTAFVVENGKDSDHSVLPPPTDLPPC

>XP_021833685.1
MASSFWNRFFCGLVFLCFGFLGLITSPAEAAIKNYQFDVQVKNVSRLCHAKSIVTVNGRFPGPTIYVREGDRVVINV
TNHAQYNMSIHWHGVKQYRNGWADGPAYITQCPIQTGSSYTYDFNVTGQRGTLWWHAHILWLRATVHGAIVIMPKQG
TPFPFPQPHREAEIILGEWWNVDVEEFVKKANNLGLAPNTSDAHTINGKPGPLFPCSEKHTFVMEVEQGKTYLLRII
NAALNDELFFAIAGHNLTVVEVDAVYTKPFTTQAILIAPGQTTNVLVQANQASGRYFMATRPFMDAPVPIDNKAATG
IFQYRGIPNTVLPSLPQLPASNDTSFALSYGKKLKSLNTPNFPANVPLKVDRKLFYTIGFGKEPCPTCLNGTRFVAS
LNNISFEMPQVGLLQAHYFNLKGVFKTDFPDRPQTPFNYTGAPLTANLGTSTGTRLSKIAFNSTVELVLQDTNLLTV
ETHPFHLHGYNFFVVGTGIGNFDPAKDPAKYNLVDPVERNTVGVPTGGWTAIRFRADNPGVWFMHCHLELHTGWGLK
TAFVVEDGPGSDQSVLPVPKDLPPC

>XP_017179926.1
MASICLRNRFLCAFMFIFFEFLGLITSLAEAAIKKYQFDYSVHGFQIDNILFGILQVQVKNVSRLCHAKP
IVTVNGRFPGPTIYVREGDRLQINVTNHAQYNMSIHWHGLKQYRNGWADGPAYVTQCPIQTGSSYTYDFN
VTGQRGTLWWHAHILWLRATVYGAIVIMPKQGTPFPFAQPYRETKIILGEWWNVDVETFVNKANSLGLPP
NSSDAHTINGKPGPLFPCSEKHTFAMEVEQGKTYLLRIINAALNDELFFAIAGHNLTVVEVDALYTKPFT
TQAILIAPGQTTNVLVKANQASGRYFMAARPFMDAPVPVDNKTATAIFQYRGVPNTVLPSLPQLPNPNDT
SFALSYNKKLRSLNSPNFPANVPLKVDRKLFYTIGFGKESCPSCINGTRFVASLNNISFEMPQVGLLQAH
YFNLKGVFKTDFPDRPSTVFNYTGAPLTANLGTSTGTRLSKIAFNSTVELVLQDTNLLTVESHPFHLHGY
NFFVVGNGIGNFDPAKEPAKYNLVDPVERNTVGVPTGGWTAIRFRADNPGVWFIHCHLELHTGWGLKTAF
VVEDGPGSDQSVLPPPKDLPPC

>XP_021833835.1
MASSVRNRLFAGLLFLCFGFLGFITSPAEAAIKKYQFDVQVKNVSRLCHAKPIVTINGRFPGPTIYVREG
DRVLINVTNHAQYNMSIHWHGLKQYRNGWADGPAYVTQCPIQTGSSYTYDFNVTGQRGTLWWHAHILWLR

ATVYGAIVILPKQGTPFPFPRPYREAEIILGEWWNGNVEEFVNKANNLGLPPNSSDAHTINGKPGPLFPC
SEKHTFAMEVEQGKTYLLRIINAALNDELFFGIAGHNLTVVEVDAVYTKPFTTEAILIAPGQTTNVLVQA
NQASGRYFMAARPFMDAPVAIDNKTATGIFQYRGIPNIVLPSLPQLPASNDTAFALSYNKKLKSLNTPNF
PANVPLKVDRKLFYTIGFGKESCPTCLNGTRFVASLNNISFEMPQVGLLQAHYFNLKGVFKTDFPDRPQT
PFNYTGTPLTASLGTSTGTRLSKIVFNSTVELVLQDTNLLTVESHPFHLHGYNFFVVGTGIGNFDPAKDP
AKYNLVDPVERNTVGVPTGGWTAIRFRADNPGVWFMHCHLELHTGWGLKTAFVVEDGPGSDHSVLPPPKD
LPPC

>XP_008246156.1
MASSRRNRLFSGLLFLCFGFLGFITSPAEAAIKKYQFDVQVKNVSRLCHAKPIVTINGRFPGPTIYVREG
DRVLINVTNHAQYNMSIHWHGLKQYRNGWADGPAYVTQCPIQTGSSYTYDFNVTGQRGTLWWHAHILWLR
ATVYGAIVILPKQGTPFPFPQPYREAEIILGEWWNGNVEEFVNKANNLGLPPNSSDAHTINGKPGPLFPC
YEKHTFVME

>XP_007198990.1
MAKTNSFCWGSSALLFLCLVGFFSTPAKAALKTYQFDVQVKNVSRLCHSKPIVTVNGMFPGPTVYAREGD
TLLVNVTNHAQYNMSIHWHGLKQYRNGWADGPAYITQCPIKTGHSYTYNITITGQRGTLWWHAHIFWLRA
TVYGAIVILPKQGTGFPFLQPYKEANIVLGEWWNNDVEEVVKQGNKLGLPPNMSDAHTINGKPGPLFPCS
EKHTYALEVEQGKTYLLRIINAALNDELFFAIAGHNLTVVEIDAVYTKPFTSQAILIAPGQTTNVLVQAN
QVPGRYFMAARPFMDAPVSIDNKTATGILQYKGIPNTVQPVLPQLPALNNTAFALSFNAKLRSLNTAQFP
ASVPLKVDRHLFYTIGLGINQCTTCLNGTQLTASLNNITFVMPQIGLLQAHYFNTKGVFTTDFPDRPPTP
FNYTGSPLTANLGTKLGTRLSKLAFNSTVELVLQDTNLLTVESHPFHLHGYNFFVVGTGVGNFDPKKDPA
KYNLVDPPERNTIGVPTGGWVALRFRADNPGVWFMHCHLELHTSWGLKTAFVVENGKDSDHSVLPPPTDL
PPC

>XP_021834477.1
MFPGPTVYAREGDTLLVNVTNHAQYNMSIHWHGLKQYRNGWADGPAYITQCPIKTGHSYTYNITITGQRG
TLWWHAHIFWLRATVYGAIVILPKQGTGFPFPQPYKEANIVLGEWWNNDVEEVVKQGNKLGLPPNMSDAH
TINGKPGPLFPCSEKHTYALEVEQGKTYLLRIINAALNDELFFAIAGHNLTVVEIDAVYTKPFTSQAILI
APGQTTNVLVQANQAPGRYFMASRPFMDAPISIDNKTATGILQYKGIPNTVQPVLPQLPALNDTAFALSF
NAKLRSLNTAQFPASVPLKVDRHLFYTIGLGINQCTTCLNGTQLTASLNNITFVMPQIGLLQAHYFNTKG
VFTTDFPDRPPTPFNYTGAPLTANLGTKRGTRLSKLAFNSTVELVLQDTNLLTVESHPFHLHGYNFFVVG
TGVGNFDPKKDPAKYNLVDPPERNTIGVPTGGWVAIRFRADNPGVWFMHCHLELHTSWGLKTAFVVENGK
DSDHSILPPPTDLPPC

>XP_021824778.1
MEALDSIFGNKIRCSLLLGLCLLFASSAMSLAEPKTHQHDFVIQATPVKRLCKIQNSITVNGQFPGPTLE
VNNGDTLVVKVTNKARYNVTIHWHGIRQMRTGWADGPEFVTQCPIRPGGSYTYRFTIQGQEGTLWWHAHS
SWLRATVYGALIIHPKQGDSYPFTKPKRETALLLGEWWNANPINVLRQSTRTGGAPNVSDAYTINGQPGD
LYNCSSQDTIIVPIDSGETNLLRVINAALNQPLFFTVANHKLTVVSADASYTKPFTTTVLMLGPGQTTDV
LITGDQPPARYYLAARAYFSAQNAAFDNTTTTAILEYKSAPCSPNCTNGPTVKPIMPPLPAFNDTATASA
FTTSFRSPRKVEVPTEIDENLFFTIGLGLNNCPKHFNKTRRCQGPNGTRFTASMNNVSFVLPNNISILQA
YQQNIPGVFTADFPANPPLKFDYTGNVSRSLWQPLSGTRGYKLKYGSRVQVVLQDTSIVTPENHPIHLHG
YDFYILAEGFGNFNAQTDTKKFNLVDPPMRNTVAVPANGWAVIRFVADNPGAWIMHCHLDVHINWGLAMV
FLVDNGVGTLQSIEAPPADLPLC

>XP_021819124.1
MEFNKMSWVLAFLGFMFLGGICKADGATHFYDFVLKETNFTRLCSTKSILTVNGSLPGPTITVQKGDTAF
VNVHNQGLYGLTMHWHGVKQPRNPWSDGPENITQCPIPVGTNFTYEINFSTEEGTLWWHAHSDWTRATVY
GAIIILPADNTTYPFATPDAQETLILGSWFKGDVMEIIEDALATGGEPNISDAFTINGQPGDLYTCSNET
TYRWLVDSGNTYLLRVINAVMNEEQFFAIADHNLRVVAQDAAYIKPITTPYIMITPGQTMDILVTANQPP
SHYYIASTSFVDGNVTFNNSTTTAILQYNGNYSTPSTTPFPTLPDHDDDTAAENFTKQVRALASEDHPIS
VPLNISTSMYIAVSINERICANSSCAGPDGNALSASLNNISFQTPSISVLQAYYGGNTNGVYATNFPDIP
PYFYNFTGDFANNTLYPSFGTRVRMINYGEGVEIVFQGTSVIAAENHPMHLHGFSFYLVGTGSGNFNSTT
SPTTYNLVDPPEVNTIGVPKNGWAAIRFVADNPGVWFMHCHLERHSSWGMDTVLIVRNGNTSESTIRGPP
AYMPPCSNS

>XP_007201720.2
MGFYNKMSWVLAFLGFMFLGGISNANGEIHHYDFVLKETNFTRLCSTKSILTVNGSLPGPTITVRKGDTA
FVNVHNQGLYGLTIHWHGVKQPRNPWSDGPENVTQCPIPAGTNFTYEVIFSNEEGTLWWHAHSDWTRATV
YGAIVILPALNTTYPFATPDAQETLVLASWFKGDVVEIIEEALASGGDPNISDAFLINGQPGDLYDCSNE
TTYRLLVDYGKTYLLRVINAVMNEEQFFAIANHTLTVVAQDAAYIKPITTSYIMITPGQTMDILVTANQP
LSHYYIASKAFVDGVVPFNNSTTTAIVQYNGNYSAPSTPSFPTLPDYDNQTAADNFTSQIRALASEEHPI
SVPLEIHSKIIISIAINERICPDSNCSGPHNNAISASLNNISFELPSIDILQAYYGGNNNGVYRTSFPNK
PPYYFNFTGDVGNNTLYPNFGTRVRMIKYGEGVEIVFQGTNIIAPENHPMHLHGFSFYVVGTGYGNFNST
TSPKTYNLVDPPEVNTIGVPKNGWAAIRFVADNPGVWFMHCHLERHSSWGMDTVLIVRNGNTTESKIRPP
PPAMAGGRRKKKKMEIKDTNFTKLCSTKSALTVNESFPGPIIRAQKGDTVYVNVYNEGRYGVTLHWHGIK
QPRNPWFDGPEYITQCPIQPGTNFTYQILLSSEEGTLFWHAHSDWSRATVHGAFVILPANGTSYPFPKPD
GEQVILFASWYKEDVMTLLDETLKSGGLTTSSDSYTINGEPGDFYLCSNETTYRMSVDYGKTYLLRIVNS
VQNVDMFFAIADHNLTVVGADGAYVKPIVTSYIMITPGQTMDVLVTAKQSLGLYYMLASPYYDGEADDFD
KSMASAIFQYNGNYTPPSSPIYPTYIPGYYDIDSARKFVTQFRSLASAEHPVDVPLNVTTRMFITISIGM
LHCPNNSCAGPEGNRIASGLNNISFADPAVDVLQAYYRNISGYYDASFPDEPPNLFNFTAEDLTTDNYTI
TSRATRVKMLDYNATVEIIFQGTNIMDSGENHPVHLHGFRFYVIGSGLGNFNNVTDPLTYNLVDPPEVNT
FPVPKDGWATIRFIANNPGVWFMHCHFDRHMSWGMDTVFIVKNGGTNETSIRPPPDYLPACSKNSLFGAD
QSMLQMGE

>XP_021820472.1
MLSGKRNSALQILSLLLLFLHCPASSHNYHFVVKEATYTRLCSTKEILTVNGKFPGPVLQAHKGDTIYVN
VHNNGRYNITIHWHGVKQPRYPWSDGTEYITQCPIQPGHKFKQNIIFSEEEGTIWWHAHSDWSRATVYGA
IIVYPKRGASYPFPQPHEEVPIILGQWWKSDVMEVFEEFVRTGGDPNVSDAHTINGQPGDLYPCSKSETF
KLFVDENKAYLLRIINAAMNSILFFSIANHNLTVVGADGSYTKPVTRDYITISPGQTLDALLLTNQQVGQ
YYMAARAYSSSPTIAFDNTTTTAIVQYNNRNSTPFSSPPILPYLPYYNDTNAAFNFFDSLRSLANEDHPI
DVPKNITTRLISTVSVNTFPCPNNSCEGPNGTRLAASMNNISFVDPTTIDILEAYYYHINGVFREGFPDF
PPLVYNFTSEDLPLILQTPKRGTKVKVFDYGSIVECVFQGTSLVAGIDHPMHLHGFSFYIVGTGFGNFDQ
DKNPLNYNLVDPPHRNTVTVPINGWTTIRFKVKNPGVWFLHCHLERHLTWGMNTVFIVKNGKHNKEKLLP
APQEMPPC

>XP_021826148.1
MANGIVHFAFSVFQLFLIFDAGNGKTHRHNFVVKSSSYTRLCSTKDILTVNGQFPGPSLKAHRGDKMIIK
VYNKANYNITFHWHGVKQPRNPWSDGPEYITQCPIRPGNKYTYKIEFTTEEGTMWWHAHSGWARATVHGA
ILVYPKPGSHYPFSKPYAEVPIILGEWWKKNVMEIPRNANITGGEPILSDAYTINGKPGHLYPCSKSGTF
EMTVKHGKTYLLRIISAVMDEELFFGIANHKMILVGRDGSYTKQIETSYIMIAPGQSMDVLLQANQPPSF
YFMAARAYSSAIGAGFDKTMTTAILKYQASSHEPPKFPHYKFPHLPPYDRTQASTDFTKRIRSLATKDHP
TNVPLHVNTHLFFTISVNLLNCSNKPCTGPFGKRFAASVNNISFVAPSIDILQAYYYKIPGVFENDFPKK
LLNFXRKPPKEFNYTGEDLPENLLTPSYGTKVVVLEYNASVELVLQGTNVLASDNHPVHLHGYSFYVVGW
GFGNFNPKKDPLSYNLVDPPEETTVGVPKNGWVAIRFRVDNPGVWLMHCHIERHQTWGMTVVLLVKNGIS
PQNKILLPPHDLPTC

>XP_007227301.1
MANGIVHFAFSVLQLFLIFDAANGKTHRHNFVVKSSSYTRLCSTKDILTVNGQFPGPSLKAHRGDKMIIK
VYNKANYNITFHWHGIKQPRNPWSDGPEYITQCPIRPGNKYTYKIEFTTEEGTMWWHAHSGWARATVHGA
ILVYPKPGSHYPFSKPYAEVPIILGEWWKKNVMEIPRNANITGGEPILSDAYTINGKPGHLYPCSKSGIF
EMTVEHGKTYLLRIISAVMDEELFFGIANHKMILVGRDGSYTKQVVTSYIMIAPGQSMDVLLQANQPPSF
YFMAARAYSSAIGAGFDKTMTTAILKYQASYHEPPEFPHYKFPHLPPYDRTQASTDFTKRIRSLATKDHP
TNVPLHVNTHLFFTISVNLLNCSNKPCTGPFGKRFAASVNNISFVAPSIDILRAYYYKIPGVFENDFPKK
PPMEFNYTGEDLPENLLTPSYGTKVVVLEYNASVELVLQGTNVLASDNHPVHLHGYNFYVVGWGFGNFNP
KKDPLNYNLVDPPEENTVGVHKNGWVAIRFRADNPGVWLMHCHIERHQTWGMTVVFLVKNGNSPQNKILP
PPHDLPTC

>XP_021827255.1
MGKYNNMSWVLAFVGFMLLGGICKANGAIYNYDFVLREENFTKLCTTKSILTVNGSFPGPTITVRKGDTA
FVNVHNQGLYGLTIHWHGVKNPRNPWSDGPENITQCPIPAGTNFTHEVIFSSEEGTLWWHAHSDWTRATV
HGAIIILPALNTSYPFVTPDAQETLILGSWFKGDVNEIIEEALATGGDPNISDAFTINGEPGDLYDCSNE
TTYRWLVDYGKTYLLRVINAVMNEEHFFAIANHNLTVVAQDAAYIKPITTSYVMITPGQTMDILVTANQS

PSHYYIASTSFVDGDVAFNNSTTTAILQYNGNYSAPSTIPLPNLPDHDDDTAAANFTTQVRALASEEHPI
SVPVNISHRLFITVSINERICPNSSCDGPDNNALAASLNNISFVTPSIDILQAYYGGINGVYTPNFPDKP
PYFFNFTGLVQNNTIYPSFATKVKMIKYGKEVEIIFQGTSMIAAENHPMHLHGFSFYLVGTGSGNFNSTT
SPKTYNLVDPPEVNTIGVPKKGWAAIRFVADNPGVWYMHCHLERHSSWGMNTVLIVRNGNTTETSILPPP
AYMPPCSKS

>XP_021809222.1
MGVPLLSSPAFPTLFLFSLIALCLIPQHALASITRHYKFEIKQQNVTRLCHTKSIVTVNGQFPGPRIVAR
EGDRLLIKVVNHVQHNVSIHWHGVRQLRTGWADGPAYVTQCPIQTGNSYVYNFTIVGQTGTLWWHAHISW
LRSTLYGPLVILPKLGVPYPFTKPYTEVPIIFGEWWNADPEAVINQALQTGGGPNVSDAYTFNGLPGPLY
NCSAKDAFKLKVKSGKTYLLRLINAALNDELFFSIANHTLKVVEADAVYVKPFETDTILITPGQTTNVLL
KTKPHFPNATFFMTARPYVTGLGTFDNSTVAGILEYESPSKTLHSTLPMKKLPLFKPILPALNDTSFATK
FSNKLRSLANAQFPANVPQKVDKHFFFTVGLGTNPCNKHNQTCQGPNGTMFAASVNNVSFVMPTTALLQA
HFSGQSNRVYSSNFPISPVIPFNYTGTPPNNTMVSNGTKLVVLPFNTSVELIMQDTSILGAESHPLHLHG
YNFFVIGQGFGNFDQNKDPANFNLVDPVERNTVGVPSGGWVAIRFLADNPGVWFMHCHLEIHTSWGLKMA
WVVLDGKLPNQKLLPPPADLPTC

>XP_021809160.1
MGVPLISSPPFLTLFLFSFMMLCLSPQHALASITRHYKFDIKLQNVTRLCQTKSIVTVNGQFPGPRIVAR
EGDQLLIKVINHVQNNISIHWHGIRQLRTGWADGPAYVTQCPIQTGHSYVYNFTIVGQRGTLWWHAHISW
LRSTVYGPLIILPKLGVPYPFTKPYKAVPIIFGEWWNADPEAVISQALQTGGGPNVSDAYTLNGLPGPLY
NCSAKDAFKLKVEAGKTYLLRLINAALNDELFFSIANHTLQVVEADAVYVKPFETHTILIAPGQTTNVLL
KTKPSFPNATFFLTARPYVTGLGTFDNSTVAGILEYEFPPNTKTLKMKKLPLFKPDLPALNDTSFATKFN
DKLRSLNNAPFPANVPQKVDKHFFFTVGLGTNPCSQHNQTCQGPNGTMFAASINNISFAMPNTALLQSHF
SGQSNGVYTPDFPTNPIIPFNYTGAPPNDTMVSNGTKLVVLPFNTSVELVMQDTSILGAESHPLHLHGFN
FFVVGQGFGNFDRNKDPAKFNLVDPVERNTVGVPSGGWVAIRFLADNPGVWFMHCHLEIHTSWGLKMAWV
VLDGKLPNQKLLPPPADLPKC

>XP_021814279.1
MAFAFPLSPTFVAAFLLALSCFWSLPEFSAAAASGVTRHYRFDIKLKNVTRLCQTKSIVAVNGKFPGPRI
IAREGDRILVKVVNHVQNNISLHWHGIRQLQSGWADGPSYITQCPIQTNQSYVYNFTIIGQRGTLFWHAH
ISWLRATVYGPLIILPKHNASYPFPKPHKEVPIIFGEWWKADPEAVIRQALQTGAGPNVSEAYTINGLPG
PLYNCSKKDTFRLKVKPGKTYLLRLINAAVNDELFFSIANHSLTVGEADALYVKPFKTDILLITPGQTTN
VLLRTKPSYPNATFLMLARPYFTGMGTFDNSTVAGILEYKNPSNPSASTSLKNPPLLKPTLPQINATSFV
ANFSAKFRSLANSKFPANVPKTVRKRFFFTVGLGTNPCPKNQTCQGPNNSTKFAASINNISFILPTTALL
QSHFFGKSNGVFTTDFPTNPVQPFNYTGTPSNNTNINVSNGTKAVVLKFNTSVEVVLQGTGILGAESHPL
HLHGFNFFVVGQGFGNFNPNKDPASFNLVDPVERNTIGVPSGGWVAIRFLADNPGVWLMHCHFDVHLSWG
LRMAWVVQDGKLPNQKLPPPPSDLPKC

>XP_021825503.1
MGSPHFPCFTSMAALLFGFFVAASWMPEFAAGKTRHYTFNIKYHNVTRLCNTRSILSVNGKFPGPRLVAR
EGDQVLIKVVNHVSNNITIHWHGIRQLQTGWADGPAYITQCPIQTGQAYTYNFTITGQRGTLLWHAHISW
LRSTLYGPIIILPKRNESYPFVKPYKEVPIILGEWFNVDPEAVISQALQTGGGPNVSDAYTINGLPGPLY
NCSSKDVFKLKVKPGKTYLLRLINAALNDELFFSIANHSLTVVEADAVYTKPFETDTLLIAPGQTTNLLL
KTKPTSPNATFLILARPYFTGAGTLDNTTTAGILEYKLPNTTHHTSIKALPLFRPSLPPINATKFVNVTQ
FVENLAKKFRSLATKQFPANVPQTVDRRFFFTIGLGTSPCPQNSTCQGPNGLKFAASVNNFSFALPSTAM
LQAHFSGQSNGVYTTDFPTKPLVAFNYTGTPPNITNVSNATRAVVLPFNTSVELVLQDTSIVGAESHPLH
LHGFNFFIVGLGFGNYDPTKDPANFNVVDPAERNTAGVPAGGWLAIRFFADNPGVWFMHCHLDIHTSWGL
RMAWIVQDGPQPNQKLPPPPSDLPKC

>XP_021815052.1
MANLLNSFTIVLSFLFIYGYNYNVHVMAQWPSGGSTRFYDFKVQTKRVTKLCSTKDMVTINGKFPGPSVY
AQEDDRIIVNVTNETPFNVTIHWHGVRQKLSCWFDGPSYITQCPIQAGQSFTYEFTMVKQKGTFFWHAHV
SWLRATVYGALIVYPKTGAPYPFKAPYEEHTLILGEYWLQDVVQLERATAASGGPPTPTNAYTINGHPGP
NYNCSNNDVFQIDVVSGKTYLLRLIHAGLNMENFFAIANHKLTIVEADAEYTKPFTTDRVMLGPGQTMSV
LVTADQSPGKYSMAMGPYMSAKGVKFQNISSIAYFQYSGAVPNSLSLPAKLPCFNDNLAVKTVMDGLRSL
NPVNVSRQIDTDLFVTIGLNVQKCHSKTPKQNCQGLNNGVMAASMNNISFVKPKISLLEAYYKKINGSFT

EDFPGVPLKFYDFVNGAPNNVPNNTQALNGTKALVLEYGARVQLIMQDTGTVTTENHPIHLHGYSFYVVG
YGTGNYNPKTANFNLVDPPYMNTIGVPVGGWAAIRFVADNPGVWFMHCHLDIHQSWGLGTVLVVKNGKGD
LEILPHPPADLPQC

>XP_021834606.1
MARFAFVLACALALLASSLASGAIVEHSFNVNNLTVTRLCKEQSITVVNGSYPGPTIRARDGDTLVVHVF
NQSPYNISIHWHGIFQLLSAWADGPAYVTQCPILPGNNYTYRFNITGQEGTLWWHAHVSWLRATVHGALI
IHPKAGRSFPFPKPYKEIPIILGEWWNGNVVDIENEGLATGIAPNNSNAYSINGLPGDLYDCSQNQTYQV
KVVKGKTYLLRIINAALNNQLFYKIANHNMTVVAIDAAYTTPYITDVVVVAPGQTTDVLITANQQTGSYY
MAATPYMSANINFDNTTTRGIIVYENSTSSSPIMPALPNPSDTPTAHKFNTNITGLAGGPQWVPVPTNVD
EHMFVTVGVNLELCPVNATCQGPFNNRLSASMSNESFQLPTNLSMMQAQFYNVSGIYTTDFPDQPLVKFD
YTDSNISLDLSLIYAPKSTKVKTLKFNSTVEIVLQNTAFLAIENHPIHLHGFNFHVLAQGFGNYDPINDP
KKFNLVNPQIRNTIGVPVGGWAVIRFTANNPGMWFMHCHLDVHLPWGLGMVFEVENGPTPWVLPAPPADL
PQC

>XP_021809566.1
MKILISLQLLGLTLLCVGIFHCQAWPRYTFVVEETPYRRLCSTKNILTVNGQFPGPTLYARTGDTIIVDV
YNKGNRNITIHWHGAKQPRNPWSDGPDYITQCPIQPGGRFTQTIIFSSEEGTLWWHAHSEWDRATVHGAI
VIYPKKGATYPFPKPHAEFPIILGEWWKEDIGQLYNETIQSGGDPNISNAFLINGQPGDLYPCSKPDTVK
LMVDYGKTYLLRLINSAVQEILFFSIANHKVTVVGSDASYTKPFSSDYVTISPGQTIDLLFTADQSPNHY
YIAAKAYVGGAGIVYDNTTTTAILQYNGNYTSTSTPSFPNLPSHNDTKASVHFTGSLRSLADQNHPVDVP
REITTSLFYTLSVNTLPCLKNSCAGPNGTRLAASVNNISFVDPSIDILQAYYYHVNGAFGTRFPNFPPFL
FNFTAQDLPLYLQTPKQGTEVKILEYNATVEIVFQGTNLVAGDDHPMHLHGFSF

>XP_021814580.1
MESWVRFLVLFLACLLPGLVESRIRHYKFNVVLKNTTRLCSTKPIVTINGRFPGPTLYAREDDTVLVKVT
NHVKYNVSVHWHGVRQLQTGWADGPAYITQCPIPPGQSYVYNFTITGQRGTLLWHAHILWLRATVHGALV
ILPKRGVPYPFPAPHKEVNVILAEWWKSDTESVINQAMSSGLAPNVSDAHTINGHPGPVANCSSQGGFTL
PVESGKTYLLRIINAALNEELFFKIAGHKLTIVEVDATYVKPFKTDTIVIAPGQTTNALITANQKSGKYL
VAASPFMDSPIAVDNLTATATLHYSGTLATTSTTLTNPPPQNATQVANNFINSLKSLNSKKFPAKVPLKI
DHNLLFTVGLGINPCPTCKAGNGSRVVASVNNVTFVMPTTALLQAHVFNISGVFTTDFPGNPPNTFNFSG
GPPANASMATTNGTKLYRLAYNSTVQLVLQDTGTIAPENHPVHLHGYNFYSVGRGVGNYNPKKDPKKFNL
VDPVERNTVGVPSGGWAAIRFQADNPGVWFLHCHLEVHTTWGLKMAFLVDNGKGPNESVLPPPSDLPKC

>XP_021816142.1
MLAMKILISVQLLGLTLLCVGIFHCQAWPRYTFVVEETPYRRLCSTKNILTVNGQFPGPTLYARMGDTII
VDVYNKGNRNITIHWHGVKQPRNPWSDGPDYITQCPIQPGGSFTQTIIFSSEEGTLWWHAHSEWDRATVH
GAIVIYPNKGATYPFPKPHAEFPIILGEWWKEDIGQLYNETIQNGGDPNISNAFLINGQPGDLYPCSKPD
TFKLMVDYGKTYMLRLINSAVQEILFFSIANHKVTVVGSDASYTKPFSSDYVTISPGQTIDLLFTADQTP
NHYYIAAKAYVGGAGIVYDNTTTTAILQYNGNYTSTSTPSFPNLPSHNDTKASVHFTGSLRSLADQNHPV
DVPREITTSLFYTLSVNTLPCLKNSCAGPNGTRLAASVNNISFVDPSIDILQAYYYHVNGAFGTRFPNFP
PLLFNFTAQDLPLYLQTPKQGTEVKILKYNATVEIVFQGTNLVAGDDHPMHLHGFSFYVVGWGLGNFDED
KDPLTYNLVDPPLQNTIAVPVNGWTTIRFKADNPGVWFMHCHLDRHMSWGMDMTFIVKNGKGLGAKILPP
PPGMPPC

>XP_021819117.1
MEFKKMSWVFAFAGFMFLGGICKANGHTHYYDFVLTESNFTRLCETKSILTVNGTLPGPTITVRKGDTAY
VNVHNQGSYGLTLHWHGVKQPRNPWYDGPENITQCPIQAGSNFTYWIIFSTEEGTLWWHAHSDWTRATVY
GAIIILPALNTTYPFTTPDAEETLVLGSWYKGDVNEIIENALATGGDPNVSDAFTINGEPGDLFKCSNAT
TYRWVVNYGKTYLLRLINAVLNEEMFFAIANHNLTVVAQDGAYLKPITTSYLMITPGQTMDILVVANQSP
SSYHVASGPFVDGDVAFNKNGNTTAILQYNGSTTPSTIPTPTLPNPTDGTAASNFTMQVRALASKDYPIS
VPLNITHTLFISVSVNERICPNSSCDGPNDNALAASLNNISFVTPSIDILQAYYGKIYGVYSANFPHKPY
IFNFTGHVRNDTIYPNFGTKVRMIEYGEEVEIIYQGTNMIAAENHPMHLHGFSFYLVGTGTGNFDPNQAP
KTYNLVDPPEVNTIGVPKNGWATVRFKADNPGVWFMHCHLERHASWGMATVLIVTNGNTNETSMLPAPAY
MPPCT

>XP_021819123.1

MGFYNKMSWVLAFLGFMFLGGLSNANGAIHHYDFVLKETNFTRLCSTKSILTVNGSLPGPTITVQKGDTA
FVNVHNQGLYGLTIHWHGVKQPRNPWSDGPENVTQCPIPAGTNFTYEVIFSSEEGTLWWHAHSDWTRATV
YGAIVILPALNTTYPFATPDAQETLILGSWFKGDVMELIEYALASGGDPNISDAFVINGQPGDLYDCSNE
TTYRWLVDYGKTYLLRVINAVMNEEQFFAIANHTLTVVAQDAAYIKPITTSYIMITPGQTMDILVTANQP
PSHYYIASKAYVDGTVAFNNSTTTAIVQYNGNYSAPSTTPFPTLPDYDNETAADNFTSQIRALASKEHPI
SVPLEIDSKIIISIAINERICPNSSCSGPDGNALSASLNNISFELPSVDILQAYYRGNNNGVYSTSFPNK
PPYTFNFTGDVGNNTIYPSFGTRVRMIKYGEGVEIVFQGTNIIAPENHPMHLHGYSFYVVGTGSGNFNST
TSPKTYNLVDPPEVNTIGVPKNGWAAIRFVADNPGVWFMHCHLERHSSWGMDTVLIVRNGNTTERKIRSP
PAYMPPCSKS

>XP_020426263.1
FFKKVKEATYTRLCSTKKILTVNGKFPGPVLQAHKGDTIYVNVHNKGSHNITIHWHGVKQPRNPWSDGPE
YITQCPIQPGDQFKQTIIFSNEEGTIWWHAHNDWARATVHGAIFVYPKRGAGYPFPKPHEQVQIILGQWW
RRDVREVLEEFIRTGGPPNVSDAHTINGQPGDLYPCSKSETFKLLVDQNKTYLLRIVNAAMNTIFFYSIA
NHNLTVVGVDGSYTKPVTTDYMTISPGQTVDAVLITNQQVGQYYMAARAYSSTLLIPFDNTTTTAIVEYK
KIGNNFTPFSSTPPLPHLPYYNDTNAAFTFYDSLKSLANEHHPIDVPIKITNRLVFAVSINTFPCSNNSC
AESNGTRLAASMNNISFVSPKTIDILEAYYYHIHGIYKKGFPDFPPLVFNFTGENLPLFLRVPKRGTKVR
VFKYGSIVELVFQGTNLGAPIDHPIHLHGFSFYIVGWGFGNFDKDKDPLNYNLIDPPLRNTVAVPISGWA
AIRFHANNPGVWFLHCHLEHHLTWGMNTVFIVKDGKNKKERLLPPPPRMPPC

>XP_007200191.1
MLSGKRNSALQILSLLLVFLHCQASSHNYHFVVKEATYTRLCSTKEILTVNGKFPGPVLQAHKGDTIYVN
VHNNGRYNITIHWHGVKQPRYPWSDGPEYITQCPIQPGHKFKQNIIFSEEEGTIWWHAHSDWSRATVYGA
IIVYPKRGASYPFPQPHEEVPIILGQWWKRDIMEVFEEFVQTGGEPNVSDAHTINGQPGDLYPCSKSETF
KLFVDENKAYLLRIINAAMNSILFFSIANHNLTVVGADGSYTKPVTRDYITISPGQTLDALLLTNQQVGQ
YYMAARAYSSSPAVAFDNTTTTAIVQYNNRNSTPFSSPPILPYLPYYNDTNAAFNFFDSLRSLANEDHPI
DVPKNITTRLISTVSVNTFPCPNSSCEGPNGTRLAASMNNISFVDPTTIDILEAYYYHINGVFREGFPDF
PPLVYNFTGEDLPLILQTPKRGTKVKVFDYGSIVECVFQGTNLVAGIDHPMHLHGFSFYIVGRGFGNFDQ
DKDPLNYNLVDPPHRNTVIVPINGWTAIRFKTNNPGVWFLHCHLERHLTWGMNTVFIVKNGKHKKEKLLP
APQEMPPC

>XP_021829543.1
MARFAFVLACALALLASSLASGAIVEHSFNVNNLTVTRLCKEQPITVVNGSYPGPAIRARDGDTLVVHVF
NQSPYNITIHWHGIFQLLSAWADGPAYVTQCPILPGNNYTYRFNITGQEGTLWWHAHVSWLRATVHGALI
IHPKAGRSFPFPKPYKEIPIILGEWWNGNVVDIENEGLATGIAPNNSNAYSINGLPGDLYDCSQNQTYQV
KVVKGKTYLLRIINAALNNQLFYKIANHNMTVVAIDAAYTTPYITDVVVVAPGQTTDVLITANQQTGSYY
MAATPYMSANINFDNTTTRGIIVYENSTSSSPIMPALPNPSDTPTAHKFNTNITGLAGGPQWVPVPTNVD
EHMFVTVGVNLELCPVNATCQGPFNNRLSASMSNESFQLPTNLSMMQAQFYNVSGIYTTDFPDQPAVKFD
YTDSNISLDLSLIYAPKSTKVKTLKFNSTVEIVLQNTAFLAIENHPIHLHGFNFHVLAQGFGNYDPINDP
KKFNLVNPQIRNTIGVPVGGWAVIRFTANNPGMWFMHCHLDVHLPWGLGMVFEVENGPTPWVLPAPPADL
PQC

>XP_021833316.1
MEFKKMSWVLAFVGFMFLGGICKANGHTHYYDFVLTESNFTRLCETKSILTVNGTLPGPTITVRKGDTAY
VNVHNQGSYGLTLHWHGVKQPRNPWYDGPENVTQCPIQAGSNFTYWIIFSTEEGTLWWHAHSDWTRATVY
GAIIILPALNTTYPFTTPDAEETLVLGSWYKGDVNEIIENALATGGDPNVSDAFTINGEPGDLYKCSNAT
TYRWVVDYGKTYLLRLINAVLNEEMFFAIANHNLTVVAQDAAYIKPITTSYLMITPGQTMDILVVANQSP
SSYHAASAPFVDGDVAFNNSTTTAILQYNGSTTPSTIPTPTFPNPADETAASNFTTQVRALASKDYPISV
PLNITHTLFISVSVNERICPNSSCDGPDGNALAASLNNISFVTPSIDILQAYSGAIYGVYSANFPHKPYI
FNFTGHVRNDTIYPNFGTKVRTIEYGEEVEIIYQGTNMIAAENHPMHLHGFSFYLVGTGSGNFDPNQAYK
TYNLVDPPEVNTIGVPKNGWATVRFKADNPGVWFMHCHLERHASWGMATVLIVTNGNTNETSMLPAPAYM
PPCT

>XP_021820658.1
MEMVPSIRALLLVACLLFPASVECMVRHYKFNVVQKKTSRLCSSKPIVTVNGRYPGPTIYAREDDTVLIK
VVNHVKYNVSIHWHGVRQLRTGWADGPAYITQCPIQPGQNYVYNFTITGQRGTLWWHAHILWLRATVHGA
LVILPKLGVPYPFPAPQKEVVVILGEWWKSDVESVINQALKSGSAPNVSDAHTINGHPGPLSTCSSQGGF

KLPVRPGKTYMLRIINAALNEELFFKIASHKLTVVEVDAVYTKPFKTDTVLIAPGQTTNVLLSTNHGTGK
YLVAASPFMDNPNILVDNKTGTATLHYTGTLESTRTTLTAPPPQNATLVATKFTNSLRSLNSVKFPARVP
LKIDHSLLFTVGLGLHTCASCANGNRLVANINNVTFVMPKISLLQAHFFNISGVFTDDFPGNPLNPYNYT
GPQPTNMQTMKGTRLYRLAYNSTVQLVLQDTGMIAPENHPVHLHGFNFFEVGRGLGNFNPKKDPKRFNLI
DPVERNTIGVPSGGWTAIRFRADNPGVWFMHCHLEVHTTWGLKMAFVVDNGKGPNESVLPPPSDLPKC

>XP_021833693.1
MASSFWNRFFCGLVFLCFGFLGLITSPAEAAIKNYQFDKRKSCTLCMAFHLITSSVFSQVQVKNVSRLCH
AKSIVTVNGRFPGPTIYVREGDRVVINVTNHAQYNMSIHWHGVKQYRNGWADGPAYITQCPIQTGSSYTY
DFNVTGQRGTLWWHAHILWLRATVHGAIVIMPKQGTPFPFPQPHREAEIILGEWWNVDVEEFVKKANNLG
LAPNTSDAHTINGKPGPLFPCSEKHTFVMEVEQGKTYLLRIINAALNDELFFAIAGHNLTVVEVDAVYTK
PFTTQAILIAPGQTTNVLVQANQASGRYFMATRPFMDAPVPIDNKAATGIFQYRGIPNTVLPSLPQLPAS
NDTSFALSYGKKLKSLNTPNFPANVPLKVDRKLFYTIGFGKEPCPTCLNGTRFVASLNNISFEMPQVGLL
QAHYFNLKGVFKTDFPDRPQTPFNYTGAPLTANLGTSTGTRLSKIAFNSTVELVLQDTNLLTVETHPFHL
HGYNFFVVGTGIGNFDPAKDPAKYNLVDPVERNTVGVPTGGWTAIRFRADNPGVWFMHCHLELHTGWGLK
TAFVVEDGPGSDQSVLPVPKDLPPC

>FVgene05430
MNDSFPGPVIRVQKGDTVYVNVHNPGDYGMEYINQEILGQMVRSISQCPIEPNTNFTYEVIFSDEEGTLWWHAHSEW
TRASVHGAIVVKPLEETGFPFLEPDGEEIIIFGSWYLIERLNEVVAEKIKEGHDTPRSDCYTINGQPGDFGWCSNDS
THRWQVEYNKTYLLRIINAVMNAELYFAIAEHTLTVVGMDGSYVKPIVTDFIMISTGQTMDILVTANQPLGRYYMAG
RQFDTAIDAFQAYDKTNVTAILEYKGNYSDLVYPIFPTTLPAFEEILPAVNFTSRLKSLASIDHPTNVPNNITTIMY
ITVEMNNGTFINGGEEIKSGLVSILNNLSWVNPSTDVLLDYYRNMSGFYTADFPDYPPSMFDFIADDQPPDRTIATV
KATSVKVLEYNEEVEVVSQGTNVLDGSEDHPMHLHGYSFYVVGSGFGNFDNETDPKGYNLIDPPKLTTVSLPKKGWV
AIRFKASNPGPGSGYGTVIWIDISVGV

>FVgene07465
MEAPSSILLLGLCLLFASSAMTSTLAEPKAHHHEFVVQATPVKRLCKTHNSITVNGQFPGPTLEVNNGDTLVVKVTN
KGRYNVTIHWHGIRQMRTGWADGPEFVTQCPIRPGGSYTYRFTVQGQEGTLWWHAHSSWLRATVYGAIIIHPKQGDS
YPFPKPNRQETLLLGEWWDANPIDVVRQATRTGAAPNVSNAYTINGQPGDLYNCSSKGTVIVPIDSGETNLLRVVNA
ALNQPLFFSVANHKLTVVGADASYTKPFTTKVLMLGPGQTTDVLISGDQQPSRYYMAAHAYFSAQNAPFDNTTTTAI
LEYKSVRCNNTTSCKKSKPIMPQLPAFNDTNTAEVFTRSFRSPRKVEVPTEIDENLFFTIGLGLNKCPKHFRSRRCQ
GPNGTRFAASMNNVSFVLPNNMSILQAYQQNIPGVYTSDFPANPPKKFDYTGNVSRSLWQPSFGTKGYKLKYGSRVQ
VVLQDTSIVTPENHPIHLHGYDFYILAQGFGNFNAKTDTKKFNLVDPPLRNTVSVPANGWAVIRFVADNPGAWLLHC
HLDVHINWGLGMFFLVDNGVGELQTIEPPPADLPRC

>FVgene07472
MEAPSSILLLGLCLLFASSAMSTLAEPKAHHHEFVIQATPAKRLCKTHNSITVNGQFPGPTLEVNNGDTLVVKVTNK
ARYNVTIHWHGIRQMRTGWADGPEFVTQCPIRPGGSYTYRFTVQGQEGTLWWHAHSSWLRATVYGALIIHPKQGDSY
PFPKPNRQETLLLGEWWDANPIDVVRQATRTGAAPNISDAYTINGQPGDLYNCSKKGTVVVPIDSGETNLLRVVNAA
LNQPLFFSVANHKLTVVGADASYTKPFTTKVLMLGPGQTTDVLISGDQQPSLYYMAAHAYFSAQNAPFDNTTTTAIL
EYKSVRCNNTTSCKKSKPIMPQLPAFNDTNTADVFTRSFRSPRKVEVPTEIDENLFFTIGLGLNKCPKHFRSRRCQG
PNGTRFSASMNNVSFVLPNNMSILQAYQQNIPGVYTSDFPANPPKKFDYTGNISRSLWQPSFGTKGYKLRYGSKVQV
VLQDTSIVTPENHPVHLHGYDFYILAQGFGNFNAKTDTKKFNLVDPPLRNTVSVPANGWAVIRFVADNPGAWLMHCH
LDVHINWGLGMVFLVDNGVGQLQTIEPPPADLPRC

>FVgene07983
MEMVLWFRALLLVACISCLFPAPAECLERHYKFNVVLKKTSRLCSSKPIVTINGKYPGPTIYAREDDTVLVKVVNHV
KYNVSIHWHGIRQLRNGWADGPAYITQCPIQPGQQYVYNFTVTGQRGTLWWHAHILWLRATVYGALVVLPKLGVPYP
FPAPKKEVVVILGEWWKSDVEAVINEALKSGAAPNVSDAHTINGHPGPLSLCSSQGGYKLPVQPGHTYLLRIINAAL
NDELFFKVANHKLTVVEVDAVYTKPFETTTILIAPGQTTNVLLTADRSTGKYLVAASPFMDNPTVAVDNKTATATLH
YRGSLASTNTILTVPPPQNATRLATSFTKSLRSLNSDTYPAKVPLKIDHSLMFTVSLGLQTCPSCANGNRVVADLNN
VTFVMPKISLLQAHVFNISGVFTDDFPGNPLMPYNYTGAQQPTNMQTMKGTRLYRLAYNSTVQLVLQDTGMIAPENH
PVHLHGFNFFEVGRGLGNFNSKTDPKIFNLIDPVERNTVGVPSGGWTAIRFRADNPGVWFMHCHLEVHTTWGLKMAF
VVDNGKGPNESVLPPPSDLPKC

>FVgene09404
MKAFCQLVLLVTLLIISGVLPSCVSQTTRRFEFNVEWKNVTRLCHKKPLLTVNGEYPGPTIAVHEGDNVEIKVTNRI
AQNTTIHWHGIRQIRTGWADGPAYITQCPIREGKTYTYKFTVIDQRGTLWWHAHHSWQRASVYGAFIIHPRTPYPFS
VPIQDEFPIIFGEWWNGDIDSVESEMMKYGGGPAISNAYTINGLPGPFYNCSNKDTLIKTVEHGKTYMLRIINAALN
DELFFAVANHTLTVVEIDAVYTKPFKTKAIMIAPGQTTNVLLTANQVPDSSGMFLMVAWTYLTSVFPFNNSTTAGFL
QYKNLMSTEHTSQHPNKVTTFDPVMYNFPQMEDTKYYTKFSDNLRSLHSPRYPCNVPKTIHKRIVAVISLNLQDCPA
NKTCKGYANKRFFASMNNQSFVRPALSILESHYKKLKTSQYSTDFPEKPRKPFDYTGVDPLTENMNTEFGTKIMQVP
YGTNLEIVLQGTSFLNVENHPIHVHGHNFFIVGKGFGNFDVSKDPAKYNLVDPPERNTVAVPSGGWAAIRIKADNPG
VWFIHCHLEEHTSWGLASGFIVQNGHGPSESLLPPPKDLPSSKPVPTLPSFYLTFICSNTPHTSPPSLLLQPSSFHT
LTHPPLPLTHIAENPKSKLNKPTMSPNPPPLFPFLCLLFTLLPSSLALTNPDPAAVQPFLPKSSPPATIPAFPEQSD
IAGCPLQLPDELFHGVKAACSAKKGDPAGHLQHSRCCPVLAAWLYSAYSATALGRSGRVGPAVTGHGSNTTTAYDPP
LLPDDSETCVQDLGKALSAKGIELMRPNETCDMVYCNCGIRLHPLSCPEAFSVNQNGKLIGDKSVKKLEKNCLSSSN
NVNNFPGLGGCSKCLNSLKLLNKKKTSNSSKLEDRTTKMHNKDCQLMGLTWLLAKNRTAYMHTVTSVFRAIMVSNDG
SDPQSCTLNSDGMPLAVDSSEISDQSPSNILAVSFTFCVVSLSLLYVQLTMS

>FVgene11905
MQPRNPWSDGPEYITQCPIQPGTNFTYEVILSKEEGTVWWHAHSDWTRASVHGAIVILPAVGTTFPFPKPDEDEVIV
IASWYKGDLQARVDEAMDNDENLPHSDAYTINGQPGDRSLSLLQWYDN

>FVgene11906
MDVLLEANQVLWRYYMAARHFSSENVAVGDFDHANVTAILEYNGNYTHLTSPVFPSSLPMYYDFAAYRNFTNQIRSL
ATPEYAVHVPLDNEITTRMFIRTSMNSLFCEDSSSGCERNKSVQAFYVVGYGFGIYDNATDPKGFNLIDPPEVTTFG
VPKNGWLAIRFTANNPGVWFWHCHMDRHLTWGMEAAFIVKNGDTNETSIRKPPTDMPLCTIPLHSGLKDFPEIITTK
KGDYL

>FVgene11910
MNIISLVVDSHEAIISPTCGLIALHGIRQLRNPWSDGPEYITQCPIQPGTNFTYEVILSKEEGTVWWHAHSDWTRAS
VHGAIVILPAVGTTFPFPKPDEDEVIVIASWYKGDLQARVDEAMENDENLPHSDAYTINGQPGDLCPCSNEATYRRK
VDYGKTYLLRIVNANIDADIFFAIAQHNFTVVGLDGAYIKPIDTTYILLTPGQTMDVLLEANQVLGHYYMAGRHFSS
ENVAVVNFDHANVTAILQYNGNYSHLTSPVFPSSLPMYYDFVASLKFINQIRSLAIPDYVINVPLDYEITTRMFITT
SMNSLYCEDSSSGCETKQISASVNNISWVNPSTDILEAYYRNISGVYSADFPDNPPSFYNFTDESYSIDTVLTVQGR
KVKVLNYNESVEIVFQGTNVLGGSVNHPMHLHGYSFYVVGYGFGIYDNVTDPKGFNLVDPPEVNTFAVPKNGWLAIR
FSANNPGVWFWHCHMERHMTWGMEAAFIVKNGNTVETSIRQPPDYMPPCNIPLLDSTLKNLQEITKKKVDYR

>FVgene11940
MQPRNPWSDGPEYITQCPIQPGTNFTYEVILSKEEGTVWWHAHSDWTRASVHGAIVILPAVGTTIPFPKPDEDEVIV
IASWYKGDLQARVDEAMENDENLPHSDAYTINGQPGDLCPCSNVNANIDADIFFAIAQHNFTVVGLDGAYIKPIDTT
YILLSPGQTMDVQVLGSYYMAARHFSSENVAVGDFDHANVTAILEYNGNYTHLTSPVFPSSLPMYYDFAAYQNFTNR
IRSLATPEYAIQAQVVKRNKSVQANISGVYSADFPDYPPSFYNFTEESYSIDTVLTVQGRKVKVLNYNESVEIVFQG
TNVLDGSVNHPMHLHGYSFYVVGYGFGIYDNATDPKGFNLIDPPEVTTFGVPKNGWLAIRFTANNSGVWFWHCYMDR
HLTWGMEAAFIVKNGDTNETSIRKPPTDMPLCTIPLHSGLKDFPEIITTKKGDYL

>FVgene12086
MRLIREEIDIPELESETSESHLEVSYPASSSYPRKNNMKPQKNCSILSTEILGFLFLLVLFISSVAGEVHFYDFIVR
EKNFTKLCLTKSILVVNDGFPGPEIRVHKGDTVYVNVHNQGDYGLTIHWHGIKQPRNPWSDGPEYITQCPIPPGTNF
TYEVILSTEEGTLWWHAHSEWTRATVHGAIVILPSAGTTYPFPQPDEDETIIIASWYEGDIKELYDEAMDDGSDLPH
SDAYTINGEPGDFCACSKETTYRRTVDYGKTYLLRIVNANMNAEHFFAVAEHNLTVVGLDGSYVKPIETSCIVISAG
QVMDVLLKANQSPGQYYMAARQYSSEDAEVTGFDHVNVTAILEYRGNYTYQTSPSFPTSLPMYLESPAAISFTNRIR
SLATPEYPIDVPNISGVYTTDFPDQPPTFYNFTEETFVDDIVFTVQGTKVKMLNYNESVQIVFQGTNVMKGSVNHPM
HMHGYSFYVVGFGYGNYDDEADPKGFNFVDPPKVTTFAIPKTGWLAIRFTASNPGVWFWHCHLDRHLSWGMDTAFIV
KNGSTTETSIRPPPLYMPPCEPHLNSRIKNSHEIIL

>FVgene14031
MGFSILSLQAFFRLFLLSVLTLCLSSELAAASITKHYKFEIKLQNVTRLCHTKSIVTVNGQFPGPRIVAREGDNLLI
KVVNHVPNNISIHWHGIRQLRSGWADGPAYVTQCPIQTGNSYVYNYTIVGQRGTLWWHAHLSWLRSTVYGPLIILPK
RGAPYPFIKPYKEVPIIFGEWWNADPEAVINQALQTGGGPNVSDAYTINGLPGPLYNCSAKDTFKLKVQAGKTYLLR
LINGALNDELFFSIANHTLKVVETDAVYVKPFKTNTILITPGQTTNVLLKTKPNFPNATFLMTARPYVTGLGTFDNS

TVAGILEYEATTLHSTLPMKKLPLHKPILPALNDTSFVTKFVSKLRSLNTPQFPANVPQKVDKHLFFTVGLGSNPCS
QQQNSNQTCQGPNGTMFAASINNVSFTMPTTALLQSHFSGQSKGVYSSDFPISPLTPFNYTGPPLNNTNVGNGTKLM
VLPFNTSVELVLQGTSILGAESHPLHLHGFNFFVVGQGFGNFDQNKDPKKFNLVDPVERNTVGVPSGGWVAIRFLAD
NPGPYELGFEDGMGSARWKASQPEAASSTENCTYAVTIETTCIKGASTSNNISLRFGDTNSNDVLVKHLNSKHIRRV
DPVEPQVLDEVPRKPFQACVVDQFQVMGQCVDSPICYLYLKLAGTDDWRPGFAQVHVLEGSHSHLSSDYFYFRRYLP
RRAWHGSDTCQKEENSGMHPEGAAPRGCCTQRVLHPAAPLLSVYCLPGFCIKTIFLFLFASPKSRVSLRGPKAERFR
CFISAIGVLRNSRKYGPESPVFDLDPEIEIQCIEGAFQKSATGLSSMDEAMVLSGVDRNGIKFNLPPTKEVQVVDSV
VLEGAQGAPPSSTELN

>FVgene14033
MSLAVAANSKLFREYIGAEGVGITFSDVPIDPGVEFHFLLSFGIDYTTSSRPKPTNGIFNAFWDTDNLSPSHVSAIK
ARHRNVKVGLSLGGDTVAGVSANFTPISINSWVRNAISSIVKITKEYKLDGIDIDYEHFNTDPNTFAECIGRLFFYL
KQNKVVSFTSIAPYDDDAVQPYYLALWRKYGHLIDYVNFQFYAYDKGTTVTQFMKYFGTQSSNYKGGKVLVSFGTDQ
SGGLSPEHGFFDACATLKSQGKLNELDSMQIVVGMVVLIKPGNMQVTIIISNSVFRVRRVTWRRGDTWQRLDLRGKA
GRLFVLCCITLGLIPQLALASITRHYTFDVQLQNVKRLCHTKSIVTINGQFPGPRIVAREGDRLLIKVVNHVQSNIS
IHWHGIRQLQTGWADGPAYITQCPIQTGNSYVYNFTIVGQRGTLWWHAHISWLRSTIYGPLIIIPKRGVPYPFTKPY
KEVPIIFGEWWNADTEAVIKQALQTGGGPNVSDAYTINGLPGPLYNCSAKDTFKLKVQAGKTYMLRIINAALNDELF
FSIANHTLKVVEADAVYVKPFDTNTILIAPGQTINVLLKTKPSHPNATFFMTARPYVTGLGTFDNSTVAGILEYEST
ALHSTLPMKKLPLHKPILPVLNDTSFATKFSNKLRSLNTPQFPANVPQKVDKRLFFTIGLGTNPCSQQKHNNQTCQG
PNVTMFAASINNISFTMPNTALLQSHFFGQSNGVYSPYFPISPLIPFNYTGAQLNNTNVSNGTKLMVLPFNTSVELV
LQDTSILGAESHPLHLHGFNFFVVGQGFGNYDQNKDPKKFNLIDPVERNTVGVPSGGWVAIRFLADNPGVWFMHCHL
EIHTSWGLRMAWLVLDGKLPNQKLLPPPLDLPKC

>FVgene14746
MASSSSWNQIFCGLAFVFFGLFLGSSTSPAEAAIKKYQFDIQVKNVSRLCHAKPIVTVNGRFPGPTIYVREGDSVLV
NVTNHAQYNMSIHWHGLKQYRNGWADGPAYVTQCPIQTGNSYTYAFNVTGQRGTLWWHAHILWLRATVYGAIVIMPK
QGTPFPFPQPHREAEIVLGEWWNADVEEVVKQGTNMGLPPNSSDAHTINGKPGPLFPCSEKHTYAMEVEQGKTYLLR
IINAALNDELFFGIAGHNLTVVEVDAVYTKPFTTQAILIAPGQTTNVLVQANQVPNRYFMAARPFMDAPLQIDNKTV
TAILQYRGVPNSVLPILPQLPSPNDTTFALGYDKKLKSLNTPKFPANVPLKVDRHLFYTIGFGTQSCPSCLNGTRFV
ASLNNITFEMPQVALLQAHYFNIKGVFKTNFPDKPPTPFNYTGAPLTANLGTSKGTRLSKIAFNSTVELVLQDTNLL
TVESHPFHLHGYNFFVVGTGIGNFDPAKDPAKYNLVDPVERNTVGVPTGGWTAIRFRADNPGVWFMHCHLELHTGWG
LKTAFVVEDGPGKHQSVLPPPKDLPKC

>FVgene15091
MLVVNESYPGPPIRARKGDTIYVNVYNEGFYGFTIHWHGVKQPRNPWFDGPEYVTQCPIAPGTNFTYQVQLTEEEGT
VWWHAHSDWTRASVHGAIVVLPALGTMFPFPEPDEEEIIVFGSWYLGNLRERVEESLQPGSTETLPQSDAYTINGQP
GDFLPCSINATWRRKVDHGKTYLLRLVNANIDAEFFFAIAEHNLTVVGLDGAYTKPINTRYIVVTPGQTMDVLLYAN
STPGLYYMAGRQYLSTTSDTGFDHMNATAILQYNGNYTTTDAPLFPEHLPMYMSQAEAFNFTNKIRSLASPEHPVNV
PKESEITTRMFITASMNSLYCAPDLGPTCVNISFATSVNNISWVNPTTDSILQAYYRNISNPIYETDFPDQPPTYFN
FTDGGTTMSTVLTVQGTKVKVLDYNETVEIVFQGTNVMGGAVNHPMHLHGHSFYVLGFGFGPFDPVKDPKGYNLVDP
PYVATFVTPKNGWLTIRFVADNPGVWFWHCHVDRHMTWGMEAAFIIKDGGTAETSMLPPPAQMPSCDVPLDSAIRNY
EALIEKQMDYRNFTNDSLIQKVKVLDYYESVEIVF

>FVgene16063
MAARAYSSAFGAGFDKTMTSVIVKYRGSDEQDTHPRRLPIFPHLPPYNRTQAATDFTKQFRSLATKNHPTKVSIQSI
DILRAYYYKIPKVFEKNFPVKPPKEFNYIGDDLPNEFLTSSYGTKVMVLKYNASVELVLQGTNVLASDNHPVHLHGY
SFYVVGSGFGNFNPKEDPLRYNLVDPPKESTVGVPKNGWVAIRFRANNPGVTGEESCQGSTPVGQRRPWWWKGDALA
ASGLEDEEVWLFGLDICG

>FVgene16187
MANNPSSLMPSELIDRCIGSKIWVIMKGDKELVGTLRGFDVYYVNMKYLSDQVVELLSYSQRNIELVQCKTRQEKVDV
GERLILVIISPVSVVLKRRVCIRVFVLSNCFSSLEGCLGNIEHIKLVWRRQDKTMRILESVVNLLNNFTQHFMDSLL
CILRLRMSSLSDVDKTRWLLESMQPGVCIRFLAKSNCFSSVEGCVANVMRAVAIPANCWCDNEITAEGRRITKLDQI
LLNGNNIAIEVSRISIHLQLLGLLFAVNGFLLHCQAWPARYTFVVEDTPYTRLCSTKNILTVNGQFPGPTLYVHKGH
TISVEVINRGSRNITLHWHGVAQPRNPWSDGPDYITQCPIQPGAKFTQKIIFSTEEGTLWWHAHSEWDRATVHGAII
IYPSKKSKYPFPKPHAEIPIILGEWWKEDIGKLYTRTIQTGGDPNISDAYLINGQPGDLYPCSKSDTFKVTVDYGKT
YLLRLINSAVQEILFFAIANHKVTVVGTDASYTKPFTTDYVTISPGQTMDLLLHANQSPNHYYMAATAYVGGAVPYD

NTTTRALLQYTNRGTKGNYTPSSIPLLPNLPAHNDTNASVHFSGSLRSLADKNHPIDVPLKITNHLFFTVSVNTFAC
PNNSCAGPNGTRLAASVNNISFVNPSIDILQAYYYHVNGAFGTRFPKTPPLLFNFTAQDLPLYLQTPKRGTEVKVLE
YDATVELVFQGTNLVAGDDHPMHLHGFSFYVVGMGLGNFDKDKDPLTYNLVDPPLRNTIAVPVNGWTTIRFKADNPG
VWFMHCHLDRHMSWGMDMTFIVKNGKDPKAHILPPPPDMPPC

>FVgene17621
MHALDDVSGEFVGLIASSISHQPGQPQPVTRVQDAHANAQAEPHQQSTMFLVISLVRGEVHFYDFAREKNFTRLCET
KSMLVVNDIFPGPEIRVRKGDTVYINVHNQGSYGITIHWHGILQLRNPWSDGPEYITQCPIQPGTNFTYKVLLSTEE
GTVWWHALIVTGHELAFMVPLSSCLLLASWYSGDLKERVDEAMEEGTSLPRSDAYTINGQPGDLYACSKETTYRRKV
DYGKTYLLRIVNANIDSTLFFAVAQHNLTVVGLDGSYIKPIYTTYMLISPGQTMDVLLKADQVLGRYYMAGRHFSSD
NAKVSEIGPISVTSIMEYNANYTHPTSPLFPKTIPMYKDSAAALNFTCQIKSLASPKHPINVPLDNDITTRMFVTAS
MNSLYCEDSGFLSCVETPVATSVNNISWVNPRSTDLLQAYYRNISGVYSTDFPDQPPTFYNFTEETYSNDTMLTVQG
TKVKVLSYNESVEIVFQGTDVQGGSVNHPMHMHGYKFYVVGYGFGNYDNETDPKVFNLVDPPHVTTFGVPKNGWLAI
RFIANNPGVWFWHCHMERHLTLGMEAAFIVKNGDTAETSILEPPAYMPTCTFSIVINGSPGMFFKPSRGLRQGDPPY
LCLLISEKKPLSTPNMVRFSFEPKDRGKQRITTLDSWNSQKLRLSINWNSSLLIPSEAGVGVAVVGECVWGTVAPIS
MAFESVVLVPAMKRTSALKKKCGCPSVLLCPAPTTALLHPSGHQRHLRSLSLASEIDLKVWAATSTQNFRQGRREEK
VIGDTSGVCSGGGAPSLASPIKDSDEFVEKKIDQ

>FVgene18442
MGYLHVPAMAAWLFGLCVAAALMPELAEGKTRHYTFNIQYHNVTRLCSTRKIVTVNGKFPGPRLVAREGDRVLVKVV
NHIKENVTIHWHGIRQLRSGWADGPAYITQCPIQTGQSYTYNFTIIGQRGTLWYHAHISWLRSTLYGPIILLPRRNE
SYPFVKPYKEIPIILGEWFKTDPEAVINQALQTGGGPNVSDAYTLNGLPGPMYNCSSNDVFKLKVKPGKTYHLRLIN
AALNDELFFSVANHSLTVVCADATYVKPFTTDILLITPGQTTGVLLKTKPNFPNATFLILTRPYFTGAGTLDNTTAA
GILKYEHPLSSSGTPSSIKTLPLLKPSLPPINGTKFVNVTQFVQNLASKYRSLDSAKYPAKVPQTVDRKFFFTVGLG
TSPCPKNTTCQGPNGQKFAAAINNVSFAMPSVAMLQTHFFAQSNVTYRTDFPSNPIVQFNYTGTPPNNTNVINSTRA
VVIPFNTTVELVLQDTSILGAESHPLHLHGFNFYIVGQGFGNFDQNKDPAKFNLVDPVERNTVGVPAGGWAAIRFLA
DNPGVWFMHCHLEIHTSWGLKMAWIVQDGPQPNQKLPPPPADLPQC

>FVgene18444
MGSSHPASPALSMATVFFSVCAFFCVLPELAVGKHAGVTRHYKFNIMSQSVTRLCKTKSIITVNGQFPGPRIVAREG
DRLVIKFSPPMLSIGLHFDDRHGIRQLRSGWADGPSYITQCPIQTGQSYVYNFTITGQRGTLWWHAHISWLRATLYG
PIVILPKKHVPYPFPQPFKEVPIIFGEWWKADTETVINQAIQTGSGPNVSDAYTINGLPGPLYNCSAKDTYKLKVKP
GKTYLLRLINAALNNELFFSIANHTFTVVEADAVYVKPFSTKVVLITPGQTTNVILRTKPKSPNATFVMAARPYVSG
PAAFDNTTTSGLLEYETSVSSNHNKNKNKRKKKLLLLTPVLPKLNDTMFSMNYNKKIRSLANPKFPSANVPKTVDKN
FFFTVGLGLSPCSKNQNCPGPNNTRVAAAINNVSFVQPSTALLQAHFFNKTKGVYTTDFPANPLVKFNYTGTPPSNI
AVSTGTKVVVLPFNTRVELVLQDTSIIGAESHPLHLHGFNFFVVGFGFGNFDPKNDPKKYNLVDPAERNTIGVPSGG
WVALRFLADNPGVWFMHCHLEVHTSWGLKMAWVVMDGKGRHQKLPPPPSDLPKC

>FVgene18694
MENNNCMSVLSCVSTNVASAFFASLERCSCINLSTTDFEDDNNFSAADDHGSLLLSSGPTSEPNLNQPAPASAAAAA
AQEVLCGLVSCRHMGKKGTVDICLHMALPESHIDDELDVILVFLRISITRQRWIIYSFALFPFRTRSLGTAALRLLR
EASAELDEPFPTVESEFRKEQNLLLILGYNVLVMAQWPSGGQTRFYDFKVQSTRVAKLCTSKDMVTINGMFPGPVVY
AQEDDRVIVKVTNETPYNTTIHWHGVRQKLSCWFDGPSYITQCPIQAGQSFTYEFTMVKQKGTFFWHAHVSWLRATV
YGALIVYPKTGVPYPFKVPYEEHIIILGEYWLQDVVQLEHDTAASGGPPTPASAFTINGHPGPNYNCSNNDVYQIDV
VSGKTYMLRLVHAGLNVESFFAIANHNLTIVEADAEYTKPFTTDRVMLGPGQTMVVLVTADQPQGKYSMAMGPYMSA
KGVKFQNISSVAYFQYLGAVPNSFSLPAKLPSFNDNLAVKTVMDGLRSLTAPVNVPRKIDSELFVTIGLNVQKCHSK
TPKKDCQGLNNGVMAASMNNISFVKPKVSVLEAYYKRINGYFTEDFPKVPQKFYDFVNGAPNSIPNNTEAISNGTRT
LVLEYGTRVQLILQDTGTVTTENHPIHLHGYSFYVVGYGTGNYSPQNASFNLVDPPYMNTIGVPVGGWAAIRFIADN
PGKVYVW

>FVgene18757
MARFVFALACALALLASSVASAAIVEQSFNVNNLTVTRLCQNQLITAVNGQYPGPTIRAREGDTLVVHVLNQSPYNL
TIHWHGIFQLLSAWADGPAYVTQCPILPGNSYTYRFNITGQEGTLWWHAHVSWLRATVYGALIIHPRSNRSYPFAAP
FQDVPIMLGEWNANVVDVENQGLATGGAPNNSDAYTINGLPGDFYNCSQNQTYKLNVVRGKTYMLRIINAALNNQL
FFGIAKHNMTVVAVDASYTKPYVTEVVLVAPGQTTDVLITANQPVGSYYMAATPYASAAIPFPNTTTRGVLLYNGYN
SSSAPLMPALPNPRDTSTAHRFSSNLTGLAGGPQWVPVPLQVDEHMFVTIGVNLDVCAEGMTCQGPLNNRLSASMNN
ESFQLPTTLSMLEASVYNVSGIYTTDFPDNPPVEFDYTNANISLDLNLIFAPKSTKVKKLKYNSTVEIVLQNTAFLA

IENHPIHLHGFNFHVLAQGFGNYDSVNDSKNFNLVDPQLRNTIGVPVGGWAVIRFQANNPGVWMMHCHLDVHLPWGL
AMAFEVENGSTPSSTLPPPPLDLPKC

>FVgene18760
MARFVFALACALALLASSVASAAIVEQSFNVNNLTVTRLCKNQLITAVNGQYPGPTIRATEGDTLVVHVLNQSPYNL
TIHWHGIFQLLSAWADGPAYVTQCPILPGKSYTYRFKITGQEGTLWWHAHVSWLRATVYGALIIHPRFPRSYPFPAP
FQDVPIMLGEWWNANVVDVENQGLATGGAPNNSNAYTINGLPGDFYNCSQNQIYKLNVVRGKTYMLRIINAALNDQL
FFGIAKHNMTVVAVDASYTKPYVTEVVVVAPGQTTDVLITANQPVGSYYMAATPYASATIPFPNTTTRGVLIYIGYL
SSRAPLMPALPNPRDTSTAHRFSSNLTGLAGGPHWVSVPLQVDEHMFVTIGVNLDVCAEGVTCQGPLNNRLSASMNN
ESFQLPTTLSMLEASVYNVSGIYTTDFPDNPPVKFDYTNANISLDLNLIFAPKSTKVKKLKYNSTVEIVLQNTAFLA
IENHPIHLHGFNFHVLAQGFGNYDSVNDSKKFNLVDPQLRNTIGVPVGGWAVIRFQANNPGVWMMHCHLDVHLPWGL
AMAFEVENGPTLSSTLPPPPLDLPKC

>FVgene18812
MASWIRVLLLLLACLLPALVESRVRNYKFNVVLRNTTRLCSTKPIVTINGRFPGPTLYAREDDTVLVKVTNHVTYNV
SIHWHGIRQIRTGWSDGPAYITQCPIPQGQSFTYNFTITGQRGTLLWHAHILWLRATVHGALVILPKRGVPYPFPTP
HKEVPVVLAEWWKSDTEAVINEALKGGLAPNVSDAHTINGHPGLVSPNCTTDGGFKLSVESGKTYLLRIINAALNEE
LFFKIAGHKLTIVEVDATYVKPFKTDTIVIAPGQTTNALITANQKSGKYLVTASPFLDTPGIAVDNLTSTATVHYSG
TLSNSPTTLTSPPPQNATQVANSFTDSLRSLNTMKFPAKVPLKIDHNLLFTIGLGINPCPSCKAGNGSRVVASINNV
TFVMPTTALLQAHFFKTKGVFTTDFPANPPNAFNFSGGLPANSSLATTNGTKVYRLAYNSTVQLVLQDTGIIAPENH
PVHLHGFNFFAIGRGLGNYNPKTDPKNFNLVDPVERNTIGVPSGGWVAIRFIADNPGVWFMHCHLEVHTSWGLKMAF
LVDNGKGPNQSLLPPPKDLPTC

>FVgene20428
MAKTTNLCFCSLLPFLCLVAFFFIPAKAAVKSYQFNIQVKNVSRLCHSKPIVTVNGMFPGPTIYAREGDTLLVNVTN
HAQYNMSIHWHGLKQYRNGWADGPAYITQCPIMTGNTYTYNMTITGQRGTLWWHAHIFWLRATVYGAIVILPKQGTG
FPFPQPYSETNLILGEWWNNDVEEVVKQGNRLGLPPNMSDAHTINGKPGPLFPCSEKHTYAMEVEQGKTYLLRIINA
ALNDELFFAIAGHNLTVVEIDAIYTKPFTSQAILIAPGQTTNVLVQANQVPSRYFMAARPFMDAPLSIDNKTATAIL
QYKGIPNSVLPVLSQLPALNDTAFALSYNAKLKSLNTAKYPANVPLKVDRQLFYTIGLGINQCSTCLNGTQLTASLN
NITFVMPKIGLLQAHYFNTKGVFTTDFPDRPPTPFNYTGAPLTANLGTKLGTRLSKIAFNSTVELVLQDTNLLTVES
HPFHLHGYNFFVVGTGVGNFDSKKNPAKFNLVDPPERNTVGVPTGGWVAVRFRADNPGVWFMHCHLELHTGWGLKTA
FVVENGKSSDQSVLPPPADLPPYVTRSDRRGKKTRFVLFGGEENALLLDWEKKMLQTGPRQKSMWASCPLVAVEVFQ
NEWARFRLGLSK

>FVgene20911
MKFSSRIQVILLVAFIYPALMVESLVRHYNFRVVLKSTTKLCSSKAILTVNGKFPGPTLYAREDDTVIIRVTNHGNR
NVTIHWHGVKQLGTCWADGPAYITQCPIQPGQNFIYKFTITGQRGTLLWHAHDSWLRATLHGAIVILPKLGTPYPFP
TPQKERIIILSEWWKSDVEAVVDESTQSGLPPNISDAHTINGHPGPAPGCSSQGFTLHVESGKTYLLRIINAALNDD
LFFKIARHNLTVVEVDASYTKPFQTDTIFISPGQTTTALLEANKDIGNYLISVSPFMDAPIGIDNFTSIATLRYKHT
PRNSTTFLTSIPPQNATPLTATFIDSLRSLDSKLYPANVPLTIDHSLFLTVGVGINPCDTCVNGSKLVASVNNVSFV
MPTIALLQAYYYNIKGVYTLDFPPKPPNPFDYTGTPPVNVQAANGTKLYRLGYNSTVQIVLQDIAIILPESHPTHLH
GFNFFVVGTGLGNFDPDKDTKTFNLVDPVERNTVSVPTGGWTAIRFRADNPGIWFLHCHLEVHTTWGLKMAFLVDNG
EGPNESLTPPPSDLPAC

>FVgene21281
MEIQIAGKLVEEHTDTSPFSTLRRRRGLASTQEDSSSVPDHFPSPASPFFSSFSARSPKTSNIVDLAGKLEKNGLVG
EGKWSGTLLESSGVKARRRRRRRVENGQILQSSKLPEPSYTDHRSPMEVLNSSIFLLGVCLLLASSTMSSAEPKTHN
HEFVIQATPVKRLCKTHKSITVNGQFPGPTLEVNNGDTLVVKVTNKARYNVTIHWHGIRQMRTGWADGPEFVTQCPI
RPGGSYTYRFTIQGQEGTLWWHAHSSWLRATVYGALIIHPKQGDSYPFTKPKREQTLLLGEWWDANPIDVLRQATRT
GAAPNVSDAYTINGQPGDLYKCSSKDTVIVPINSGETNLLRVVNAALNQQLFFAVANHKLTVVSADASYTKPFTTTT
LMLGPGQTTDVLITGDQPPSRYYMAARAYFSAQNAQFDNTTTTAILEYESAPCSNCKNGPSTKPVMPQLPAYNDTNT
ASAFTTSFRSPRKVEVPTDIDENLFFTIGLGLNNCPKNFPSSRCQGPNGTRFTASMNNVSFVLPNNISILQAFQQNI
PGVVTSDFPANPPQQFDYTGNVSRSLWQPKSGTKGYRLNYGSRVQVVLQDTSIVTPENHPIHLHGYDFYVLAEGFGN
FNAKTDTQKFNLVDPPMRNTVAVPVNGWAVIRFVADNPGAWIMHCHLDVHINWGLAMVFLVDNGVGKLQSVEPPPAD
LPLC

>FVgene24290

MEDGDAVLKRAHADYGGYEEDVIVDILARLPVKSLMRFRCVCKSWRALISDPYFVKKHFSWRTETYKLNFNIDPPLF
LDLKALKDVKNEVSFKGIDSIMLWNPCTRDSKVLSNPPLVIEPKDSNYYYHGFGYDSATDDYKVMRGFAYDANGAEK
FMTQIFALKTGSWRTVKDIDYVELTEGQGVFLNGALHWLGDLSDGDRKILSFDLGAEKFQETIPLPYDDWFTDLLIH
RNRLCVCTRPTKSNSCHIWMMKEYGVKESWTELVQFSLDNYVWGPEDHRHYVSPVCILENGVVLIDGLGCYEWLMFA
VFAVLIIILKVLISLAQAEVYYHDFIVKEVNFTRLCESKLMLVVNESYPGPAIRARKGDTIYVNVYNQGYYGFTIHWQ
VPSHNLLFLHGVKQPRNPWFDGPEYVTQCPILPGTNFTYQVQLSTEEGTLWWHAHSDWTRASVHGAIVVLPALGTTF
PFPEPDEEEIIVFGSWYLGNLKEQVDECLEPATNSNLPIAAGYTVNGRLGDFSPCSKNTTYRRKVDYGKTYLLRIVN
ANIDVEFYFAIAEHNLTVVGLDGSYAKPINTDLIVIAPGQTMDVLLNANSTQGRYYYMVGREYLSATVDTASDDDAI
AILEYNGNYTTSEAPLFPEQIPDSLSQAPALNFYRKIRSLATPEYPVNVPKESDVTTRMYITASANALYCTEDLGPT
CFNISFAASVNNISWANPSESILQAYYKNISEPVYETDFPDEPPVYFNFTEIAMTLDPVLTVQGTKVKVLEYNETVE
MVFQGTEVMGGSVNHPMHLHGHSFYVLGFGYGNYFAERDSKTFNLIDPPYVATFITPKNGWLAIRFVANNPGVWFWH
CHMERHLTWGMESAFIIKDGGTPETSMLPPPATMPSCDVPLDSATLPSNSELNKNQIE

>FVgene24292
MATQFLVFAVLIVILLVFSSFAQCEVHYHDFIDITDSPFTGKSLHTTSSSYQHGVKQPRNPWFDGPEYVTQCPILPG
TNFTYQVQLSTEEGTLWWHAHSDWTRASVHGAIVVLPALVTTFPFPEPDEEEIIVFGSWYLGNLKEEVDECLDPATN
SNLPIADGYTINGRLGDFSACSQNTTYRRKVDYGKTYLLRIVNANIDVEFYFAIAEHSLTVVGLDGSYTKPINTGYI
LIAPGQTMDVLLNASSSLGRYYMVGREYISATVETLSDTANDAIAILEYNGNYTTSETPLFPDQIPNSLSQEPAFSF
YGKLRSLATPEYPASVPKESEVTTRMYITASANALYCTEDLGPSCVNISFAASVNNISWANPRESVLQAYYKNISEP
VYETDFPDEPPTYFNFTEIAMTLDPVLTVQGTKVKVLEYNETVEMVFQGTDVMGGSVNHPMHLHGHSFFVLGFGFGN
YLAQRDSKTFNLIDPPCVVLALSYGETFVMGYGVCFYSEERGHTGDYNAPSSGYNAFL

>FVgene24295
MATQFLVFAVLIVILLFDYLFPSSILVVLGTILNRGREPSASNQAIKDEESTNSTVGFFKFALRIRHGVKQPRNPWF
DGPEYVTQCPILPGTNFTYQVQLSTEEGTLWWHAHSDWTRASVHGAIAVLPALGTTFPFPEPDEEEIIVFGTWYLGN
LKEQVDKCLDPATNSDLPKASGYTINGQPGDFSPCSTNTTYRRKVDCGKTYLLRIVNANMDEEFYFAIAEHSLTVVG
LDGSYTKPINTNYIVIAPGQTMDVLLNTSSTLGSYYMVGREYLNSNVDKPIADHDAIAILEYNGNYTTSAAPLFPQQ
IPNSLSQEPAFNFYGKLRSLATPEYPVNVPQESDITTKMYITASSNALYCTPDFDPTCFNISFAASVNNISWANPSE
SILQAYYMNISEPVYETDFPDEPPVYFNFTEKTTTLDRVLTVQGTKVKVLEYNETVEMVFQGTEVMGGSANHPMHLH
GHSFYVLGFGFGNYFAERDSKTFNLIDPPYVSTFITPKKGWLAIRFIANNPGVWFWHCHMERHMTWGMESAFMVKNG
GTPETSMLPPPATMPSCEVPLDSATLPSNSELMKNQIE

>FVgene24296
MSTQFPAFAVLIIIILQLFISLARAEVHYHDFIVKEVNFTRLCESKLMLVVNESFPGPELRARKGDTIYVNVYNQGFY
GFTIHWQVSPLFNSSPICLLHGVKQPRNPWFDGPEFVTQCPISPGTNFTYQINLTTEEGTVWWHAHSDWTRASVHGT
IVVLPALGTTYPFPVPDEEESIVFGSWYMGNLKETIDECMVPENATNLPEAAAYTINGQPGDFALCSANSSYRFKVD
YGKTYLLRILNANIDAEMYFAIAEHNLTLIGMDGAYTKPLESSYIMISPGQTMDLLLNTNISTLGSYYMVAREYVSA
TIDTVSSYLDDAVAILEYNGSYSSLDAPVYPELLPMSLSQEPAYSFIKQIRSLATPEYPINVPTESDVTKRMYITAS
SNALYCTPDLGPSCVNISWAASVNNISWVNPSESILQAYYNNISEPIYETDFPDEPPVYFNFTDKSTAIDLGLTVQG
TKVTMLEYNETVEMVFQGTDVEGASVNHPMHIHGLSFYVLGFGFGNYFAERDSKTFNLIDPPFVATFITPKNGWLAI
RFVADNPGVWFWHCHMERHLTWGMESAFIVKNGGTPETTMLPPPATMPSCDVPLESSAIRDKLERVD

>FVgene25233
MEVEKGKTYLLLIVSAAVNDELFFDIAGHNLTVANQVPSRYFMATRSFMDAPLSINNKTATAILQYKGTQFCSASPS
QLPALNATAFVLSYNGKLRRLDTVKYPENVPLEVDRHLNTSRCFFEQHHFHDAPSWTASSSLLQHHGGIFYRFS

>FVgene25238
MTRSNNFCRASLLLFSCVVGFFFIPAKAALKNYQFDIQVKNVSRLCHSKPIVTVNGMFPGPTIYAREGDTLLVNVTN
HAQYNMSIHWHGLKQYRNGWADGPAYITQCPIKTGNRYTYNMTITGQRGTLWWHAHIFWLRATVYGAIVILPKQGTA
GFPFPQPYSETNLILGEWWNNDVEEVVKQGNKLGLPPNMSDAHTINGKPGPLFPCSEKHTFAMEVEQGKTYLLRIIN
AALNDELFFTIAGHNLTVVEIDAVYTKPFTTQAILIAPGQTTNVLVQANQVPSRYFMAARPFMDAPVSIDNKTATAI
LQYKGIPNSVLPVLSQLPALNDTAFALSYGAKLKSLNTAKYPANVPLKVDRQLFYTIGLGINQCSTCLNGTQLTASL
NNITFVMPKIGLLQAHYFNTKGVFTTDFPDRPPTPFNYTGAPLTANLGTKLGTKLSKIAFNSTVELVLQDTNLLTVE
SHPFHLHGYNFFVVGTGVGNFDPKKDPAKFNLVDPPERNTVGVPTGGWAAIRFRADNPGVWFMHCHLELHTSWGLKT
AFVVENGKGADKSVLPPPADLPPSRHPKMQSSANIHSKKS

>FVgene25266
MAKTNNFGFCSLLPYLCFVAFLFIPAKAAVKSYQFDIQVKNVSRLCHSKPIVTVNGMFPGPTVYAREGDTLLINVTN
HAQYNMSIHWHGLKQYRNGWADGPAYITQCPIKTGSSYIYNMTVTGQRGTLWWHAHIFWLRATVYGAIVVLPKQGTV
FPFPRPYREANLILGEWWNNDVEEVVKQGNRLGLPPNMSDAHTINGKPGPLFPCSEKHTFAMEVEQRKTYLLRIINA
ALNDELFFAIAGHNLTVVEIDAVYTKPFTSQAILIAPGQTTNVLVQANQVPSRYFMAARPFMNAPVSIDNKTATAIL
QYKGIPNSVLPVLSQLPALNDTAFALSYAAKLRSLNTPKYPANVPLKVDRQLFYTIGLGINQCTTCLNGMLEIKWT

>FVgene25408
MTRLCHTKSIVTINGQFPGPRIVAREGDHLLIKVVNHVQSNISIHWHGIRQLQTGWADGPAYITQCPIQTGNSYVYN
FTIVGQRGTLWWHAHISWLRSTIYGPLIILPKRGVPYPFTKPYKEVPIIFGEWWNADTEAVIKQALQTGGGPNVSDA
YTINGLPGPLYNCSAKAQLNNTNVSNGTKLMVLPFNTSVELVLQDTSILGAESHPLHLHGFNFFVVGQGFGNFDQNK
DPKKFNLIDPVERNTVGVPSGGWVAIRFLADNPGVWFMHCHLEIHTSWGLRMAWLVLDGKLPNQKLLPPPLDLPKC

>FVgene27234
MAQNRRLRSTAGCESSKKDRETERRERFEHCGGDSKRVEIWNQQNERPLWWHVHVLWLRATVYGALIIHPRSPRSYP
FPTPFQDVPIMLANRRHRDGNWPQIFPW

>FVgene27522
MGSMLGLLGVFLLDGLLLSMAYNVHHYSFILKETNFTRLCTTKPMLTVNEQFPGPTITVRKGDRAFVNVHNNGKYGV
TIHWHGVKQPQNPWSDGPENITQCPIQPGKNFTYEVIFSDEEGTLWWHAHSDWSRATVHGPIVILPERGKSYPFPEP
YAEKTVVLASWFNRDVKELIDNATAIGGDVDPSDAFTINGWPGALYNCSETISRLPVVFGKTYLFRLVSAVMNEEMF
FAIAGHNFTVVAQDGSYIKPILTSYIMITPGQTMDILLTANQPPSYYYMAASPYFESGAPHDETTTTAILQYTNVIA
APANISMPTLPIETDNISADNFTSKIKALASKDHPISVPKNVTKQILITVSVNQLPCELGAGLCGGPNGNRLSASLN
NISFDTPTTDILQAYYRSLPNVFDSDFPDQPPLYFNFSGVDSDTYLLPQVGTKVKLIEYGASVEIVFQGTNVGNPEN
HPMHLHGYSFYVVGSAYGNWNQTTSPATYNLIDPPAVNTVGVPRNGWTAIRFVADNPGVWFMHCHLERHASWGMDAL
ADGEHPPSDGEQHNSSLIARPAAPHAQPPPHAPGLLHTHSLLLTPSRSARTATVPLPCATPAASVPGLVRCRRKRGC
VRKLRWTELTRAGLLL

>FVgene27523
MGCRILVFFLGTFFLDGLLLSMAANVHHYSFILKETNFTRLCFTKPMLTVNESFPGPTIHVRKGDTAFVNVLNNGKY
GVTIHWHGVKQPRNPWSDGPENITQCPIQPGKNFTYEIIFSDEEGTLWWHAHSDWSRATVHGHIVILPEIGKTYPFP
EPYSQQTIFLGEWFDTDLKELIDEATATGSDPDPSTGFTINGWPGALYNCSNETIYTLGVKYGKTYLLRLVNGVMNE
EMFFGIAGHNITVVAQDASYIKPIVTSYIMITPGQTMDILLTANQTPSYYYMAASPFFDSGAPFDNTTT
TAILRYTNVTSAPSTIPMPSLPFVTDKEGADNFTTQLRALASKKHPISVPKNITERILITVSVNQLPCEANVTCGGP
NGNRLAASLNNISFTTPTTDILQAYYRNLNGVYKDDFPNQPPVYFNFTGVDTDVYLLPDVGTKVKVIEYGASVEIVY
QGTNVGNAENHPMHLHGYSFYVVGSAYGNWNKTTSPATYNLVDPPLVNTVGVPKNGWAAIRFVANNPGVWFMHCHLE
RHTSWGMNSVVIVKNGRTTQTSIRRPPPNLPICIRTSFRRTSTAADSSSVPDHFPSPVSPPNSSFPARSPKTSNKVN
LAGKLEFGGLAGDGKWSGKLLESAGVEARPRRCRTVASGGTSAL

>FVgene27526
MAGFNKMGVILLAASLGFMFLQLCSATRYYDFVLKETNFTRLCSTKSILTVNGSFPGPTIKVHKGETIYVNVHNQGL
YGVTIHWHGVKQPRNPWSDGPENITQCPIQPGSSFTYEVVFSNEEGTLWWHAHSDWTRATVHGAIVILPAAGTKYPF
AAPHAQHPIILGSWFKSDVMEVINSALATGADPNISDAFTINGQPGDLYSCSKASTYRLKVDYGKTYMLRIINAIMN
EEQFFAIANHTLTVVTQDAAYIKPITTSYIMITPGQTMDVLVTANQPRSHYYMASSAYADGIVPFNNSTTTAILQYR
GNYSTPSTIPLPSLPIYNDTTAADSFTALLKSLASKAHPISVPTKITTRIYMTIAINERICPNASCAGPDNNAIAAS
LNNISFETPSIDILQAYYGGINGVFEGNFPSNPPHFYNFTGDYDNSTIYPTFGAKVKFIKYGEGVEIVFQGTNLIAP
ENHPMHLHGFSFYVIGTGYGNFNATESPKTYNLVDPPEVNTVGVPKNGWTTIRFLADNPGVWFMHCHLERHSSWGMD
TVIIVRNGNTTKSKLRPPPAHMPPCSKS

>FVgene27527
MQPKVYLNFSAGAVLGNRYPKRAQLFSLDLKMGCFKPISFFCSVFFFAILACILLLQSEVEAKELYYDFVLKEANFT
KLCSTKSAMVVNGSFPGPVITARKGDTVYVNVHNEGTYGVTIHWHGIKQPRNPWFDGPEYITQCPIQPGTNFTYTVL
LSSEEGTLFWHAHSDWTRATVHGAFVILPAENTTYPFPEPDHEAVLVFASWYKEDVPTLMDEALKYGGLTTLSDSYA
INGQPGDFYECSNETTFRLAVDHGSTYLLRLVNSVQNTDMFFAIADHNLTLVGVDGNYVKPFVTNYLMITPGETMDI
LVTATQPLGHYYMLISPYFDGQADDFDKSVTSAIFQYNGNYTPPTSPIYPDHIPGFYDVDAARNFDRQLKSLASLEH
PANVPQHITTRMFITLSIGMLRCPNDSCDGPDGNRLASALNNISFANPAIDVLQAYYRNMSRYFQADFPDRPPTLFN
FTADELMTDNITLSDQGTKVKMLNYNETVEIIFQGTNVMNSGENHPVHLHGFKFFVVGAGVGNFDNETDPLTYNLID

PPELNTFPVPKDGWAVIRFVADNPGVWFMHCHFDRHMSWGMDTAFIVRNGDTEETSVRPPPEYMPSCGENSIYSGDE
QSMFKLEE

>FVgene27531
MKPQKNRLLVTTEFLGFLFLIAQFTSLLVQGDVHYYDFVVREKEFTKLCVTKSILVVNDSFPGPEIRVHKGDTVFVN
VHNQGYYGFTIHWHGIKQPRNPWSDGPEYITQCPIAPGTNFTYEVQLSDEEGTLWWHAHSDWTRATVHGAIVILPAV
GTTFPFPQQPDEDETTLIIASWYFGDIKELVDVAMEVGGDLPHSDAYTINGEPGDLCECSKETTYRRMVDYGKTYLL
RIVNANINAEHFFAVGGHNLTVVGLDGAYIKPINTGYIVISPGQAMDVLLEANQAPGQYYMAARQYSSEDAAVVDFD
HVNVTAILAYTGNYTYQPPLYPTTLPMYLDYPAAVEFILRIKSLATPEYPISVPLNVTTSMFIVVSMNELPCKHSGC
KTDMEMTSSLNNISFAFPLNTTDVLQAYYRNISGVYTTDFPDQPTQYYNFTDETFEEDILFTVQGTKVKMLDYNETV
QIVFQGTDVMKSSVNHPMHMHGYSFYVVGFGFGNHDNEIDPKGFNLVDPPQVVTFGVPKNGWLAIRFTANNPGMPFP
LYFHF

>FVgene27556
MSHACHLDFGSFPPNPATSLQTLKSVRLTMALLPGFLGALLLSSLAVLCMVEGTVHYYDFVLKEKNFTRLCSTKSML
VVNDMLPGPTIRVQKGDTVYVNVQNQGDFDLTIHWHGVHQPRNPWSDGPEYITQCPIEPGSNFTYEVIFSDEEGTLW
WHAHSDWTRNSVNGAIVVMPLNETGFPFPEPDGEEIIVLGSWYFDDVNEVVAEALQDGTDTPRSDCYTINSQPGDFF
ACSNDSTHRWEVEYGKTYLLRMVNAVMNAELYFAIAEHNLTVVGMDGSYVKPIVTNFTMIAPGQTMDILVTANQSLG
RYYMAARQYDSVRPDVTDYDMSNVTAILEYKGNYTPPELPIFPSTLPTYEDLYSAADFASRIKSLASTEHPINVPQN
ITTRMYITAEMNMITFDNGTGEITGLGSSLNNVSWQDPSTDVLLAYYRNISGFYTSDFPDYPPTFFDFVAEGLPDSV
AATVRGTKVRVLEYNEEVEVVFQGTDVLDASEDHPMHMHGYSYYVVGTGFGNFDNETDPKTYNLVDPPKMNTVTVPK
KGWVAIRFKASNPGVWFWHCHFDRHLSWGMNTVMIVKDGGTPETSMRKPPAYMPPCSDSHTIRLQPFHHTSSRKSK

>FVgene27557
MACSVSLHAYRTRTTKTTSFSVKMGVLQRLLGSLLLCSVFLCLVEGDVHYYDFVLKEANFTRLCMTKSMLVVNDSFP
GPVIRVHKGDTVYVNVQNQGDYGLTIHWHGVHQPRNPWSDGPEYVTQCPIEPGTNFTYEVIFSDEEGTLWWHAHSDW
TRAGVHGAIVVMPSDETGFPFPEPDGEEILVFGSWYYIEDLNAVVAEDVADGSDTPRSDCYTINGQPGDFGLCSKDS
TYRWQVEYGKTYLLRLVSAVMNAELYFAIADHSLTVVGLDGAYVKPIVTDNIMISPGQTMDILVTANQSLGEYYMAA
RQFDSVRPDDQTFDKSNVTAILEYKGNYTPPAYPIFPSTLPSYEDLLPAIEFTNQIKSLASEDHPINVPMNITTKMY
IAVEMNSGSVTETSDDITVSLLASLNNISWVNPTTDVLLAYYRNISGFYASDFPDFPPYFYDFVSDDLPDNTELTLK
ATKVKVLEYNEEVEIVFQGTNTINASENHPMHLHGYSFYLVGSGFGNFDNVRDPQSYNLVDPPKMTTISVPMKGWAA
IRFKASNPGVWLWHCHLDRHFSWGMSFVLIVKDGGEPKTSMREPPPYMPPCTDSPTIRLRPSHKFSRTGK

>FVgene28072
MAQNRRLRSTTGCESSKRDRETERRERFEHCGGDSERVEIWNQQNERPLWWHAHVSWLRATVYGALIIHPRSPRSYP
FPAPFQDVPIMLGEPDAGHISKQTAPNSSSTTTTTTTTTSANAKSSSATNVDSSKLSYSASSNSHSSIAPFSSFSHS
NGSRWSSAAHSSANRAISSPDAVVQQWKPSERILRMKLELTRIQLPCPTPKQYGSLTSFPLTEDVQGSSYYDHG

>FVgene28184
MGASSFTLKPLSCCLLLCLLAFLTLLASFAAAETHYHEFVVEAKPVKRLCRTHNTITVNGQFPGPTLEVRNGDSLAV
KVTNRAKYNVTIHWHGLKQLRNPWADGPEYVTQCPIQPGATYTYRFTIQNQEGTLWWHAHSKWLRATVYGALIIYPR
LGTPYPFSMPKKEFPLLLGEWWDRNPLDVQRLAQFTGGAPNVSDAYTINGQPGTVRIPVESSETILLRIINSALNQE
LFFTIANHRLTVVSADASYTKPFTTRVIMLGPGQTTDVLLTADQPPAHYYAAARAYQTAQNAAFDNTTTTAILEYKS
AAACDSKKAKFSAPVLPSLPAYNDTATATAFSVQIKSPSQVQIPTQIDENLYFIVGLGLINCTVPNSPRCQGPNNTR
FAASMNNVSFVFPKTTSLMQAYYNGIPGVFTTDFPPVPPVQFDYTGNVSRGLWTPSRGTKLYKLKYGSSVQIVLQDT
SIVTTEDHPMHLHGYHFYVVGSGFGNFNPRTDPAKFNLIDPPLRNTIGTNPGGWVAIRFVADNPGVWLMHCHLDAHL
VLGLAMAFVVENGNGPLQSVIPPPADLPQC

>FVgene30568
MFAASINNISFTMPNTALLQSHFFGQSNGVYSPYFPISPLIPFNYTGAQLNNTNVSNGTKLMVLPFNTSVELVLQDT
SILGAESHPLHLHGFNFFVVGQGFGNFDQNKDPKKFNLIDPVERNTVGVPSGGWVAIRFLADNPGVWFMHCHLEIHT
SWGLRMAWLVLDGKLPNQKLLPPPLDLPKC

```
XP_021819117.1      ---------MEFKKMS------WVFAFAGFM-FL--GGICKANGHTHYYDFV--------     34
XP_021833316.1      ---------MEFKKMS------WVLAFVGFM-FL--GGICKANGHTHYYDFV--------     34
XP_021827255.1      --------MGKYNNMS------WVLAFVGFM-LL--GGICKANGAIYNYDFV--------     35
XP_021819124.1      ---------MEFNKMS------WVLAFLGFM-FL--GGICKADGATHFYDFV--------     34
XP_007201720.2      --------MGFYNKMS------WVLAFLGFM-FL--GGISNANGEIHHYDFV--------     35
XP_021819123.1      --------MGFYNKMS------WVLAFLGFM-FL--GGLSNANGAIHHYDFV--------     35
XP_021826148.1      ---------MANGIVH------FAFSVFQL---F--LIFDAGNGKTHRHNFV--------     32
XP_007227301.1      ---------MANGIVH------FAFSVLQL---F--LIFDAANGKTHRHNFV--------     32
XP_021809566.1      ------------MKIL------ISLQLLGLTLLC--VGIFHCQA-WPRYTFV--------     31
XP_021816142.1      ---------MLAMKIL------ISVQLLGLTLLC--VGIFHCQA-WPRYTFV--------     34
XP_020426263.1      --------------------------------------------FFKK--------     4
XP_021820472.1      ---------MLSGKRN------SALQILSLLL-----LFLHCPASSHNYHFV--------     32
XP_007200191.1      ---------MLSGKRN------SALQILSLLL-----VFLHCQASSHNYHFV--------     32
XP_021824778.1      ----------MEALDSIFGNKIRCSLLLGLCLLFASSAMSLAEPKTHQHDFV--------     42
XP_021834606.1      --------------MARFAFVLACAL--------ALLASSLASGAIVEHSFN--------     30
XP_021829543.1      --------------MARFAFVLACAL--------ALLASSLASGAIVEHSFN--------     30
XP_021815052.1      ----------MANLLNSFTIVLSFLFIYGYNYNVHVMAQWPSGGSTRFYDFK--------     42
XP_021803024.1      ----------MECL------IQHYGLFVVMLMILAGALPSGFSQTTRRFQFN--------     36
XP_021825503.1      ---------MGSPHFPCFTSMAALLFG---FFVAASWMPEFAAGKTRHYTFN--------     40
XP_021814279.1      ---------MAFAFPLSPTFVAAFLLALSCFWSLPEFSAAAASGVTRHYRFD--------     43
XP_021809222.1      ---------MGVPLLSSPAFPTLFLFSLIALCLIP---QHALASITRHYKFE--------     40
XP_021809160.1      ---------MGVPLISSPPFLTLFLFSFMMLCLSP---QHALASITRHYKFD--------     40
XP_021829870.1      LQTCHCITIQSRKMV-RTKSFCWGSSALLFLCL-VGFFSTPAKAALKTYRFD--------     50
XP_007198990.1      --------------MA-KTNSFCWGSSALLFLCL-VGFFSTPAKAALKTYQFD--------     37
XP_021834477.1      ------------------------------------------------------------     0
XP_017179926.1      ---------MASICL-RNRFLC--AFMFIFFEF-LGLITSLAEAAIKKYQFDYSVHGFQI     47
XP_021833685.1      ----------MASSF-WNRFFC--GLVFLCFGF-LGLITSPAEAAIKNYQFD--------     38
XP_021833693.1      ----------MASSF-WNRFFC--GLVFLCFGF-LGLITSPAEAAIKNYQFDKRKSCTLC     46
XP_021833835.1      ----------MASSV-RNRLFA--GLLFLCFGF-LGFITSPAEAAIKKYQFD--------     38
XP_008246156.1      ----------MASSR-RNRLFS--GLLFLCFGF-LGFITSPAEAAIKKYQFD--------     38
XP_021833014.1      ---------MESCRL-------HLRAMLLLVAF---LYPAFVQSLVRHYKFS--------     33
XP_021814580.1      ------------MES-------WVRFLVLFLAC---LLPGLVESRIRHYKFN--------     30
XP_021820658.1      ----------MEMVP-------SIRALLLVACL---LFPASVECMVRHYKFN--------     32


XP_021819117.1      -------------LTESNFTRLCETKSILTVNGTLPGPTITVRKGDTAYVNVHNQGSYGL     81
XP_021833316.1      -------------LTESNFTRLCETKSILTVNGTLPGPTITVRKGDTAYVNVHNQGSYGL     81
XP_021827255.1      -------------LREENFTKLCTTKSILTVNGSFPGPTITVRKGDTAFVNVHNQGLYGL     82
XP_021819124.1      -------------LKETNFTRLCSTKSILTVNGSLPGPTITVQKGDTAFVNVHNQGLYGL     81
XP_007201720.2      -------------LKETNFTRLCSTKSILTVNGSLPGPTITVRKGDTAFVNVHNQGLYGL     82
XP_021819123.1      -------------LKETNFTRLCSTKSILTVNGSLPGPTITVQKGDTAFVNVHNQGLYGL     82
XP_021826148.1      -------------VKSSSYTRLCSTKDILTVNGQFPGPSLKAHRGDKMIIKVYNKANYNI     79
XP_007227301.1      -------------VKSSSYTRLCSTKDILTVNGQFPGPSLKAHRGDKMIIKVYNKANYNI     79
XP_021809566.1      -------------VEETPYRRLCSTKNILTVNGQFPGPTLYARTGDTIIVDVYNKGNRNI     78
XP_021816142.1      -------------VEETPYRRLCSTKNILTVNGQFPGPTLYARMGDTIIVDVYNKGNRNI     81
XP_020426263.1      -------------VKEATYTRLCSTKKILTVNGKFPGPVLQAHKGDTIYVNVHNKGSHNI     51
XP_021820472.1      -------------VKEATYTRLCSTKEILTVNGKFPGPVLQAHKGDTIYVNVHNNGRYNI     79
XP_007200191.1      -------------VKEATYTRLCSTKEILTVNGKFPGPVLQAHKGDTIYVNVHNNGRYNI     79
XP_021824778.1      -------------IQATPVKRLCKIQNSITVNGQFPGPTLEVNNGDTLVVKVTNKARYNV     89
XP_021834606.1      -------------VNNLTVTRLCKEQSITVVNGSYPGPTIRARDGDTLVVHVFNQSPYNI     77
XP_021829543.1      -------------VNNLTVTRLCKEQPITVVNGSYPGPAIRARDGDTLVVHVFNQSPYNI     77
XP_021815052.1      -------------VQTKRVTKLCSTKDMVTINGKFPGPSVYAQEDDRIIVNVTNETPFNV     89
XP_021803024.1      -------------VEWKNVTRLCSTKPLLTVNGEYPGPTIAVHEGDNVEIKVTNHIADNT     83
XP_021825503.1      -------------IKYHNVTRLCNTRSILSVNGKFPGPRLVAREGDQVLIKVVNHVSNNI     87
XP_021814279.1      -------------IKLKNVTRLCQTKSIVAVNGKFPGPRIIAREGDRILVKVVNHVQNNI     90
XP_021809222.1      -------------IKQQNVTRLCHTKSIVTVNGQFPGPRIVAREGDRLLIKVVNHVQHNV     87
XP_021809160.1      -------------IKLQNVTRLCQTKSIVTVNGQFPGPRIVAREGDQLLIKVINHVQNNI     87
XP_021829870.1      -------------VQVKNVSRLCHSKPIVTVNGMFPGPTVYAREGDTLLVNVTNHAQYNM     97
XP_007198990.1      -------------VQVKNVSRLCHSKPIVTVNGMFPGPTVYAREGDTLLVNVTNHAQYNM     84
XP_021834477.1      ---------------------------------MFPGPTVYAREGDTLLVNVTNHAQYNM     27
XP_017179926.1      DNI----LFGILQVQVKNVSRLCHAKPIVTVNGRFPGPTIYVREGDRLQINVTNHAQYNM     103
XP_021833685.1      -------------VQVKNVSRLCHAKSIVTVNGRFPGPTIYVREGDRVVINVTNHAQYNM     85
XP_021833693.1      MAFHLITSSVFSQVQVKNVSRLCHAKSIVTVNGRFPGPTIYVREGDRVVINVTNHAQYNM     106
XP_021833835.1      -------------VQVKNVSRLCHAKPIVTINGRFPGPTIYVREGDRVLINVTNHAQYNM     85
XP_008246156.1      -------------VQVKNVSRLCHAKPIVTINGRFPGPTIYVREGDRVLINVTNHAQYNM     85
XP_021833014.1      -------------VVFKNTTKLCSSKPTITVNGKFPGPTLYAREDDTVIVRVINHVNHNL     80
XP_021814580.1      -------------VVLKNTTRLCSTKPIVTINGRFPGPTLYAREDDTVLVKVTNHVKYNV     77
XP_021820658.1      -------------VVQKKTSRLCSSKPIVTVNGRYPGPTIYAREDDTVLIKVVNHVKYNV     79
                                                 *** : .. .*   : * *.   .
```

```
XP_021819117.1      TLHWHGVKQPRNPWYDGPENITQCPIQAGSNFTYWIIFSTEEGTLWWHAHSDWTRATVYG      141
XP_021833316.1      TLHWHGVKQPRNPWYDGPENVTQCPIQAGSNFTYWIIFSTEEGTLWWHAHSDWTRATVYG      141
XP_021827255.1      TIHWHGVKNPRNPWSDGPENITQCPIPAGTNFTHEVIFSSEEGTLWWHAHSDWTRATVHG      142
XP_021819124.1      TMHWHGVKQPRNPWSDGPENITQCPIPVGTNFTYEINFSTEEGTLWWHAHSDWTRATVYG      141
XP_007201720.2      TIHWHGVKQPRNPWSDGPENVTQCPIPAGTNFTYEVIFSNEEGTLWWHAHSDWTRATVYG      142
XP_021819123.1      TIHWHGVKQPRNPWSDGPENVTQCPIPAGTNFTYEVIFSSEEGTLWWHAHSDWTRATVYG      142
XP_021826148.1      TFHWHGVKQPRNPWSDGPEYITQCPIRPGNKYTYKIEFTTEEGTMWWHAHSGWARATVHG      139
XP_007227301.1      TFHWHGIKQPRNPWSDGPEYITQCPIRPGNKYTYKIEFTTEEGTMWWHAHSGWARATVHG      139
XP_021809566.1      TIHWHGAKQPRNPWSDGPDYITQCPIQPGGRFTQTIIFSSEEGTLWWHAHSEWDRATVHG      138
XP_021816142.1      TIHWHGVKQPRNPWSDGPDYITQCPIQPGGSFTQTIIFSSEEGTLWWHAHSEWDRATVHG      141
XP_020426263.1      TIHWHGVKQPRNPWSDGPEYITQCPIQPGDQFKQTIIFSNEEGTIWWHAHNDWARATVHG      111
XP_021820472.1      TIHWHGVKQPRYPWSDGTEYITQCPIQPGHKFKQNIIFSEEEGTIWWHAHSDWSRATVYG      139
XP_007200191.1      TIHWHGVKQPRYPWSDGPEYITQCPIQPGHKFKQNIIFSEEEGTIWWHAHSDWSRATVYG      139
XP_021824778.1      TIHWHGIRQMRTGWADGPEFVTQCPIRPGGSYTYRFTIQGQEGTLWWHAHSSWLRATVYG      149
XP_021834606.1      SIHWHGIFQLLSAWADGPAYVTQCPILPGNNYTYRFNITGQEGTLWWHAHVSWLRATVHG      137
XP_021829543.1      TIHWHGIFQLLSAWADGPAYVTQCPILPGNNYTYRFNITGQEGTLWWHAHVSWLRATVHG      137
XP_021815052.1      TIHWHGVRQKLSCWFDGPSYITQCPIQAGQSFTYEFTMVKQKGTFFWHAHVSWLRATVYG      149
XP_021803024.1      TIHWHGIRQLRTGWADGPAYITQCPIRGGKSYTYKFTVQYQRGTLWWHAHYAWQRASVYG      143
XP_021825503.1      TIHWHGIRQLQTGWADGPAYITQCPIQTGQAYTYNFTITGQRGTLLWHAHISWLRSTLYG      147
XP_021814279.1      SLHWHGIRQLQSGWADGPSYITQCPIQTNQSYVYNFTIIGQRGTLFWHAHISWLRATVYG      150
XP_021809222.1      SIHWHGVRQLRTGWADGPAYVTQCPIQTGNSYVYNFTIVGQTGTLWWHAHISWLRSTLYG      147
XP_021809160.1      SIHWHGIRQLRTGWADGPAYVTQCPIQTGHSYVYNFTIVGQRGTLWWHAHISWLRSTVYG      147
XP_021829870.1      SIHWHGLKQYRNGWADGPAYITQCPIKTGHSYTYNITITGQRGTLWWHAHIFWLRATVYG      157
XP_007198990.1      SIHWHGLKQYRNGWADGPAYITQCPIKTGHSYTYNITITGQRGTLWWHAHIFWLRATVYG      144
XP_021834477.1      SIHWHGLKQYRNGWADGPAYITQCPIKTGHSYTYNITITGQRGTLWWHAHIFWLRATVYG      87
XP_017179926.1      SIHWHGLKQYRNGWADGPAYVTQCPIQTGSSYTYDFNVTGQRGTLWWHAHILWLRATVYG      163
XP_021833685.1      SIHWHGVKQYRNGWADGPAYITQCPIQTGSSYTYDFNVTGQRGTLWWHAHILWLRATVHG      145
XP_021833693.1      SIHWHGVKQYRNGWADGPAYITQCPIQTGSSYTYDFNVTGQRGTLWWHAHILWLRATVHG      166
XP_021833835.1      SIHWHGLKQYRNGWADGPAYVTQCPIQTGSSYTYDFNVTGQRGTLWWHAHILWLRATVYG      145
XP_008246156.1      SIHWHGLKQYRNGWADGPAYVTQCPIQTGSSYTYDFNVTGQRGTLWWHAHILWLRATVYG      145
XP_021833014.1      TIHWHGVKQLGTVWADGPAYITQCPIQPGQNFIYNFTFTGQRGTLLWHAHTSWLRATLHG      140
XP_021814580.1      SVHWHGVRQLQTGWADGPAYITQCPIPPGQSYVYNFTITGQRGTLLWHAHILWLRATVHG      137
XP_021820658.1      SIHWHGVRQLRTGWADGPAYITQCPIQPGQNYVYNFTITGQRGTLWWHAHILWLRATVHG      139
                    :.****  :    * **   :*****  .  :   . .  : **: ****  * *::::*

XP_021819117.1      AIIILPALNTTYPFTTPD-AEETLVLGSWYKGDVNEIIENALATGGDPNVSDAFTINGEP      200
XP_021833316.1      AIIILPALNTTYPFTTPD-AEETLVLGSWYKGDVNEIIENALATGGDPNVSDAFTINGEP      200
XP_021827255.1      AIIILPALNTSYPFVTPD-AQETLILGSWFKGDVNEIIEEALATGGDPNISDAFTINGEP      201
XP_021819124.1      AIIILPADNTTYPFATPD-AQETLILGSWFKGDVMEIIEDALATGGEPNISDAFTINGQP      200
XP_007201720.2      AIVILPALNTTYPFATPD-AQETLVLASWFKGDVVEIIEEALASGGDPNISDAFLINGQP      201
XP_021819123.1      AIVILPALNTTYPFATPD-AQETLILGSWFKGDVMELIEYALASGGDPNISDAFVINGQP      201
XP_021826148.1      AILVYPKPGSHYPFSKPY-AEVPIILGEWWKKNVMEIPRNANITGGEPILSDAYTINGKP      198
XP_007227301.1      AILVYPKPGSHYPFSKPY-AEVPIILGEWWKKNVMEIPRNANITGGEPILSDAYTINGKP      198
XP_021809566.1      AIVIYPKKGATYPFPKPH-AEFPIILGEWWKEDIGQLYNETIQSGGDPNISNAFLINGQP      197
XP_021816142.1      AIVIYPNKGATYPFPKPH-AEFPIILGEWWKEDIGQLYNETIQNGGDPNISNAFLINGQP      200
XP_020426263.1      AIFVYPKRGAGYPFPKPH-EQVQIILGQWWRRDVREVLEEFIRTGGPPNVSDAHTINGQP      170
XP_021820472.1      AIIVYPKRGASYPFPQPH-EEVPIILGQWWKSDVMEVFEEFVRTGGDPNVSDAHTINGQP      198
XP_007200191.1      AIIVYPKRGASYPFPQPH-EEVPIILGQWWKRDIMEVFEEFVQTGGEPNVSDAHTINGQP      198
XP_021824778.1      ALIIHPKQGDSYPFTKPK-RETALLLGEWWNANPINVLRQSTRTGGAPNVSDAYTINGQP      208
XP_021834606.1      ALIIHPKAGRSFPFPKPY-KEIPIILGEWWNGNVVDIENEGLATGIAPNNSNAYSINGLP      196
XP_021829543.1      ALIIHPKAGRSFPFPKPY-KEIPIILGEWWNGNVVDIENEGLATGIAPNNSNAYSINGLP      196
XP_021815052.1      ALIVYPKTGAPYPFKAPY-EEHTLILGEYWLQDVVQLERATAASGGPPTPTNAYTINGHP      208
XP_021803024.1      AFIIHPRM--PFPFSAPIQDEFPIIFGEWWNGDVDEVENEMMKYGGGPNSSDAYTINGLP      201
XP_021825503.1      PIIILPKRNESYPFVKPY-KEVPIILGEWFNVDPEAVISQALQTGGGPNVSDAYTINGLP      206
XP_021814279.1      PLIILPKHNASYPFPKPH-KEVPIIFGEWWKADPEAVIRQALQTGAGPNVSEAYTINGLP      209
XP_021809222.1      PLVILPKLGVPYPFTKPY-TEVPIIFGEWWNADPEAVINQALQTGGGPNVSDAYTFNGLP      206
XP_021809160.1      PLIILPKLGVPYPFTKPY-KAVPIIFGEWWNADPEAVISQALQTGGGPNVSDAYTLNGLP      206
XP_021829870.1      AIVILPKQGTGFPFLQPY-KEANIVLGEWWNNDVEEVVKQGNKLGLPPNMSDAHTINGKP      216
XP_007198990.1      AIVILPKQGTGFPFLQPY-KEANIVLGEWWNNDVEEVVKQGNKLGLPPNMSDAHTINGKP      203
XP_021834477.1      AIVILPKQGTGFPFPQPY-KEANIVLGEWWNNDVEEVVKQGNKLGLPPNMSDAHTINGKP      146
XP_017179926.1      AIVIMPKQGTPFPFAQPY-RETKIILGEWWNVDVETFVNKANSLGLPPNSSDAHTINGKP      222
XP_021833685.1      AIVIMPKQGTPFPFPQPH-REAEIILGEWWNVDVEEFVKKANNLGLAPNTSDAHTINGKP      204
XP_021833693.1      AIVIMPKQGTPFPFPQPH-REAEIILGEWWNVDVEEFVKKANNLGLAPNTSDAHTINGKP      225
XP_021833835.1      AIVILPKQGTPFPFPRPY-REAEIILGEWWNGNVEEFVNKANNLGLPPNSSDAHTINGKP      204
XP_008246156.1      AIVILPKQGTPFPFPQPY-REAEIILGEWWNGNVEEFVNKANNLGLPPNSSDAHTINGKP      204
XP_021833014.1      AIVILPKRGTPYPFPTPD-EEKTVILAEWWKSDVEAVVNQSVQSGLPPNVSDAHTINGHA      199
XP_021814580.1      ALVILPKRGVPYPFPAPH-KEVNVILAEWWKSDTESVINQAMSSGLAPNVSDAHTINGHP      196
XP_021820658.1      ALVILPKLGVPYPFPAPQ-KEVVVILGEWWKSDVESVINQALKSGSAPNVSDAHTINGHP      198
                     :.: *     :**  *      :::..::  :   .       *  *  ::*. :**
```

```
XP_021819117.1      GDLFKCSNATTYRWVVNYGKTYLLRLINAVLNEEMFFAIANHNLTVVAQDGAYLKPITTS      260
XP_021833316.1      GDLYKCSNATTYRWVVDYGKTYLLRLINAVLNEEMFFAIANHNLTVVAQDAAYIKPITTS      260
XP_021827255.1      GDLYDCSNETTYRWLVDYGKTYLLRVINAVMNEEHFFAIANHNLTVVAQDAAYIKPITTS      261
XP_021819124.1      GDLYTCSNETTYRWLVDSGNTYLLRVINAVMNEEQFFAIADHNLRVVAQDAAYIKPITTP      260
XP_007201720.2      GDLYDCSNETTYRLLVDYGKTYLLRVINAVMNEEQFFAIANHTLTVVAQDAAYIKPITTS      261
XP_021819123.1      GDLYDCSNETTYRWLVDYGKTYLLRVINAVMNEEQFFAIANHTLTVVAQDAAYIKPITTS      261
XP_021826148.1      GHLYPCSKSGTFEMTVKHGKTYLLRIISAVMDEELFFGIANHKMILVGRDGSYTKQIETS      258
XP_007227301.1      GHLYPCSKSGIFEMTVEHGKTYLLRIISAVMDEELFFGIANHKMILVGRDGSYTKQVVTS      258
XP_021809566.1      GDLYPCSKPDTVKLMVDYGKTYLLRLINSAVQEILFFSIANHKVTVVGSDASYTKPFSSD      257
XP_021816142.1      GDLYPCSKPDTFKLMVDYGKTYMLRLINSAVQEILFFSIANHKVTVVGSDASYTKPFSSD      260
XP_020426263.1      GDLYPCSKSETFKLLVDQNKTYLLRIVNAAMNTIFFYSIANHNLTVVGVDGSYTKPVTTD      230
XP_021820472.1      GDLYPCSKSETFKLFVDENKAYLLRIINAAMNSILFFSIANHNLTVVGADGSYTKPVTRD      258
XP_007200191.1      GDLYPCSKSETFKLFVDENKAYLLRIINAAMNSILFFSIANHNLTVVGADGSYTKPVTRD      258
XP_021824778.1      GDLYNCSSQDTIIVPIDSGETNLLRVINAALNQPLFFTVANHKLTVVSADASYTKPFTTT      268
XP_021834606.1      GDLYDCSQNQTYQVKVVKGKTYLLRIINAALNNQLFYKIANHNMTVVAIDAAYTTPYITD      256
XP_021829543.1      GDLYDCSQNQTYQVKVVKGKTYLLRIINAALNNQLFYKIANHNMTVVAIDAAYTTPYITD      256
XP_021815052.1      GPNYNCSNNDVFQIDVVSGKTYLLRLIHAGLNMENFFAIANHKLTIVEADAEYTKPFTTD      268
XP_021803024.1      GPFYPCSNKDTFIKTVERGKTYLLRIINAALNDELFFAVVNHTLTVVEIDAVYTKPFTAT      261
XP_021825503.1      GPLYNCSSKDVFKLKVKPGKTYLLRLINAALNDELFFSIANHSLTVVEADAVYTKPFETD      266
XP_021814279.1      GPLYNCSKKDTFRLKVKPGKTYLLRLINAAVNDELFFSIANHSLTVGEADALYVKPFKTD      269
XP_021809222.1      GPLYNCSAKDAFKLKVKSGKTYLLRLINAALNDELFFSIANHTLKVVEADAVYVKPFETD      266
XP_021809160.1      GPLYNCSAKDAFKLKVEAGKTYLLRLINAALNDELFFSIANHTLQVVEADAVYVKPFETH      266
XP_021829870.1      GPLFPCSEKHTYALEVEQGKTYLLRIINAALNDELFFAIAGHNLTVVEIDAVYTKPFTSQ      276
XP_007198990.1      GPLFPCSEKHTYALEVEQGKTYLLRIINAALNDELFFAIAGHNLTVVEIDAVYTKPFTSQ      263
XP_021834477.1      GPLFPCSEKHTYALEVEQGKTYLLRIINAALNDELFFAIAGHNLTVVEIDAVYTKPFTSQ      206
XP_017179926.1      GPLFPCSEKHTFAMEVEQGKTYLLRIINAALNDELFFAIAGHNLTVVEVDALYTKPFTTQ      282
XP_021833685.1      GPLFPCSEKHTFVMEVEQGKTYLLRIINAALNDELFFAIAGHNLTVVEVDAVYTKPFTTQ      264
XP_021833693.1      GPLFPCSEKHTFVMEVEQGKTYLLRIINAALNDELFFAIAGHNLTVVEVDAVYTKPFTTQ      285
XP_021833835.1      GPLFPCSEKHTFAMEVEQGKTYLLRIINAALNDELFFGIAGHNLTVVEVDAVYTKPFTTE      264
XP_008246156.1      GPLFPCYEKHTFVME---------------------------------------------      219
XP_021833014.1      GPVPGCSSPGGYTLHVESGKTYLLRIINAALNDDLFFKIAGHNLTVVEVDASYTKPFQTD      259
XP_021814580.1      GPVANCSSQGGFTLPVESGKTYLLRIINAALNEELFFKIAGHKLTIVEVDATYVKPFKTD      256
XP_021820658.1      GPLSTCSSQGGFKLPVRPGKTYMLRIINAALNEELFFKIASHKLTVVEVDAVYTKPFKTD      258
                    *    *

XP_021819117.1      YLMITPGQTMDILVVANQS---PSSYHVASGPFVDGDVAFNKNGNTTAILQYNGS-----      312
XP_021833316.1      YLMITPGQTMDILVVANQS---PSSYHAASAPFVDGDVAFNNS-TTTAILQYNGS-----      311
XP_021827255.1      YVMITPGQTMDILVTANQS---PSHYYIASTSFVDGDVAFNNS-TTTAILQYNGN----Y      313
XP_021819124.1      YIMITPGQTMDILVTANQP---PSHYYIASTSFVDGNVTFNNS-TTTAIVQYNGN----Y      312
XP_007201720.2      YIMITPGQTMDILVTANQP---LSHYYIASKAFVDGVVPFNNS-TTTAIVQYNGN----Y      313
XP_021819123.1      YIMITPGQTMDILVTANQP---PSHYYIASKAYVDGTVAFNNS-TTTAIVQYNGN----Y      313
XP_021826148.1      YIMIAPGQSMDVLLQANQP---PSFYFMAARAYSSAIGAGFDKTMTTAILKYQASSHEP-      314
XP_007227301.1      YIMIAPGQSMDVLLQANQP---PSFYFMAARAYSSAIGAGFDKTMTTAILKYQASYHEP-      314
XP_021809566.1      YVTISPGQTIDLLFTADQS---PNHYYIAAKAYVGGAGIVYDNTTTTAILQYNGN----Y      310
XP_021816142.1      YVTISPGQTIDLLFTADQT---PNHYYIAAKAYVGGAGIVYDNTTTTAILQYNGN----Y      313
XP_020426263.1      YMTISPGQTVDAVLITNQQ---VGQYYMAARAYSSTLLIPFDNTTTTAIVEYKKIGNNFT      287
XP_021820472.1      YITISPGQTLDALLLTNQQ---VGQYYMAARAYSSSPTIAFDNTTTTAIVQYNN--RNST      313
XP_007200191.1      YITISPGQTLDALLLTNQQ---VGQYYMAARAYSSSPAVAFDNTTTTAIVQYNN--RNST      313
XP_021824778.1      VLMLGPGQTTDVLITGDQP---PARYYLAARAYFSAQNAAFDNTTTTAILEYKSAPCSPN      325
XP_021834606.1      VVVVAPGQTTDVLITANQQ---TGSYYMAATPYMSA-NINFDNTTTRGIIVYENS-----      307
XP_021829543.1      VVVVAPGQTTDVLITANQQ---TGSYYMAATPYMSA-NINFDNTTTRGIIVYENS-----      307
XP_021815052.1      RVMLGPGQTMSVLVTADQS---PGKYSMAMGPYMSAKGVKFQNISSIAYFQYSGAVPNSL      325
XP_021803024.1      AIMVAPGQTTNVLLTANQVPDSSGTFLMAAWPYLTSI-FPFDNSTTAGFLRYKNTVTENI      320
XP_021825503.1      TLLIAPGQTTNLLLKTKPTS-PNATFLILARPYFTGA-GTLDNTTTAGILEYKLPNTTHH      324
XP_021814279.1      ILLITPGQTTNVLLRTKPSY-PNATFLMLARPYFTGM-GTFDNSTVAGILEYKNPSNPSA      327
XP_021809222.1      TILITPGQTTNVLLKTKPHF-PNATFFMTARPYVTGL-GTFDNSTVAGILEYESPSKTLH      324
XP_021809160.1      TILIAPGQTTNVLLKTKPSF-PNATFFLTARPYVTGL-GTFDNSTVAGILEYEFPPNT--      322
XP_021829870.1      AILIAPGQTTNVLVQANQV---PGRYFMAARPFMDAP-VSIDNKTATGILQYKGIPNTVQ      332
XP_007198990.1      AILIAPGQTTNVLVQANQV---PGRYFMAARPFMDAP-VSIDNKTATGILQYKGIPNTVQ      319
XP_021834477.1      AILIAPGQTTNVLVQANQA---PGRYFMASRPFMDAP-ISIDNKTATGILQYKGIPNTVQ      262
XP_017179926.1      AILIAPGQTTNVLVKANQA---SGRYFMAARPFMDAP-VPVDNKTATAIFQYRGVPNTVL      338
XP_021833685.1      AILIAPGQTTNVLVQANQA---SGRYFMATRPFMDAP-VPIDNKAATGIFQYRGIPNTVL      320
XP_021833693.1      AILIAPGQTTNVLVQANQA---SGRYFMATRPFMDAP-VPIDNKAATGIFQYRGIPNTVL      341
XP_021833835.1      AILIAPGQTTNVLVQANQA---SGRYFMAARPFMDAP-VAIDNKTATGIFQYRGIPNIVL      320
XP_008246156.1      ------------------------------------------------------------      219
XP_021833014.1      TIFISPGQTTNAILTANQG---IGKYLIAASPFMDAP-VGFDNLTSIASLRYKGTPANPK      315
XP_021814580.1      TIVIAPGQTTNALITANQK---SGKYLVAASPFMDSP-IAVDNLTATATLHYSGTLATTS      312
XP_021820658.1      TVLIAPGQTTNVLLSTNHG---TGKYLVAASPFMDNPNILVDNKTGTATLHYTGTLESTR      315
```

```
XP_021819117.1      TTPS--------TIPTPTLPNPTDGT------AASNFTMQVRALASKDYPISVPLNITHT      358
XP_021833316.1      TTPS--------TIPTPTFPNPADET------AASNFTTQVRALASKDYPISVPLNITHT      357
XP_021827255.1      SAPS--------TIPLPNLPDHDDDT------AAANFTTQVRALASEEHPISVPVNISHR      359
XP_021819124.1      STPS--------TTPFPTLPDHDDDT------AAENFTKQVRALASEDHPISVPLNISTS      358
XP_007201720.2      SAPS--------TPSFPTLPDYDNQT------AADNFTSQIRALASEEHPISVPLEIHSK      359
XP_021819123.1      SAPS--------TTPFPTLPDYDNET------AADNFTSQIRALASKEHPISVPLEIDSK      359
XP_021826148.1      PKFP--------HYKFPHLPPYDRTQ------ASTDFTKRIRSLATKDHPTNVPLHVNTH      360
XP_007227301.1      PEFP--------HYKFPHLPPYDRTQ------ASTDFTKRIRSLATKDHPTNVPLHVNTH      360
XP_021809566.1      TSTS--------TPSFPNLPSHNDTK------ASVHFTGSLRSLADQNHPVDVPREITTS      356
XP_021816142.1      TSTS--------TPSFPNLPSHNDTK------ASVHFTGSLRSLADQNHPVDVPREITTS      359
XP_020426263.1      PFSS--------TPPLPHLPYYNDTN------AAFTFYDSLKSLANEHHPIDVPIKITNR      333
XP_021820472.1      PFSS--------PPILPYLPYYNDTN------AAFNFFDSLRSLANEDHPIDVPKNITTR      359
XP_007200191.1      PFSS--------PPILPYLPYYNDTN------AAFNFFDSLRSLANEDHPIDVPKNITTR      359
XP_021824778.1      CTNGPTV-----KPIMPPLPAFNDTA------TASAFTTSF----RSPRKVEVPTEIDEN      370
XP_021834606.1      ----TSS-----SPIMPALPNPSDTP------TAHKFNTNITGLAGGPQWVPVPTNVDEH      352
XP_021829543.1      ----TSS-----SPIMPALPNPSDTP------TAHKFNTNITGLAGGPQWVPVPTNVDEH      352
XP_021815052.1      S-------------LPAKLPCFNDNL------AVKTVMDGLRSLN----PVNVSRQIDTD      362
XP_021803024.1      KH--LQKMPSTFEPNKYNLPKMEDTK------FVTKFSDKLRSLASPQYPCTVPKTIDKR      372
XP_021825503.1      --TSIKALP----LFRPSLPPINATKFVNVTQFVENLAKKFRSLATKQFPANVPQTVDRR      378
XP_021814279.1      -STSLKNPP----LLKPTLPQINATS------FVANFSAKFRSLANSKFPANVPKTVRKR      376
XP_021809222.1      STLPMKKLP----LFKPILPALNDTS------FATKFSNKLRSLANAQFPANVPQKVDKH      374
XP_021809160.1      KTLKMKKLP----LFKPDLPALNDTS------FATKFNDKLRSLNNAPFPANVPQKVDKH      372
XP_021829870.1      P-------------VLPQLPALNNTA------FALSFNAKLRSLNTAQFPASVPLKVDRH      373
XP_007198990.1      P-------------VLPQLPALNNTA------FALSFNAKLRSLNTAQFPASVPLKVDRH      360
XP_021834477.1      P-------------VLPQLPALNDTA------FALSFNAKLRSLNTAQFPASVPLKVDRH      303
XP_017179926.1      P-------------SLPQLPNPNDTS------FALSYNKKLRSLNSPNFPANVPLKVDRK      379
XP_021833685.1      P-------------SLPQLPASNDTS------FALSYGKKLKSLNTPNFPANVPLKVDRK      361
XP_021833693.1      P-------------SLPQLPASNDTS------FALSYGKKLKSLNTPNFPANVPLKVDRK      382
XP_021833835.1      P-------------SLPQLPASNDTA------FALSYNKKLKSLNTPNFPANVPLKVDRK      361
XP_008246156.1      ------------------------------------------------------------      219
XP_021833014.1      A-------------FLTSIPPQNATP------VTNTFMNALRSLNSKQYPANVPLTIDHS      356
XP_021814580.1      T-------------TLTNPPPQNATQ------VANNFINSLKSLNSKKFPAKVPLKIDHN      353
XP_021820658.1      T-------------TLTAPPPQNATL------VATKFTNSLRSLNSVKFPARVPLKIDHS      356


XP_021819117.1      LFISVSVNERICPN-----SSCDGPN-DNALAASLNNISFVTP-SIDILQAYYGKI-YGV      410
XP_021833316.1      LFISVSVNERICPN-----SSCDGPD-GNALAASLNNISFVTP-SIDILQAYSGAI-YGV      409
XP_021827255.1      LFITVSINERICPN-----SSCDGPD-NNALAASLNNISFVTP-SIDILQAYYGGI-NGV      411
XP_021819124.1      MYIAVSINERICAN-----SSCAGPD-GNALSASLNNISFQTP-SISVLQAYYGGNTNGV      411
XP_007201720.2      IIISIAINERICPD-----SNCSGPH-NNAISASLNNISFELP-SIDILQAYYGGNNNGV      412
XP_021819123.1      IIISIAINERICPN-----SSCSGPD-GNALSASLNNISFELP-SVDILQAYYRGNNNGV      412
XP_021826148.1      LFFTISVNLLNCSN-----KPCTGPF-GKRFAASVNNISFVAP-SIDILQAYYYKI-PGV      412
XP_007227301.1      LFFTISVNLLNCSN-----KPCTGPF-GKRFAASVNNISFVAP-SIDILRAYYYKI-PGV      412
XP_021809566.1      LFYTLSVNTLPCLK-----NSCAGPN-GTRLAASVNNISFVDP-SIDILQAYYYHV-NGA      408
XP_021816142.1      LFYTLSVNTLPCLK-----NSCAGPN-GTRLAASVNNISFVDP-SIDILQAYYYHV-NGA      411
XP_020426263.1      LVFAVSINTFPCSN-----NSCAESN-GTRLAASMNNISFVSPKTIDILEAYYYHI-HGI      386
XP_021820472.1      LISTVSVNTFPCPN-----NSCEGPN-GTRLAASMNNISFVDPTTIDILEAYYYHI-NGV      412
XP_007200191.1      LISTVSVNTFPCPN-----SSCEGPN-GTRLAASMNNISFVDPTTIDILEAYYYHI-NGV      412
XP_021824778.1      LFFTIGLGLNNCPKHFNKTRRCQGPN-GTRFTASMNNVSFVLPNNISILQAYQQNI-PGV      428
XP_021834606.1      MFVTVGVNLELCPVN----ATCQGPF-NNRLSASMSNESFQLPTNLSMMQAQFYNV-SGI      406
XP_021829543.1      MFVTVGVNLELCPVN----ATCQGPF-NNRLSASMSNESFQLPTNLSMMQAQFYNV-SGI      406
XP_021815052.1      LFVTIGLNVQKCHSKTP-KQNCQGLNNG-VMAASMNNISFVKPK-ISLLEAYYKKI-NGS      418
XP_021803024.1      VVTVVSLNLQDCPA----NKTCKGYE-GKRFFASMNNLSFVRPS-MSILESYYNKLRNNE      426
XP_021825503.1      FFFTIGLGTSPCPQ----NSTCQGPN-GLKFAASVNNFSFALPS-TAMLQAHFSGQSNGV      432
XP_021814279.1      FFFTVGLGTNPCPK----NQTCQGPNNSTKFAASINNISFILPT-TALLQSHFFGKSNGV      431
XP_021809222.1      FFFTVGLGTNPCNKH---NQTCQGPN-GTMFAASVNNVSFVMPT-TALLQAHFSGQSNRV      429
XP_021809160.1      FFFTVGLGTNPCSQH---NQTCQGPN-GTMFAASINNISFAMPN-TALLQSHFSGQSNGV      427
XP_021829870.1      LFYTIGLGINQCTT-------CHN---GTQLTASLNNITFVMPQ-IGLLQAHYFNT-KGV      421
XP_007198990.1      LFYTIGLGINQCTT-------CLN---GTQLTASLNNITFVMPQ-IGLLQAHYFNT-KGV      408
XP_021834477.1      LFYTIGLGINQCTT-------CLN---GTQLTASLNNITFVMPQ-IGLLQAHYFNT-KGV      351
XP_017179926.1      LFYTIGFGKESCPS-------CIN---GTRFVASLNNISFEMPQ-VGLLQAHYFNL-KGV      427
XP_021833685.1      LFYTIGFGKEPCPT-------CLN---GTRFVASLNNISFEMPQ-VGLLQAHYFNL-KGV      409
XP_021833693.1      LFYTIGFGKEPCPT-------CLN---GTRFVASLNNISFEMPQ-VGLLQAHYFNL-KGV      430
XP_021833835.1      LFYTIGFGKESCPT-------CLN---GTRFVASLNNISFEMPQ-VGLLQAHYFNL-KGV      409
XP_008246156.1      ------------------------------------------------------------      219
XP_021833014.1      LFFTIAVGVNPCAT-------CVN---GSKLVAAFNNVSFVMPT-IALLQAYYYNK-KGV      404
XP_021814580.1      LLFTVGLGINPCPT-------CKAG-NGSRVVASVNNVTFVMPT-TALLQAHVFNI-SGV      403
XP_021820658.1      LLFTVGLGLHTCAS-------CAN---GNRLVANINNVTFVMPK-ISLLQAHFFNI-SGV      404
```

```
XP_021819117.1      YSANFPH-------K-PYIFNFTGHV-RND--TIYPNFGTKVRMIEYGEEVEIIYQGTNM      459
XP_021833316.1      YSANFPH-------K-PYIFNFTGHV-RND--TIYPNFGTKVRTIEYGEEVEIIYQGTNM      458
XP_021827255.1      YTPNFPD-------KPPYFFNFTGLV-QNN--TIYPSFATKVKMIKYGKEVEIIFQGTSM      461
XP_021819124.1      YATNFPD-------IPPYFYNFTGDF-ANN--TLYPSFGTRVRMINYGEGVEIVFQGTSV      461
XP_007201720.2      YRTSFPN-------KPPYYFNFTGDV-GNN--TLYPNFGTRVRMIKYGEGVEIVFQGTNI      462
XP_021819123.1      YSTSFPN-------KPPYTFNFTGDV-GNN--TIYPSFGTRVRMIKYGEGVEIVFQGTNI      462
XP_021826148.1      FENDFPKKLLNFXRKPPKEFNYTGEDLPEN--LLTPSYGTKVVVLEYNASVELVLQGTNV      470
XP_007227301.1      FENDFPK-------KPPMEFNYTGEDLPEN--LLTPSYGTKVVVLEYNASVELVLQGTNV      463
XP_021809566.1      FGTRFPN-------FPPFLFNFTAQDLPLY--LQTPKQGTEVKILEYNATVEIVFQGTNL      459
XP_021816142.1      FGTRFPN-------FPPLLFNFTAQDLPLY--LQTPKQGTEVKILKYNATVEIVFQGTNL      462
XP_020426263.1      YKKGFPD-------FPPLVFNFTGENLPLF--LRVPKRGTKVRVFKYGSIVELVFQGTNL      437
XP_021820472.1      FREGFPD-------FPPLVYNFTSEDLPLI--LQTPKRGTKVKVFDYGSIVECVFQGTSL      463
XP_007200191.1      FREGFPD-------FPPLVYNFTGEDLPLI--LQTPKRGTKVKVFDYGSIVECVFQGTNL      463
XP_021824778.1      FTADFPA-------NPPLKFDYTGNVSR---SLWQPLSGTRGYKLKYGSRVQVVLQDTSI      478
XP_021834606.1      YTTDFPD-------QPLVKFDYTDSNISLDLSLIYAPKSTKVKTLKFNSTVEIVLQNTAF      459
XP_021829543.1      YTTDFPD-------QPAVKFDYTDSNISLDLSLIYAPKSTKVKTLKFNSTVEIVLQNTAF      459
XP_021815052.1      FTEDFPG-------VPLKFYDFVNGAPNNVPNNTQALNGTKALVLEYGARVQLIMQDTGT      471
XP_021803024.1      YSTDFPE-------KPLKPFDYTGVDTVSE--NMNTQFGTKILEVPYGTNLEIVLQGTSF      477
XP_021825503.1      YTTDFPT-------KPLVAFNYTGTPPNIT----NVSNATRAVVLPFNTSVELVLQDTSI      481
XP_021814279.1      FTTDFPT-------NPVQPFNYTGTPSNNT--NINVSNGTKAVVLKFNTSVEVVLQGTGI      482
XP_021809222.1      YSSNFPI-------SPVIPFNYTGTPPNNT----MVSNGTKLVVLPFNTSVELIMQDTSI      478
XP_021809160.1      YTPDFPT-------NPIIPFNYTGAPPNDT----MVSNGTKLVVLPFNTSVELVMQDTSI      476
XP_021829870.1      FTTDFPD-------RPPTPFNYTGAPL-TA--NLGTKLGTRLSKLAFNSTVELVLQDTNL      471
XP_007198990.1      FTTDFPD-------RPPTPFNYTGSPL-TA--NLGTKLGTRLSKLAFNSTVELVLQDTNL      458
XP_021834477.1      FTTDFPD-------RPPTPFNYTGAPL-TA--NLGTKRGTRLSKLAFNSTVELVLQDTNL      401
XP_017179926.1      FKTDFPD-------RPSTVFNYTGAPL-TA--NLGTSTGTRLSKIAFNSTVELVLQDTNL      477
XP_021833685.1      FKTDFPD-------RPQTPFNYTGAPL-TA--NLGTSTGTRLSKIAFNSTVELVLQDTNL      459
XP_021833693.1      FKTDFPD-------RPQTPFNYTGAPL-TA--NLGTSTGTRLSKIAFNSTVELVLQDTNL      480
XP_021833835.1      FKTDFPD-------RPQTPFNYTGTPL-TA--SLGTSTGTRLSKIVFNSTVELVLQDTNL      459
XP_008246156.1      ------------------------------------------------------------      219
XP_021833014.1      YTLDFPA-------NPPIPFNYTGNSTA----NMQTTNGTRLYRLGYNSTVQIVLQGTAI      453
XP_021814580.1      FTTDFPG-------NPPNTFNFSGGPPANA--SMATTNGTKLYRLAYNSTVQLVLQDTGT      454
XP_021820658.1      FTDDFPG-------NPLNPYNYTGPQPT----NMQTMKGTRLYRLAYNSTVQLVLQDTGM      453


XP_021819117.1      IAAENHPMHLHGFSFYLVGTGTGNFDPNQAPKTYNLVDPPEVNTIGVPKNGWATVRFKAD      519
XP_021833316.1      IAAENHPMHLHGFSFYLVGTGSGNFDPNQAYKTYNLVDPPEVNTIGVPKNGWATVRFKAD      518
XP_021827255.1      IAAENHPMHLHGFSFYLVGTGSGNFNSTTSPKTYNLVDPPEVNTIGVPKKGWAAIRFVAD      521
XP_021819124.1      IAAENHPMHLHGFSFYLVGTGSGNFNSTTSPTTYNLVDPPEVNTIGVPKNGWAAIRFVAD      521
XP_007201720.2      IAPENHPMHLHGFSFYVVGTGYGNFNSTTSPKTYNLVDPPEVNTIGVPKNGWAAIRFVAD      522
XP_021819123.1      IAPENHPMHLHGYSFYVVGTGSGNFNSTTSPKTYNLVDPPEVNTIGVPKNGWAAIRFVAD      522
XP_021826148.1      LASDNHPVHLHGYSFYVVGWGFGNFNPKKDPLSYNLVDPPEETTVGVPKNGWVAIRFRVD      530
XP_007227301.1      LASDNHPVHLHGYNFYVVGWGFGNFNPKKDPLNYNLVDPPEENTVGVHKNGWVAIRFRAD      523
XP_021809566.1      VAGDDHPMHLHGFSF---------------------------------------------      474
XP_021816142.1      VAGDDHPMHLHGFSFYVVGWGLGNFDEDKDPLTYNLVDPPLQNTIAVPVNGWTTIRFKAD      522
XP_020426263.1      GAPIDHPIHLHGFSFYIVGWGFGNFDKDKDPLNYNLIDPPLRNTVAVPISGWAAIRFHAN      497
XP_021820472.1      VAGIDHPMHLHGFSFYIVGTGFGNFDQDKNPLNYNLVDPPHRNTVTVPINGWTTIRFKVK      523
XP_007200191.1      VAGIDHPMHLHGFSFYIVGRGFGNFDQDKDPLNYNLVDPPHRNTVIVPINGWTAIRFKTN      523
XP_021824778.1      VTPENHPIHLHGYDFYILAEGFGNFNAQTDTKKFNLVDPPMRNTVAVPANGWAVIRFVAD      538
XP_021834606.1      LAIENHPIHLHGFNFHVLAQGFGNYDPINDPKKFNLVNPQIRNTIGVPVGGWAVIRFTAN      519
XP_021829543.1      LAIENHPIHLHGFNFHVLAQGFGNYDPINDPKKFNLVNPQIRNTIGVPVGGWAVIRFTAN      519
XP_021815052.1      VTTENHPIHLHGYSFYVVGYGTGNYNPK--TANFNLVDPPYMNTIGVPVGGWAAIRFVAD      529
XP_021803024.1      LNVENHPIHIHGHNFFIVGRGFGNFNVAKDPAKYNLIDPPERNTVAVPTGGWAAIRFKAD      537
XP_021825503.1      VGAESHPLHLHGFNFFIVGLGFGNYDPTKDPANFNVVDPAERNTAGVPAGGWLAIRFFAD      541
XP_021814279.1      LGAESHPLHLHGFNFFVVGQGFGNFNPNKDPASFNLVDPVERNTIGVPSGGWVAIRFLAD      542
XP_021809222.1      LGAESHPLHLHGYNFFVIGQGFGNFDQNKDPANFNLVDPVERNTVGVPSGGWVAIRFLAD      538
XP_021809160.1      LGAESHPLHLHGFNFFVVGQGFGNFDRNKDPAKFNLVDPVERNTVGVPSGGWVAIRFLAD      536
XP_021829870.1      LTVESHPFHLHGYNFFVVGTGVGNFDPKKDPAKYNLVDPPERNTIGVPTGGWVALRFRAD      531
XP_007198990.1      LTVESHPFHLHGYNFFVVGTGVGNFDPKKDPAKYNLVDPPERNTIGVPTGGWVALRFRAD      518
XP_021834477.1      LTVESHPFHLHGYNFFVVGTGVGNFDPKKDPAKYNLVDPPERNTIGVPTGGWVAIRFRAD      461
XP_017179926.1      LTVESHPFHLHGYNFFVVGNGIGNFDPAKEPAKYNLVDPVERNTVGVPTGGWTAIRFRAD      537
XP_021833685.1      LTVETHPFHLHGYNFFVVGTGIGNFDPAKDPAKYNLVDPVERNTVGVPTGGWTAIRFRAD      519
XP_021833693.1      LTVETHPFHLHGYNFFVVGTGIGNFDPAKDPAKYNLVDPVERNTVGVPTGGWTAIRFRAD      540
XP_021833835.1      LTVESHPFHLHGYNFFVVGTGIGNFDPAKDPAKYNLVDPVERNTVGVPTGGWTAIRFRAD      519
XP_008246156.1      ------------------------------------------------------------      219
XP_021833014.1      IAPESHPTHLHGFNFFVVGKGIGNFDPKKDPKTFNLVDPIERNTISVPTGGWTAIRFRAD      513
XP_021814580.1      IAPENHPVHLHGYNFYSVGRGVGNYNPKKDPKKFNLVDPVERNTVGVPSGGWAAIRFQAD      514
XP_021820658.1      IAPENHPVHLHGFNFFEVGRGLGNFNPKKDPKRFNLIDPVERNTIGVPSGGWTAIRFRAD      513
```

```
XP_021819117.1      NPGVWFMHCHLERHASWGMATVLIVTNGNTNETSMLPAPAYMPPCT--------------      565
XP_021833316.1      NPGVWFMHCHLERHASWGMATVLIVTNGNTNETSMLPAPAYMPPCT--------------      564
XP_021827255.1      NPGVWYMHCHLERHSSWGMNTVLIVRNGNTTETSILPPPAYMPPCSKS------------      569
XP_021819124.1      NPGVWFMHCHLERHSSWGMDTVLIVRNGNTSESTIRGPPAYMPPCSNS------------      569
XP_007201720.2      NPGVWFMHCHLERHSSWGMDTVLIVRNGNTTESKIRPPPPAMAGGRRKKKKMEIKDTNFT      582
XP_021819123.1      NPGVWFMHCHLERHSSWGMDTVLIVRNGNTTERKIRSPPAYMPPCSKS------------      570
XP_021826148.1      NPGVWLMHCHIERHQTWGMTVVLLVKNGISPQNKILLPPHDLPTC---------------      575
XP_007227301.1      NPGVWLMHCHIERHQTWGMTVVFLVKNGNSPQNKILPPPHDLPTC---------------      568
XP_021809566.1      ------------------------------------------------------------      474
XP_021816142.1      NPGVWFMHCHLDRHMSWGMDMTFIVKNGKGLGAKILPPPPGMPPC---------------      567
XP_020426263.1      NPGVWFLHCHLEHHLTWGMNTVFIVKDGKNKKERLLPPPPRMPPC---------------      542
XP_021820472.1      NPGVWFLHCHLERHLTWGMNTVFIVKNGKHNKEKLLPAPQEMPPC---------------      568
XP_007200191.1      NPGVWFLHCHLERHLTWGMNTVFIVKNGKHKKEKLLPAPQEMPPC---------------      568
XP_021824778.1      NPGAWIMHCHLDVHINWGLAMVFLVDNGVGTLQSIEAPPADLPLC---------------      583
XP_021834606.1      NPGMWFMHCHLDVHLPWGLGMVFEVENGPTP-WVLPAPPADLPQC---------------      563
XP_021829543.1      NPGMWFMHCHLDVHLPWGLGMVFEVENGPTP-WVLPAPPADLPQC---------------      563
XP_021815052.1      NPGVWFMHCHLDIHQSWGLGTVLVVKNGKGDLEILPHPPADLPQC---------------      574
XP_021803024.1      NPGVWFIHCHLEEHTSWGLASGLIVQNGPRASQCVLPPPKDLPSC---------------      582
XP_021825503.1      NPGVWFMHCHLDIHTSWGLRMAWIVQDGPQPNQKLPPPPSDLPKC---------------      586
XP_021814279.1      NPGVWLMHCHFDVHLSWGLRMAWVVQDGKLPNQKLPPPPSDLPKC---------------      587
XP_021809222.1      NPGVWFMHCHLEIHTSWGLKMAWVVLDGKLPNQKLLPPPADLPTC---------------      583
XP_021809160.1      NPGVWFMHCHLEIHTSWGLKMAWVVLDGKLPNQKLLPPPADLPKC---------------      581
XP_021829870.1      NPGVWFMHCHLELHTSWGLKTAFVVENGKDSDHSVLPPPTDLPPC---------------      576
XP_007198990.1      NPGVWFMHCHLELHTSWGLKTAFVVENGKDSDHSVLPPPTDLPPC---------------      563
XP_021834477.1      NPGVWFMHCHLELHTSWGLKTAFVVENGKDSDHSILPPPTDLPPC---------------      506
XP_017179926.1      NPGVWFIHCHLELHTGWGLKTAFVVEDGPGSDQSVLPPPKDLPPC---------------      582
XP_021833685.1      NPGVWFMHCHLELHTGWGLKTAFVVEDGPGSDQSVLPVPKDLPPC---------------      564
XP_021833693.1      NPGVWFMHCHLELHTGWGLKTAFVVEDGPGSDQSVLPVPKDLPPC---------------      585
XP_021833835.1      NPGVWFMHCHLELHTGWGLKTAFVVEDGPGSDHSVLPPPKDLPPC---------------      564
XP_008246156.1      ------------------------------------------------------------      219
XP_021833014.1      NPGIWFLHCHLEVHTTWGLKMAFLVENGKGPNETLRPPPADLPTC---------------      558
XP_021814580.1      NPGVWFLHCHLEVHTTWGLKMAFLVDNGKGPNESVLPPPSDLPKC---------------      559
XP_021820658.1      NPGVWFMHCHLEVHTTWGLKMAFVVDNGKGPNESVLPPPSDLPKC---------------      558

XP_021819117.1      ------------------------------------------------------------      565
XP_021833316.1      ------------------------------------------------------------      564
XP_021827255.1      ------------------------------------------------------------      569
XP_021819124.1      ------------------------------------------------------------      569
XP_007201720.2      KLCSTKSALTVNESFPGPIIRAQKGDTVYVNVYNEGRYGVTLHWHGIKQPRNPWFDGPEY      642
XP_021819123.1      ------------------------------------------------------------      570
XP_021826148.1      ------------------------------------------------------------      575
XP_007227301.1      ------------------------------------------------------------      568
XP_021809566.1      ------------------------------------------------------------      474
XP_021816142.1      ------------------------------------------------------------      567
XP_020426263.1      ------------------------------------------------------------      542
XP_021820472.1      ------------------------------------------------------------      568
XP_007200191.1      ------------------------------------------------------------      568
XP_021824778.1      ------------------------------------------------------------      583
XP_021834606.1      ------------------------------------------------------------      563
XP_021829543.1      ------------------------------------------------------------      563
XP_021815052.1      ------------------------------------------------------------      574
XP_021803024.1      ------------------------------------------------------------      582
XP_021825503.1      ------------------------------------------------------------      586
XP_021814279.1      ------------------------------------------------------------      587
XP_021809222.1      ------------------------------------------------------------      583
XP_021809160.1      ------------------------------------------------------------      581
XP_021829870.1      ------------------------------------------------------------      576
XP_007198990.1      ------------------------------------------------------------      563
XP_021834477.1      ------------------------------------------------------------      506
XP_017179926.1      ------------------------------------------------------------      582
XP_021833685.1      ------------------------------------------------------------      564
XP_021833693.1      ------------------------------------------------------------      585
XP_021833835.1      ------------------------------------------------------------      564
XP_008246156.1      ------------------------------------------------------------      219
XP_021833014.1      ------------------------------------------------------------      558
XP_021814580.1      ------------------------------------------------------------      559
XP_021820658.1      ------------------------------------------------------------      558
```

```
XP_021819117.1      ------------------------------------------------------------      565
XP_021833316.1      ------------------------------------------------------------      564
XP_021827255.1      ------------------------------------------------------------      569
XP_021819124.1      ------------------------------------------------------------      569
XP_007201720.2      ITQCPIQPGTNFTYQILLSSEEGTLFWHAHSDWSRATVHGAFVILPANGTSYPFPKPDGE      702
XP_021819123.1      ------------------------------------------------------------      570
XP_021826148.1      ------------------------------------------------------------      575
XP_007227301.1      ------------------------------------------------------------      568
XP_021809566.1      ------------------------------------------------------------      474
XP_021816142.1      ------------------------------------------------------------      567
XP_020426263.1      ------------------------------------------------------------      542
XP_021820472.1      ------------------------------------------------------------      568
XP_007200191.1      ------------------------------------------------------------      568
XP_021824778.1      ------------------------------------------------------------      583
XP_021834606.1      ------------------------------------------------------------      563
XP_021829543.1      ------------------------------------------------------------      563
XP_021815052.1      ------------------------------------------------------------      574
XP_021803024.1      ------------------------------------------------------------      582
XP_021825503.1      ------------------------------------------------------------      586
XP_021814279.1      ------------------------------------------------------------      587
XP_021809222.1      ------------------------------------------------------------      583
XP_021809160.1      ------------------------------------------------------------      581
XP_021829870.1      ------------------------------------------------------------      576
XP_007198990.1      ------------------------------------------------------------      563
XP_021834477.1      ------------------------------------------------------------      506
XP_017179926.1      ------------------------------------------------------------      582
XP_021833685.1      ------------------------------------------------------------      564
XP_021833693.1      ------------------------------------------------------------      585
XP_021833835.1      ------------------------------------------------------------      564
XP_008246156.1      ------------------------------------------------------------      219
XP_021833014.1      ------------------------------------------------------------      558
XP_021814580.1      ------------------------------------------------------------      559
XP_021820658.1      ------------------------------------------------------------      558

XP_021819117.1      ------------------------------------------------------------      565
XP_021833316.1      ------------------------------------------------------------      564
XP_021827255.1      ------------------------------------------------------------      569
XP_021819124.1      ------------------------------------------------------------      569
XP_007201720.2      QVILFASWYKEDVMTLLDETLKSGGLTTSSDSYTINGEPGDFYLCSNETTYRMSVDYGKT      762
XP_021819123.1      ------------------------------------------------------------      570
XP_021826148.1      ------------------------------------------------------------      575
XP_007227301.1      ------------------------------------------------------------      568
XP_021809566.1      ------------------------------------------------------------      474
XP_021816142.1      ------------------------------------------------------------      567
XP_020426263.1      ------------------------------------------------------------      542
XP_021820472.1      ------------------------------------------------------------      568
XP_007200191.1      ------------------------------------------------------------      568
XP_021824778.1      ------------------------------------------------------------      583
XP_021834606.1      ------------------------------------------------------------      563
XP_021829543.1      ------------------------------------------------------------      563
XP_021815052.1      ------------------------------------------------------------      574
XP_021803024.1      ------------------------------------------------------------      582
XP_021825503.1      ------------------------------------------------------------      586
XP_021814279.1      ------------------------------------------------------------      587
XP_021809222.1      ------------------------------------------------------------      583
XP_021809160.1      ------------------------------------------------------------      581
XP_021829870.1      ------------------------------------------------------------      576
XP_007198990.1      ------------------------------------------------------------      563
XP_021834477.1      ------------------------------------------------------------      506
XP_017179926.1      ------------------------------------------------------------      582
XP_021833685.1      ------------------------------------------------------------      564
XP_021833693.1      ------------------------------------------------------------      585
XP_021833835.1      ------------------------------------------------------------      564
XP_008246156.1      ------------------------------------------------------------      219
XP_021833014.1      ------------------------------------------------------------      558
XP_021814580.1      ------------------------------------------------------------      559
XP_021820658.1      ------------------------------------------------------------      558
```

```
XP_021819117.1      ------------------------------------------------------------      565
XP_021833316.1      ------------------------------------------------------------      564
XP_021827255.1      ------------------------------------------------------------      569
XP_021819124.1      ------------------------------------------------------------      569
XP_007201720.2      YLLRIVNSVQNVDMFFAIADHNLTVVGADGAYVKPIVTSYIMITPGQTMDVLVTAKQSLG      822
XP_021819123.1      ------------------------------------------------------------      570
XP_021826148.1      ------------------------------------------------------------      575
XP_007227301.1      ------------------------------------------------------------      568
XP_021809566.1      ------------------------------------------------------------      474
XP_021816142.1      ------------------------------------------------------------      567
XP_020426263.1      ------------------------------------------------------------      542
XP_021820472.1      ------------------------------------------------------------      568
XP_007200191.1      ------------------------------------------------------------      568
XP_021824778.1      ------------------------------------------------------------      583
XP_021834606.1      ------------------------------------------------------------      563
XP_021829543.1      ------------------------------------------------------------      563
XP_021815052.1      ------------------------------------------------------------      574
XP_021803024.1      ------------------------------------------------------------      582
XP_021825503.1      ------------------------------------------------------------      586
XP_021814279.1      ------------------------------------------------------------      587
XP_021809222.1      ------------------------------------------------------------      583
XP_021809160.1      ------------------------------------------------------------      581
XP_021829870.1      ------------------------------------------------------------      576
XP_007198990.1      ------------------------------------------------------------      563
XP_021834477.1      ------------------------------------------------------------      506
XP_017179926.1      ------------------------------------------------------------      582
XP_021833685.1      ------------------------------------------------------------      564
XP_021833693.1      ------------------------------------------------------------      585
XP_021833835.1      ------------------------------------------------------------      564
XP_008246156.1      ------------------------------------------------------------      219
XP_021833014.1      ------------------------------------------------------------      558
XP_021814580.1      ------------------------------------------------------------      559
XP_021820658.1      ------------------------------------------------------------      558

XP_021819117.1      ------------------------------------------------------------      565
XP_021833316.1      ------------------------------------------------------------      564
XP_021827255.1      ------------------------------------------------------------      569
XP_021819124.1      ------------------------------------------------------------      569
XP_007201720.2      LYYMLASPYYDGEADDFDKSMASAIFQYNGNYTPPSSPIYPTYIPGYYDIDSARKFVTQF      882
XP_021819123.1      ------------------------------------------------------------      570
XP_021826148.1      ------------------------------------------------------------      575
XP_007227301.1      ------------------------------------------------------------      568
XP_021809566.1      ------------------------------------------------------------      474
XP_021816142.1      ------------------------------------------------------------      567
XP_020426263.1      ------------------------------------------------------------      542
XP_021820472.1      ------------------------------------------------------------      568
XP_007200191.1      ------------------------------------------------------------      568
XP_021824778.1      ------------------------------------------------------------      583
XP_021834606.1      ------------------------------------------------------------      563
XP_021829543.1      ------------------------------------------------------------      563
XP_021815052.1      ------------------------------------------------------------      574
XP_021803024.1      ------------------------------------------------------------      582
XP_021825503.1      ------------------------------------------------------------      586
XP_021814279.1      ------------------------------------------------------------      587
XP_021809222.1      ------------------------------------------------------------      583
XP_021809160.1      ------------------------------------------------------------      581
XP_021829870.1      ------------------------------------------------------------      576
XP_007198990.1      ------------------------------------------------------------      563
XP_021834477.1      ------------------------------------------------------------      506
XP_017179926.1      ------------------------------------------------------------      582
XP_021833685.1      ------------------------------------------------------------      564
XP_021833693.1      ------------------------------------------------------------      585
XP_021833835.1      ------------------------------------------------------------      564
XP_008246156.1      ------------------------------------------------------------      219
XP_021833014.1      ------------------------------------------------------------      558
XP_021814580.1      ------------------------------------------------------------      559
XP_021820658.1      ------------------------------------------------------------      558
```

```
XP_021819117.1      ------------------------------------------------------------      565
XP_021833316.1      ------------------------------------------------------------      564
XP_021827255.1      ------------------------------------------------------------      569
XP_021819124.1      ------------------------------------------------------------      569
XP_007201720.2      RSLASAEHPVDVPLNVTTRMFITISIGMLHCPNNSCAGPEGNRIASGLNNISFADPAVDV      942
XP_021819123.1      ------------------------------------------------------------      570
XP_021826148.1      ------------------------------------------------------------      575
XP_007227301.1      ------------------------------------------------------------      568
XP_021809566.1      ------------------------------------------------------------      474
XP_021816142.1      ------------------------------------------------------------      567
XP_020426263.1      ------------------------------------------------------------      542
XP_021820472.1      ------------------------------------------------------------      568
XP_007200191.1      ------------------------------------------------------------      568
XP_021824778.1      ------------------------------------------------------------      583
XP_021834606.1      ------------------------------------------------------------      563
XP_021829543.1      ------------------------------------------------------------      563
XP_021815052.1      ------------------------------------------------------------      574
XP_021803024.1      ------------------------------------------------------------      582
XP_021825503.1      ------------------------------------------------------------      586
XP_021814279.1      ------------------------------------------------------------      587
XP_021809222.1      ------------------------------------------------------------      583
XP_021809160.1      ------------------------------------------------------------      581
XP_021829870.1      ------------------------------------------------------------      576
XP_007198990.1      ------------------------------------------------------------      563
XP_021834477.1      ------------------------------------------------------------      506
XP_017179926.1      ------------------------------------------------------------      582
XP_021833685.1      ------------------------------------------------------------      564
XP_021833693.1      ------------------------------------------------------------      585
XP_021833835.1      ------------------------------------------------------------      564
XP_008246156.1      ------------------------------------------------------------      219
XP_021833014.1      ------------------------------------------------------------      558
XP_021814580.1      ------------------------------------------------------------      559
XP_021820658.1      ------------------------------------------------------------      558

XP_021819117.1      ------------------------------------------------------------      565
XP_021833316.1      ------------------------------------------------------------      564
XP_021827255.1      ------------------------------------------------------------      569
XP_021819124.1      ------------------------------------------------------------      569
XP_007201720.2      LQAYYRNISGYYDASFPDEPPNLFNFTAEDLTTDNYTITSRATRVKMLDYNATVEIIFQG      1002
XP_021819123.1      ------------------------------------------------------------      570
XP_021826148.1      ------------------------------------------------------------      575
XP_007227301.1      ------------------------------------------------------------      568
XP_021809566.1      ------------------------------------------------------------      474
XP_021816142.1      ------------------------------------------------------------      567
XP_020426263.1      ------------------------------------------------------------      542
XP_021820472.1      ------------------------------------------------------------      568
XP_007200191.1      ------------------------------------------------------------      568
XP_021824778.1      ------------------------------------------------------------      583
XP_021834606.1      ------------------------------------------------------------      563
XP_021829543.1      ------------------------------------------------------------      563
XP_021815052.1      ------------------------------------------------------------      574
XP_021803024.1      ------------------------------------------------------------      582
XP_021825503.1      ------------------------------------------------------------      586
XP_021814279.1      ------------------------------------------------------------      587
XP_021809222.1      ------------------------------------------------------------      583
XP_021809160.1      ------------------------------------------------------------      581
XP_021829870.1      ------------------------------------------------------------      576
XP_007198990.1      ------------------------------------------------------------      563
XP_021834477.1      ------------------------------------------------------------      506
XP_017179926.1      ------------------------------------------------------------      582
XP_021833685.1      ------------------------------------------------------------      564
XP_021833693.1      ------------------------------------------------------------      585
XP_021833835.1      ------------------------------------------------------------      564
XP_008246156.1      ------------------------------------------------------------      219
XP_021833014.1      ------------------------------------------------------------      558
XP_021814580.1      ------------------------------------------------------------      559
XP_021820658.1      ------------------------------------------------------------      558
```

```
XP_021819117.1      ------------------------------------------------------------      565
XP_021833316.1      ------------------------------------------------------------      564
XP_021827255.1      ------------------------------------------------------------      569
XP_021819124.1      ------------------------------------------------------------      569
XP_007201720.2      TNIMDSGENHPVHLHGFRFYVIGSGLGNFNNVTDPLTYNLVDPPEVNTFPVPKDGWATIR      1062
XP_021819123.1      ------------------------------------------------------------      570
XP_021826148.1      ------------------------------------------------------------      575
XP_007227301.1      ------------------------------------------------------------      568
XP_021809566.1      ------------------------------------------------------------      474
XP_021816142.1      ------------------------------------------------------------      567
XP_020426263.1      ------------------------------------------------------------      542
XP_021820472.1      ------------------------------------------------------------      568
XP_007200191.1      ------------------------------------------------------------      568
XP_021824778.1      ------------------------------------------------------------      583
XP_021834606.1      ------------------------------------------------------------      563
XP_021829543.1      ------------------------------------------------------------      563
XP_021815052.1      ------------------------------------------------------------      574
XP_021803024.1      ------------------------------------------------------------      582
XP_021825503.1      ------------------------------------------------------------      586
XP_021814279.1      ------------------------------------------------------------      587
XP_021809222.1      ------------------------------------------------------------      583
XP_021809160.1      ------------------------------------------------------------      581
XP_021829870.1      ------------------------------------------------------------      576
XP_007198990.1      ------------------------------------------------------------      563
XP_021834477.1      ------------------------------------------------------------      506
XP_017179926.1      ------------------------------------------------------------      582
XP_021833685.1      ------------------------------------------------------------      564
XP_021833693.1      ------------------------------------------------------------      585
XP_021833835.1      ------------------------------------------------------------      564
XP_008246156.1      ------------------------------------------------------------      219
XP_021833014.1      ------------------------------------------------------------      558
XP_021814580.1      ------------------------------------------------------------      559
XP_021820658.1      ------------------------------------------------------------      558

XP_021819117.1      ------------------------------------------------------------      565
XP_021833316.1      ------------------------------------------------------------      564
XP_021827255.1      ------------------------------------------------------------      569
XP_021819124.1      ------------------------------------------------------------      569
XP_007201720.2      FIANNPGVWFMHCHFDRHMSWGMDTVFIVKNGGTNETSIRPPPDYLPACSKNSLFGADQS      1122
XP_021819123.1      ------------------------------------------------------------      570
XP_021826148.1      ------------------------------------------------------------      575
XP_007227301.1      ------------------------------------------------------------      568
XP_021809566.1      ------------------------------------------------------------      474
XP_021816142.1      ------------------------------------------------------------      567
XP_020426263.1      ------------------------------------------------------------      542
XP_021820472.1      ------------------------------------------------------------      568
XP_007200191.1      ------------------------------------------------------------      568
XP_021824778.1      ------------------------------------------------------------      583
XP_021834606.1      ------------------------------------------------------------      563
XP_021829543.1      ------------------------------------------------------------      563
XP_021815052.1      ------------------------------------------------------------      574
XP_021803024.1      ------------------------------------------------------------      582
XP_021825503.1      ------------------------------------------------------------      586
XP_021814279.1      ------------------------------------------------------------      587
XP_021809222.1      ------------------------------------------------------------      583
XP_021809160.1      ------------------------------------------------------------      581
XP_021829870.1      ------------------------------------------------------------      576
XP_007198990.1      ------------------------------------------------------------      563
XP_021834477.1      ------------------------------------------------------------      506
XP_017179926.1      ------------------------------------------------------------      582
XP_021833685.1      ------------------------------------------------------------      564
XP_021833693.1      ------------------------------------------------------------      585
XP_021833835.1      ------------------------------------------------------------      564
XP_008246156.1      ------------------------------------------------------------      219
XP_021833014.1      ------------------------------------------------------------      558
XP_021814580.1      ------------------------------------------------------------      559
XP_021820658.1      ------------------------------------------------------------      558
```

```
XP_021819117.1      ------   565
XP_021833316.1      ------   564
XP_021827255.1      ------   569
XP_021819124.1      ------   569
XP_007201720.2      MLQMGE   1128
XP_021819123.1      ------   570
XP_021826148.1      ------   575
XP_007227301.1      ------   568
XP_021809566.1      ------   474
XP_021816142.1      ------   567
XP_020426263.1      ------   542
XP_021820472.1      ------   568
XP_007200191.1      ------   568
XP_021824778.1      ------   583
XP_021834606.1      ------   563
XP_021829543.1      ------   563
XP_021815052.1      ------   574
XP_021803024.1      ------   582
XP_021825503.1      ------   586
XP_021814279.1      ------   587
XP_021809222.1      ------   583
XP_021809160.1      ------   581
XP_021829870.1      ------   576
XP_007198990.1      ------   563
XP_021834477.1      ------   506
XP_017179926.1      ------   582
XP_021833685.1      ------   564
XP_021833693.1      ------   585
XP_021833835.1      ------   564
XP_008246156.1      ------   219
XP_021833014.1      ------   558
XP_021814580.1      ------   559
XP_021820658.1      ------   558
```

Figure S1: Alignment of the sweet cherry LMCOs showing the conserved L1 (green box), L3 (cyan box), M2 (purple box) and M4 (underlined) motifs[20].

a

	1	2	3	4	5	6	7
1: Lac-Ma	100.00	30.54	25.83	22.33	22.94	22.43	21.88
2: Lac-Tt	30.54	100.00	29.00	28.45	27.85	27.14	26.69
3: AO-Cp	25.83	29.00	100.00	31.14	29.07	30.97	30.54
4: LMCO-Arabidopsis	22.33	28.45	31.14	100.00	49.64	50.28	53.78
5: LMCO-Litchi	22.94	27.85	29.07	49.64	100.00	59.96	61.03
6: Lac-Rhus	22.43	27.14	30.97	50.28	59.96	100.00	62.29
7: LMCO-Cherry	21.88	26.69	30.54	53.78	61.03	62.29	100.00

b

```
Lac-Ma              EPTCNTPSNRACWSDGFDINTDY--EVSTPDTGVTQSYVFNLTEVDNWMGPDGVVKEKVM   58
Lac-Tt              ------------------------------AIGP--VADLTISNG--AVSP-DGFSRQAI   25
Ao-Cp               --------------------------------SQIRHYKWEVEYM--FWAP-NCNENIVM   25
LMCO-Arabidopsis    ------------MSHSFFNLFLISL--FLYNNCIAHHYTFTVREV--PYTK-LCSTKAIL   43
LMCO-Litchi         ----------MGLVSRFLRLMFLGCLLFCEAEGAVHYYDFVVKES--NFTR-LCNTKSML   47
Lac-Rhus            --------------------------------VDVHNYTFVLQEK--NFTK-WCSTKSML   25
LMCO-Cherry         ----MGKYNNMSWVLAFVGFMLLGG--ICKANGAIYNYDFVLREE--NFTK-LCTTKSIL   51
                                                           :               .  :

Lac-Ma              LINGNIMGPNIVANWGDTVEVTVINN----LVTNGTSIHWHGIHQKDTNLHDGANGVTEC   114
Lac-Tt              LVNDVFPSPLITGNKGDRFQLNVIDNMTNHTMLKSTSIHWHGFFQHGTNWADGPAFVNQC   85
Ao-Cp               GINGQFPGPTIRANAGDSVVVELTNK----LHTEGVVIHWHGILQRGTPWADGTASISQC   81
LMCO-Arabidopsis    TVNSQFPGPIIKVHKGDTIYVNVQNR-----ASENITMHWHGVEQPRNPWSDGPEYITQC   98
LMCO-Litchi         TVNDSFPGPEIRVQKGDTAFVTVYNQ-----GPYGITIHWHGVKMPRNPWSDGPEYVTQC   102
Lac-Rhus            VVNGSFPGPTITARKGDTIFVNVINQ-----GKYGLTIHWHGVKQPRNPWSDGPEYITQC   80
LMCO-Cherry         TVNGSFPGPTITVRKGDTAFVNVHNQ-----GLYGLTIHWHGVKNPRNPWSDGPENITQC   106
                     :*. : .* *  . **   : : :.        .  :****.    .    **   :.:*
```

```
Lac-Ma              PIPPKGGQRTYRWRARQYGTSWYHSHFSAQYGNGVVGTIQINGPA---SLPYDIDLGVF-   170
Lac-Tt              PISTGHAFLYDFQVPDQAGTFWYHSHLSTQYCDGLRGPIVVYDPQDPHKSLYDVDDDSTV   145
Ao-Cp               AINPGETFFYNFTVD-NPGTFFYHGHLGMQRSAGLYGSLIVDPPQGKK-EPFH-YDGEIN   138
LMCO-Arabidopsis    PIRPGSDFLYKVIFSIEDTTVWWHAHSSWTR-ATVHGLIFVYPRPPQI-LPFPKADHEVP   156
LMCO-Litchi         KIAPRTNFTQEINFSIEEGTIWWHAHSDWSR-ATVHGAIIVYPASGTT-YPYPTPDGEQT   160
Lac-Rhus            PIKPGTNFIYEVILSTEEGTLWWHAHSDWTR-ATVHGALVILPANGTT-YPFPPPYQEQT   138
LMCO-Cherry         PIPAGTNFTHEVIFSSEEGTLWWHAHSDWTR-ATVHGAIIILPALNTS-YPFVTPDAQET   164
                     *              :  * ::*.* .      : * : :          :

Lac-Ma              -PITDYYYRAADDLVHFT------QNNAP-------------PFSDNVLINGTAVN-PNT   209
Lac-Tt              ITLADWYHLAAKVG-------------SP------------VPTADATLING-LGRSIDT   179
Ao-Cp               LLLSDWWHQSIHKQEVGLSSKPIRWIGEPQTILLNGRGQFDCSIAAKYDSNLEPCKLKGS   198
LMCO-Arabidopsis    IILGEWWKRDVREVVEEFV----RTGGAP-------------NVSDALTINGHPGFLYPC   199
LMCO-Litchi         IVLASWYNSDVMEVYEEAV----ASGEEF-------------NTSDAFTINGQPGALYDC   203
Lac-Rhus            IVLASWFKGDVMEVITSSE----ETGVFP-------------AAADGFTINGELGDLYNC   181
LMCO-Cherry         LILGSWFKGDVNEIIEEAL----ATGGDP-------------NISDAFTINGEPGDLYDC   207
                      : .::                                       :     *

                                         #
Lac-Ma              GEGQYANVTLTPGKRHRLRILNTSTENHFQVSLVNHTMTVIAADMVPVNAMTVDSLFLAV   269
Lac-Tt              LNADLAVITVTKGKRYRFRLVSLSCDPNHVFSIDGHSLTVIEADSVNLKPQTVDSIQIFA   239
Ao-Cp               ESCAPYIFHVSPKKTYRIRIASTTALAALNFAIGNHQLLVVEADGNYVQPFYTSDIDIYS   258
LMCO-Arabidopsis    SKSDTFHLTVEKGKTYRIRMVNAAMNLPLFFAIANHSLTVVSADGHYIKPIKATYITISP   259
LMCO-Litchi         STGTTFRMNVTSGKTYLLRIINAILNEEMFFGIANHNLTVVGTDGFYTKPINAEYIFITP   263
Lac-Rhus            SKETTYRLSVQPNKTYLLRIVNAVLNEEKFFGIAKHTLTVVAQDASYIKPINTSYIMITP   241
LMCO-Cherry         SNETTYRWLVDYGKTYLLRVINAVMNEEHFFAIANHNLTVVAQDAAYIKPITTSYVMITP   267
                             :   * : :*: .        ..:   * : *:   *     :    .   : :

Lac-Ma              GQRYDVVIDASRAPD-NYWFNVTFGGQAACGGSLNPHPAAIFHYAGAPGGLPTDEGTPPV   328
Lac-Tt              AQRYSFVLNADQDVG-NYWIRALPNS-G-TRNFDGGVNSAILRYDGAAPVEPTT-----S   291
Ao-Cp               GESYSVLITTDQNPSENYWVSVGTRARH-P---NTPPGLTLLNYLPNSVSKLP--TSPPP   312
LMCO-Arabidopsis    GETLDMLLHADQDPERTYYMAARAYQSG-NIDFNNSTTIGILSYTSSCKAKTSSFSGYYP   318
LMCO-Litchi         GQTIDVLVTANQTPS-YYYMAASPFSDS-EADFDNSTTVAYFQYIGNETVPDP-DPIPFP   320
Lac-Rhus            GQTMDVLFTTDQTPS-HYYMVASPFHDA-LDTFANFSTNAIIQYNGSYKAP---KSPFVK   296
LMCO-Cherry         GQTMDILVTANQSPS-HYYIASTSFVDG-DVAFNNSTTTAILQYNGNYSAP---STIPLP   322
                    .:  ..:. :.:     *:.                        :  *

Lac-Ma              D----------------HQCLDTLDVRPVVPRSVPVNSFVKRPDNTLPVALDL----TGT   368
Lac-Tt              QTPSTNPLVES-----ALTTLEGTAAPGS-PAPGGVDL----ALNMA----------FGF   331
Ao-Cp               QTPAWDDFDRSKNFTYRITAAMGS---PKPPVKFNRRIFLLNTQNVIN------------   357
LMCO-Arabidopsis    TLPFYNDTSAAFGFFTKIKCLFSG----QVPVQISRRIITTVSINLRMCPQNSCEGPNG-   373
LMCO-Litchi         SLPGTNDSGPPFGFIRQLRSLADA-AXVSVPTNIPKHIYMTASVNVIYCPNDSCSANLGG   379
Lac-Rhus            PLPVYNDIKAADKFTGKLRSLANEKFPVNVPKVNVRRIFMAVSLNIVKCANKSCNNNIG-   355
LMCO-Cherry         NLPDHDDDTAAANFTTQVRALASEEHPISVPVNISHRLFITVSINERICPNSSCDGPDN-   381
                                       *              *

Lac-Ma              PLFVWKVNGSDINVDWGKPIIDYILTGN-----TSYP----------------------V   401
Lac-Tt              AGGKFTINGASFTPPT-VPVLLQILSGAQSAQD------------------------LL   365
Ao-Cp               GYVKWAINDVSLALPP-TPYLGAMKYNLLHAFDQNPPPEVFPEDY---DIDTPPTNEKTR   413
LMCO-Arabidopsis    SRLAASMNNISFVTPSHVDILKAYYYHIKGVYGTRFP-EFPPLIFNFTAENQPLFLETPR   432
LMCO-Litchi         DKLGASLNNQSFQFPS-IDILQAYYNNISGVFTTDFP-LEPPSFFNFTAVEPNVT-VYAG   436
Lac-Rhus            HSTSASLNNISFALPQ-TDVLQAYYRNISGVFGRDFP-TVQKK--------ANFS-LNTA   404
LMCO-Cherry         NALAASLNNISFVTPS-IDILQAYYGGINGVYTPNFP-DKPPYFFNFTGLVQNNT-IYPS   438
                          :*. .:          :

Lac-Ma              -SDNIVQVDAVDQWTYWLI-ENDPEGPFSLPHPMHLHGHDFLVLGRSPDVPAASQQRFVF   459
Lac-Tt              PSGSVYSLPANADIEISLPATAA---APGFPHPFHLHGHTFAVVRSA-------------   409
Ao-Cp               IGNGVYQFKIGEVVDVILQNANMMKENLSETHPWHLHGHDFWVLGYGDGKFSA-------   466
LMCO-Arabidopsis    LATEVKVIEFGQVVELVIQGTSLVGG--GLDHPMHLHGFSFYVVGVGFGNYNI-------   483
LMCO-Litchi         QGTKVIELDYGDEVELVFQGTNL-GN--AQNHPMHLHGYSFYMVGLGFGNFNS-------   486
Lac-Rhus            QGTQVLMIEYGEAVEIVYQGTNL-GA--ATSHPMHLHGFNFYLVGTGAGTFNN-------   454
LMCO-Cherry         FATKVKMIKYGKEVEIIFQGTSM-IA--AENHPMHLHGFSFYLVGTGSGNFNS-------   488
                     .  :  .                    .   ** ****. * ::   .
                                                             #
```

```
Lac-Ma               DPAVDLARLNGDNPPRRDTTMLP-AGGWLLLAFRTDNPGAWLFHCHIAWHVSGGLSVDFL   518
Lac-Tt               ----GSSTYNYENPVYRDVVSTGSPGDNVTIRFRTDNPGPWFLHCHIDFHLEAGFAVVMA   465
Ao-Cp                EEE---SSLNLKNPPLRNTVVIF-PYGWTAIRFVADNPGVWAFHCHIEPHLHMGMGVVFA   522
LMCO-Arabidopsis     SEEDPSSRYNLYDPPYKNTMTVP-RNGWIAIRFVADNPGVWFMHCHLDRHQTWGMNVVFI   542
LMCO-Litchi          STD--PSSYNLVDPPEVNTIQLS-KRGWVAIRFKADNPGVWLMHCHYERHTAWGMAAVFI   543
Lac-Rhus             VTD--PPKYNLVDPPELNTINLP-RIGWAAIRFVADNPGVWFLHCHFERHTTEGMATVVI   511
LMCO-Cherry          TTS--PKTYNLVDPPEVNTIGVP-KKGWAAIRFVADNPGVWYMHCHLERHSSWGMNTVLI   545
                              *  :*   :.       .   : * :**** * :***   *   *: . .

Lac-Ma               ERPADLRQRISQEDEDDFNRVCDEWRAYWPTNPYPKIDSGL    559
Lac-Tt               EDIPEVAATNPV-PQ-AWSDLCPTYDA-LSPDDQ-------    496
Ao-Cp                EGVEKVG------RIPTKALACGGTAKSLINNPKNP-----    552
LMCO-Arabidopsis     VKNGREPNQQIL-PPPDDLPPCYE-----------------    565
LMCO-Litchi          VKDGGTTNTSMR-APPAYMPSCSSS----------------    567
Lac-Rhus             VKDGGTTNTSML-PSPAYMPPCS------------------    533
LMCO-Cherry          VRNGNTTETSIL-PPPAYMPPCSKS----------------    569
                                           *
```

Figure S2: Multiple alignment and identity matrix of LMCO and ascorbate oxidase (Ao) from plants and fungi. **a:** percent identity matrix. **b:** copper-binding motifs. Green highlight, L1 region; purple highlight, M2 region; underlined, M4 region; cyan highlight, L3 region. In fungal sequences, the L2 region described in Giardina and colleagues[39] is shown in pink. Red residues depict copper-binding residues to T1, T2 and T3. Boxed, substrate-binding loops; grey, loop I; yellow, loop II; green, loop III and blue, loop IV. Some other conserved residues characteristic of plant sequences are shown in white with black background. White amino acids, residues interacting with the phenolic substrates within the active-site. Orange, axial residues within the active-site that are involved in the redox potential. #, residues involved in catalysis (blue) and electron abstraction from the substrate (red His).

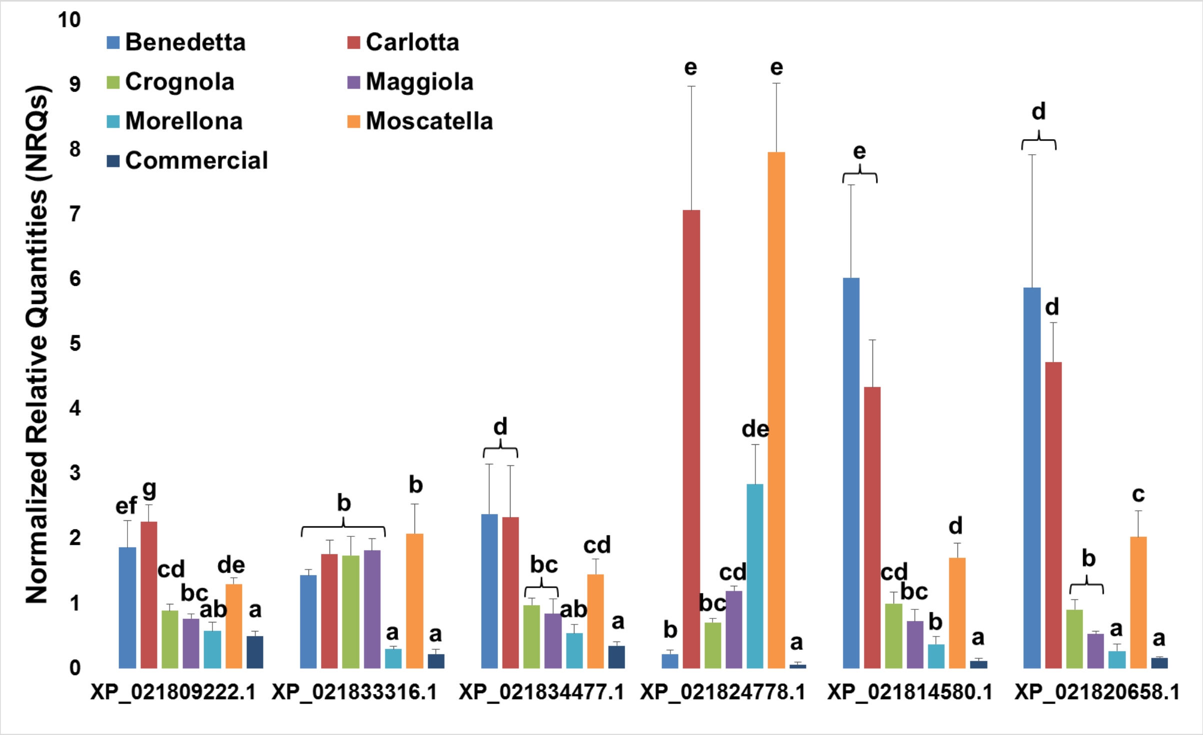

Figure S3: Gene expression analysis of the six LMCOs in the ancient and commercial sweet cherry varieties. The accession numbers indicate the proteins encoded by the corresponding genes. Error bars indicate standard deviations

(n=4). Different letters indicate statistically significant differences (p<0.05) calculated with the Tukey's post-hoc test.
